# Evaluation of Imidazolium Ionenes: Solid–Solid Phase Change Materials as Heat Sinks

**DOI:** 10.3390/polym17131782

**Published:** 2025-06-27

**Authors:** Carolina Arriaza-Echanes, Gabriel I. Krüger, Bibiana Comesaña-Gándara, Claudio A. Terraza, Loreto Sanhueza, Pablo A. Ortiz

**Affiliations:** 1 Centro de Nanotecnología Aplicada, Facultad de Ciencias, Ingeniería y Tecnología, Universidad Mayor, Camino La Pirámide 5750, Huechuraba 8580745, Chile; carolina.arriaza@mayor.cl; 2 Departamento de Ingeniería Química, Universidad Católica del Norte, Antofagasta 1240000, Chile; g.krugercarrasco@gmail.com; 3 IU CINQUIMA, University of Valladolid, Paseo Belén 5, 47011 Valladolid, Spain; bibiana.comesana@uva.es; 4 Research Laboratory for Organic Polymers (RLOP), Department of Organic Chemistry, Pontificia Universidad Católica de Chile, Santiago 7820436, Chile; cterraza@uc.cl; 5 Núcleo de Química y Bioquímica, Facultad de Ciencias, Ingeniería y Tecnología, Universidad Mayor, Camino La Pirámide 5750, Huechuraba 8580745, Chile; loreto.sanhueza@umayor.cl; 6 Escuela de Ingeniería en Medio Ambiente y Sustentabilidad, Facultad de Ciencias, Ingeniería y Tecnología, Universidad Mayor, Camino La Pirámide 5750, Huechuraba 8580745, Chile

**Keywords:** polyelectrolytes, ionenes, heat sink, SS-PCMs

## Abstract

Overheating in miniaturized electronic devices can reduce their useful life, where conventional heat sinks are insufficient. The utilization of ionenes as solid–solid phase change materials is proposed to enhance thermal dissipation without the risk of leakage. In this work, a series of imidazolium ionenes with structural modifications in their aromatic core and aliphatic chain length were synthesized. The synthesis was carried out using the respective monomers diimidazole and alkyl dibromide, followed by counterion bromide exchange using lithium bis(trifluoromethanesulfonyl)imide, with yields over 90% in all cases. Thermal characterizations showed that all ionenes are heat-resistant, with degradation temperatures between 421 °C and 432 °C; moreover, they all presented only a solid–solid transition (Tg) as a phase change, between 59 °C and 28 °C, which varied depending on the aromatic core used and the length of the aliphatic chain. The obtained ionenes were introduced into an experimental device with an operating temperature of 40 °C, to be evaluated as solid–solid phase change materials in heat sinks. These demonstrated an average decrease in operating temperature of 9 °C compared to the device without ionenes. On the other hand, the stability of the ionenes was analyzed over 10 thermal cycles at 40 °C at a heating rate of 5 °C/min. This analysis demonstrated that the ionenes did not present changes or degradation during the evaluated cycles. These findings demonstrate that imidazolium ionenes are promising solid–solid phase change materials for use as efficient and self-repairing heat sinks in compact electronic devices.

## 1. Introduction

Nowadays, electronic devices are undergoing a constant process of miniaturization and an increase in their operating power [1,2]. Consequently, there is a significant increase in the amount of heat produced during their operation, which in turn leads to an increase in the temperature of the device. In some cases, this temperature exceeds its optimal operating point, thereby affecting the performance, reliability, and lifetime of various components of the device, such as CPUs, GPUs, LEDs, and batteries, among others [2,3]. In addition, the high temperatures generated accelerate device failures, causing irreversible damage leading to device loss [4]. For this reason, thermal management becomes a crucial point, especially when working with electronic devices that generate a large amount of heat, such as CPUs, GPUs, servers, and data centers [5].

Thermal management in electronic devices has traditionally used passive heat sinks, which do not require external energy and dissipate heat through convection or radiation due to their large surface area [6]. Additionally, active systems such as fans can be employed to enhance dissipation [7].

The materials most commonly used as passive heat sinks are copper or aluminum [8]. In addition, polymers have become important for thermal management. Exploration of the design of passive dissipation devices, such as flexible pulsating heat pipes [9], or by protecting substrates in high-temperature manufacturing processes, such as during sintering for flexible solar cells [10], has been conducted.

However, these heat sinks are insufficient or inapplicable in miniaturized devices [5,11]. Passive heat sinks are unable to handle the temperatures generated [12], while active systems add complexity, size, power consumption, and noise [13,14]. Space constraints have driven the search for new strategies and materials for optimal thermal management [15,16].

A promising alternative is phase change materials (PCMs) [17]. PCMs store and release heat during a phase transition (latent heat) [18,19,20], offering better thermal management within a specific temperature range and acting as an effective buffer against heat spikes [21,22]. Integrated into a heat sink, PCMs absorb excess heat during operating cycles, reducing the temperature and keeping it more stable [23,24,25]. The stored heat is released during low-power or idle cycles, preventing the device from overheating [18,26].

Currently, PCMs are classified by their composition (organic, inorganic, and eutectic) [27,28] and by the phase changes observed (solid–liquid, liquid–gas, and solid–solid) [29,30]. Solid–liquid phase change PCMs (SL-PCMs) are the most studied due to their high enthalpy (latent heat) [31,32]. However, their transition to the liquid phase has the disadvantage of increasing volume and the risk of leakage, which requires the material to be encapsulated; this adds complexity to their application [33,34,35,36].

Considering these limitations, solid–solid phase change materials (SS-PCMs) have recently received much attention [37,38,39]. These materials feature a transition between two solid phases, their main advantage being a more stable phase, eliminating the risk of leakage and the need for encapsulation [40,41]. This simplifies PCM incorporation, improves thermal contact, and reduces device complexity. In addition, these materials possess lower volume variation and higher mechanical stability than SL-PCMs [37,42].

Of the many existing materials, polymers have been widely used as PCMs [43,44,45]. Poly(ethylene)glycol (PEG) is one of the main polymers under study due to its high latent heat (melting) enthalpy, low cost, and wide range of melting temperatures, which can be adjusted according to its molecular weight [46,47]. However, despite its characteristics, its phase transition is a melting process, so it passes through a liquid state during the operating temperature. It also has low conductivity and thermal stability [48,49]. Research indicates that utilizing PEG in conjunction with materials capable of containing it can prevent leaks and enhance thermal stability. This approach results in a composite that functions akin to an SS-PCM [44,50,51].

One potential solution to address the limitations of conventional polymers, such as PEG, involves the use of polyelectrolytes (PEs) as PCMs. Polyelectrolytes are defined as polymers that contain ionic groups within their structure, either in the main chain (ionenes) or in the side chains (ionomers). Polyelectrolytes with a melting temperature below 100 °C are designated as poly(ionic liquid)s [52,53]. As with non-ionic polymers, ionenos have been observed to exhibit a glass transition temperature, whereby a phase change occurs from a crystalline to a rubbery state. This property renders them suitable for utilization as solid–solid phase change materials (SS-PCM) [54]. The primary benefit of polyelectrolytes as SS-PCMs is their enhanced thermal stability, attributable to the presence of ionic groups, which enables them to endure a greater number of heating and cooling cycles [55,56,57]. Furthermore, ionenes with imidazolium, amide, and imide groups [58,59] have exhibited the capacity for self-healing, a feature that is advantageous in their utilization as PCMs in scenarios where replacement of components is not feasible.

A characteristic of these materials is that, being polymers, structural modifications can be made to modulate their thermal properties, such as modifying the type of cation or anion, the spacing between charges, and the stiffness and flexibility of the chain, among others [52,60].

Among the cations used for PEs, phosphonium, pyridinium, and imidazolium stand out [61,62,63]. The latter are cations formed from imidazole, and their main advantages over others are their low cost and simplicity of synthesis [64,65,66]. As for anions, the most common are halides formed during PE synthesis; however, modification of counterion can be performed by exchange with anions such as tetrafluoroborate, hexafluorophosphate, and bis(trifluoromethanesulfonyl)imide, among others. It is important to note that the properties of PE may be subject to alteration, depending on the type of counterion employed [67,68].

In the present work, the synthesis and characterization of imidazolium ionenes with variations in the length of the aliphatic hydrocarbon chain and in their aromatic core have been developed. The aim of these structural modifications is to modulate their thermal properties to obtain efficient solid–solid phase change materials (SS-PCMs). The obtained SS-PCMs were incorporated into an experimental device and evaluated as heat sinks.

## 2. Materials and Methods

### 2.1. Materials and Characterization Techniques

Reagents and solvents were purchased from Sigma Aldrich-Merck (San Francisco, CA, USA), except lithium bis(trifluoromethane)sulfonimide (LiNTf_2_), which was obtained from AK Scientific Inc. (Union City, CA, USA): 1-(3-aminopropyl)imidazole (API); pyromellitic dianhydride (PMDA); biphenyl-tetracarboxylic acid dianhydride (BPDA); 4,4′-oxydiphthalic anhydride (ODPA); 1,4-dibromobutane (C_4_); 1,8-dibromooctane (C_8_); 1,12-dibromododecane (C_12_); *N*,*N*-dimethylformamide (DMF); dimethyl sulfoxide (DMSO); ethyl ether; acetonitrile; ethanol; methanol; acetone; ethyl acetate; chloroform; and tetrahydrofuran (THF).

### 2.2. Synthesis of Diimidazole Monomers and Ionenes

The synthesis, purification, and characterization of the diimidazole monomers were performed as described in Arriaza-Echanes et al., 2023 [69]. For the synthesis of the monomers (Figure S1), 70 mmol of the corresponding dianhydride (PMDA, BPDA, or ODPA), 147 mmol of 1-(3-aminopropyl)imidazole (API, 5% mol excess), and 45 mL of DMF were added to a round bottom flask, heating at 130 °C for 24 h.

**Figure 1:**
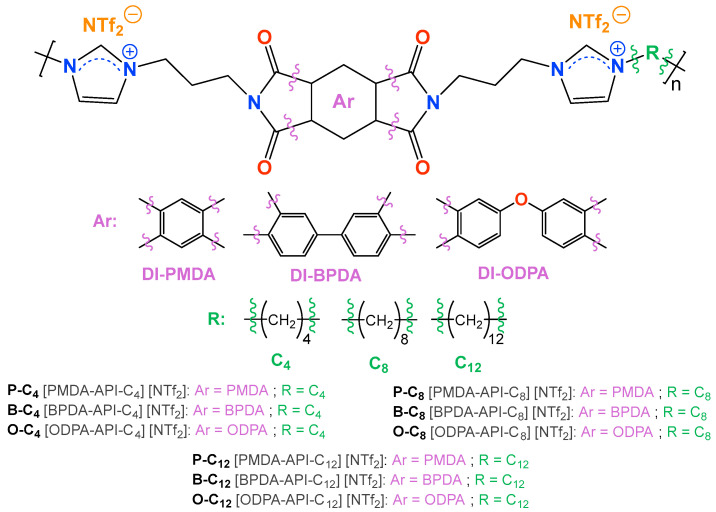
Structure of the synthesized ionenes grouped according to the dibromide monomer used.

Three series of ionenes were prepared [69] (Figure 1). For this purpose, 4.0 mmol of diimidazole monomer (DI-PMDA, DI-OPDA, or DI-BPDA), 4.0 mmol of dibromide derivative (C_4_, C_8_, or C_12_), and 20 mL of DMF were added to a high pressure tube (60 mL), which was sealed and left for 24 h at 100 °C under stirring. Then, 8.4 mmol of lithium bis(trifluoromethanesulfonyl)imide (LiNTf_2_, 5% excess) was added, and the reaction was again left for 24 h at 100 °C. After this time, the reaction mixture was dropped into 200 mL of distilled water, causing precipitation of the ionene. Subsequently, the solid obtained was isolated by filtration, washed thoroughly with water, and dried at 60 °C. Ionenes were purified by reprecipitated, dissolving them in acetonitrile and adding water dropwise (Figure S2).

### 2.3. Characterization

FTIR-ATR (ZnSe) spectra were recorded on a Spectrum Two (Perkin Elmer, Waltham, MA, USA) spectrophotometer over the range of 450–4000 cm^−1^. ^1^H, ^13^C, ^19^F, DEPT-135°, COSY, HSQC, and HMBC NMR spectra were carried out on a 500 MHz instrument Bruker Avance (Bruker Corporation, Karlsruhe, Germany) using DMSO-*d*_6_ as solvent and TMS as internal standard. For the solubility tests, 50 mg of ionene and 0.5 mL of the solvent were added to a glass test tube and shaken for five minutes at room temperature. The crystallinity of the polymers was determined by polarized light microscopy, using a Motic BA310Pol microscope equipped with a 20X optical objective. The ionenes were deposited on a glass slide and heated to 100 °C for 5 min, then allowed to cool and observed under the microscope. Additionally, the ionenes were prepared by the solvent-casting method, dissolving 100 mg in 1 mL of acetone, and allowed to dry at room temperature and observed under the microscope. Thermal stability of the ionenes was evaluated using a TGA-50 SHIMADZU thermogravimetric analyzer (Shimadzu, Columbia, MD, USA). Analysis was performed using a temperature range of 30–800 °C, under nitrogen atmosphere, and a heating ramp of 20 °C/min; the maximum degradation speed temperatures (T_d_) were determined from the peaks of the first derivative of the TGA curve (DTGA). The glass transition temperature (Tg) of the ionenes was determined using a TA Instruments DSC Q20 differential scanning calorimeter (Hüllhorst, Germany). The measurements were from −50 to 250 °C at a speed of 10 °C/min, with a nitrogen atmosphere with a flow of 50 mL/min. The results were analyzed using TRIOS software 5.7 from TA Instruments, OriginPro 8.5, and MestReNova 14.2. The self-healing test was performed by thermoforming 1 g of ionenes into a square shape (1.5 × 1.5 cm, thickness of 2.6–3.3 mm), cutting it, and heating it on a Teflon plate at 100 °C for 1, 5, and 10 min to evaluate the cut repair.

### 2.4. Evaluation of Ionenes as Heat Sinks

**Figure 2:**
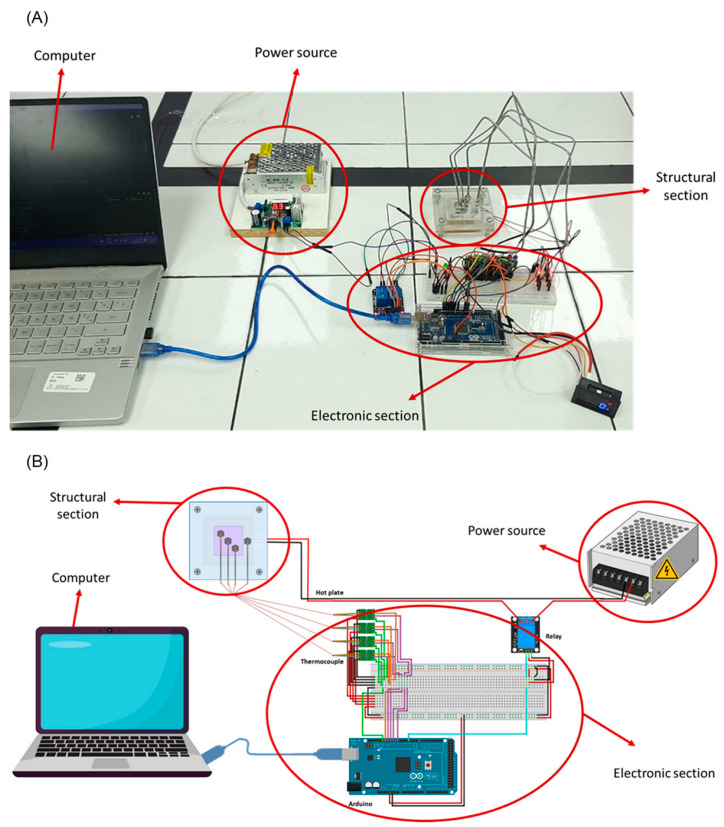
Experimental device for testing ionenes as heat sink materials. (**A**) Photograph and (**B**) illustration of the experimental device.

To evaluate the performance of the ionenes as heat sinks, an experimental device was constructed as shown in Figure 2. This device consisted of two sections: a structural section to contain and thermally insulate the ionenes and an electronic section for temperature control and measurement and data collection.

**Figure 3:**
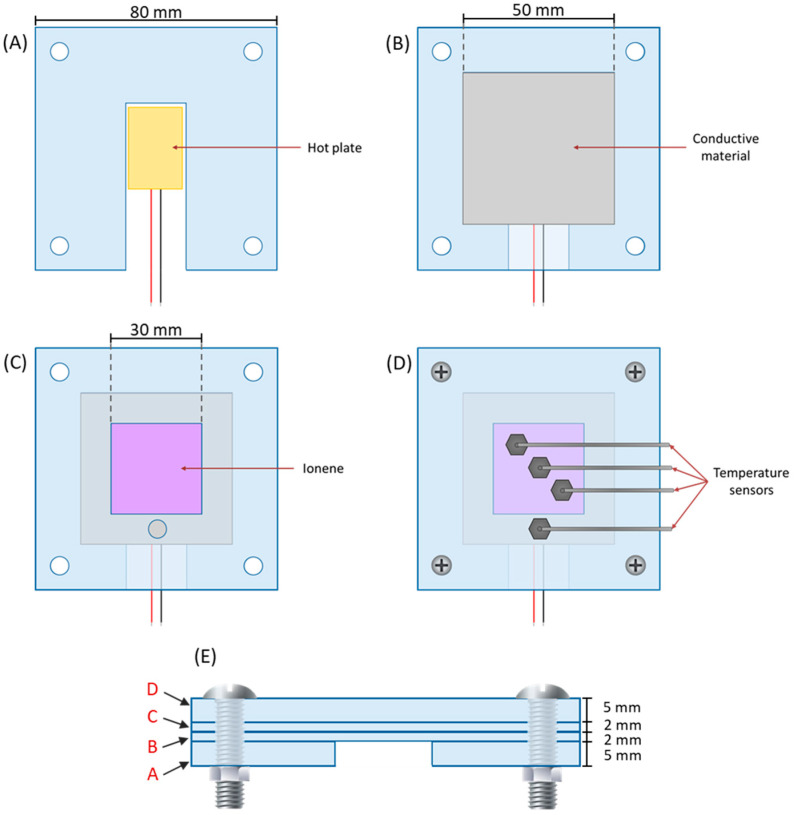
Schematic of the experimental device setup. (**A**–**D**) These correspond to the parts that form the structural section of the experimental device and (**E**) This corresponds to the assembly of the structural section of the experimental device, indicating the order of the parts (**A**–**D**).

The structural section of the device was fabricated from four acrylic panels (insulating material) and an AA1100XX aluminium plate (conductive material). Figure 3 shows the diagram of the dimensions of each structural panel. Furthermore, it can be observed that the structure has a space to deposit the material to be analyzed, which is in direct contact with the aluminum plate during the evaluation (Figure 3C).

An Arduino Mega 2560 microcontroller (Arduino, Ivrea, Italy) was responsible for temperature data acquisition, reading time recording, and runtime control. Temperature measurement was performed with three type K-type thermocouples with a measurement range between 0 to 800 °C and an accuracy of ±0.1 °C, and one type K-type thermocouple as a control. A 12W PTC heating plate, capable of reaching a maximum temperature of 120 °C, was used as the heat source. Additionally, an adjustable LM317 power supply (28 V; 2 A/220 V) was integrated for the operation of the heating plate.

The specifications and technical diagrams of the experimental system are found in Figures S3 and S4. The code used for Arduino is available on GitHub https://github.com/KrugerGK/SS-PCMs-heatsink.

Once the experimental system was assembled, 2.5 g of each ionene was dimensioned as a 3 × 3 cm square section, and these were deposited in the experimental system. The measurements were carried out in two stages: In the first, the sample was heated until a constant temperature of 40 °C was achieved for a period of 30 min (1800 s), with the aim of simulating an electronic device in operation. Subsequently, in the second stage, the heat supply was suspended, allowing the system to cool naturally for 60 min (3600 s). All measurements were performed in triplicate. This procedure allows the evaluation of heat sink performance under controlled conditions.

## 3. Results and Discussion

### 3.1. Synthesis and Structural Characterization of Ionenes

During the synthesis, it was observed that increasing the length of the aliphatic chain length of the dibromide used increased the solubility of the ionenes formed in the reaction medium. Thus, ionenes with aliphatic chains of 8 or 12 carbon atoms were soluble in the reaction medium, while those with four methylene units partially precipitated. This fact was attributed to the spacing of the ionic (cationic) groups in the main chain, which decreases the ionic character of the chain, favoring dipolar interactions between the material and the solvent. Finally, the ionenes were isolated and purified, with a synthesis yield of 91% to 97% and a purification yield of 89% to 93%. This latter process allowed for the removal of shorter chains formed during the polymerization.

**Figure 4:**
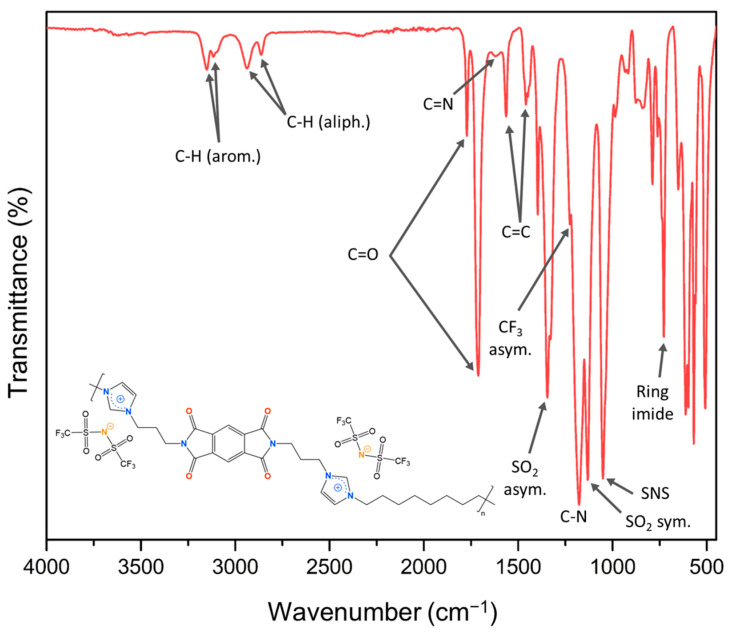
FT-IR spectrum of P-C_8_ ([PMDA-API-C8] [NTf_2_]).

Structural characterization of the purified ionenes was performed by FT-IR and NMR (^1^H, ^13^C, ^19^F, DEPT-135°, COSY, HSQC, and HMBC) spectroscopy, and the spectra recorded are shown in Figures S5–S13. As an example, Figure 4 shows the FT-IR spectrum of the P-C_8_, indicating the main signals observed. In all FT-IR spectra, the bands corresponding to C-H stretching of sp^3^ and sp^2^ carbon atoms are identified between 3150 and 2800 cm^−1^, the carbonyl atoms signals of the imide group (C=O) between 1770 and 1710 cm^−1^, the C=N signal of imidazolium groups at 1450 cm^−1^, the C-N bonds belonging to the imide and imidazolium group at 1390 and 1170 cm^−1^, and the imide ring substitution between 728 and 740 cm^−1^. Along with the aforementioned signals, the asymmetric and symmetric stretching bands of the SO_2_ groups were observed at approximately 1345 cm^−1^ and 1130 cm^−1^, respectively, a signal corresponding to the asymmetric stretching of the CF_3_ unit at 1220 cm^−1^, and finally, at 1050 cm^−1^, the signal corresponding to the S-N-S moiety. These latter signals refer to the presence of the bis(trifluoromethanesulfonyl)imide (NTf_2_) counterion exchanged for the respective bromide ions during the second part of the ionene synthesis.

**Figure 5:**
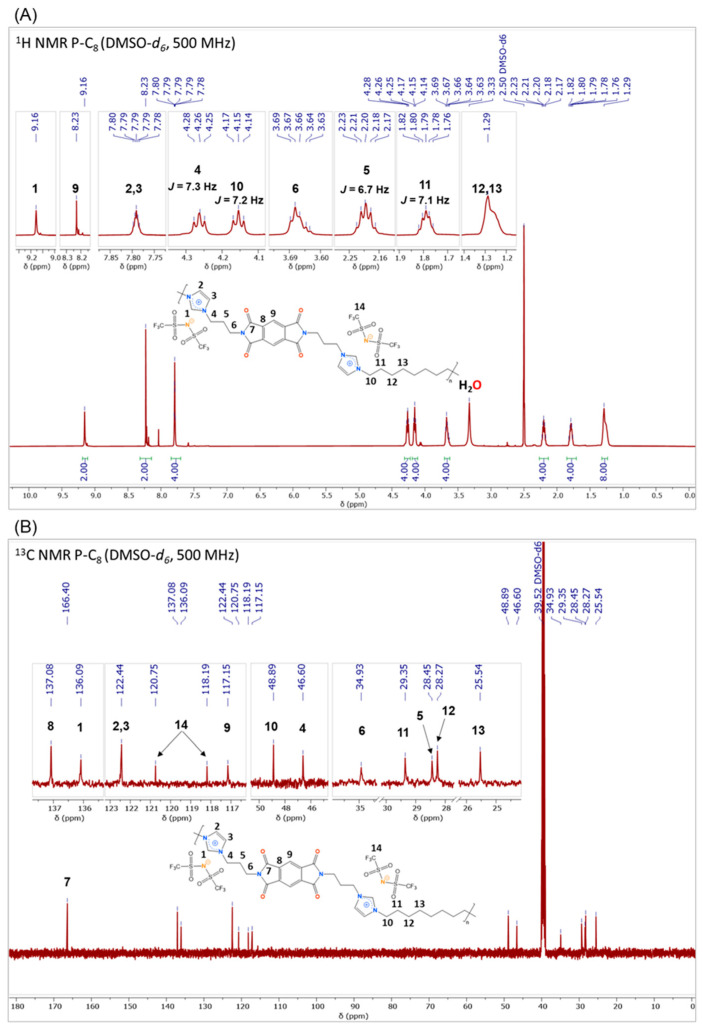
NMR spectra of P-C_8_ ([PMDA-API-C8] [NTf_2_]) in DMSO-*d*_6_. (**A**) ^1^H and (**B**) ^13^C.

All ^1^H NMR spectra of the ionenes showed a singlet near 9.1 ppm, which integrates for 2H. This signal, shifted downfield relative to the other observed signals, was consistent with the deshielding effect of the imidazolium nitrogen atoms on the methylene group located between them (H1). Signals centered between 8.3 ppm and 7.5 ppm were assigned to the aromatic hydrogen atoms of the arylimide core and the imidazolium ring. In addition, between 4.3 and 1.2 ppm, signals corresponding to the aliphatic hydrogens of each ionene were observed, which were assigned using both ^1^H spectra and COSY, HMBC, and HSQC spectra. Noteworthy here is the presence of two triplet signals shifted further downfield than the rest of these signals. These signals were attributed to the hydrogen atoms adjacent to the imidazolium group, which strongly deshields them. Figure 5A shows the ^1^H NMR spectrum of P-C_8_ as an example.

In the case of the ^13^C NMR spectra, all the expected signals for the non-equivalent carbon atoms present in the respective ionenes were observed and, similar to what was observed in the ^1^H NMR spectra, the difference between the aromatic and aliphatic signals was appreciated. Furthermore, at low field, the carbonyl carbons of the arylimide core stood out with a shift close to 167 ppm. Aromatic carbon atoms were visualized between 144 ppm and 113 ppm and, in the case of aliphatic nuclei, between 48 ppm and 25 ppm. Moreover, among the signals of the aromatic carbon, two signals (120 ppm and 118 ppm) were observed, which were assigned as the carbon atoms of the NTf_2_ counterion. Figure 5B shows the ^13^C NMR spectrum of P-C_8_ as an example.

Finally, the presence of a fluorine atom from the NTf_2_ counterion was confirmed by ^19^F NMR analysis. In the resulting spectra, a signal near −78.7 ppm was observed, which corroborated the presence of the CF_3_ unit and that the counterion exchange process was effective.

### 3.2. Properties of Ionenes

#### 3.2.1. Solubility

Solubility tests were carried out with the solvents described in Table 1 to determine which solvents are suitable for the characterization and processing of ionenes. From these results, it can be seen that the samples are soluble in aprotic solvents of high polarity, such as DMSO, DMF, acetonitrile, and also in acetone. Meanwhile, they remained insoluble in protic polar solvents such as water, ethanol, and methanol, and in low polarity solvents such as THF, ethyl acetate, chloroform, and ethyl ether.

These results show significant progress in the structure–solubility relationship of this type of material, as they are soluble in highly volatile solvents, such as acetone, a low-cost reagent that enables their processing through techniques such as solvent casting. This property is particularly promising for future applications such as additive manufacturing, inkjet printing, or spray coating, where the use of a volatile, low-cost solvent is crucial for depositing thin, uniform layers of material on heat sinks or other electronic components, especially when compared to solvents such as DMSO and DMF. Importantly, solubility was reduced in protic polar solvents, which are often used to dissolve materials with similar characteristics, such as ionic liquids or other ionenes [70], especially when they have small counterions such as bromides or chlorides [68,71]. This fact is consistent with the hydrophobic characteristics and size of the NTf_2_ counterion, which neutralizes the imidazolium cation while blocking its interaction with the hydroxyl groups of protic polar solvents. As for the performance of the low polarity solvents, it was expected that they would not be able to dissolve this series of ionenes due to the significant difference in their interactions. In this sense, while ionenes have groups that confer dipolar, ionic, and charge transfer complexes interactions, these solvents have mainly low polarity dipolar and London dispersion interactions.

#### 3.2.2. Crystalline Structure

The crystallinity of the ionenes was observed using polarized light microscopy. In Figure S14, the photographs obtained for each ionene were recorded both with and without polarized light. It was determined that when the ionenes were exposed to polarized light, the resulting images appeared black or dark. This finding indicates that all ionenes were predominantly amorphous materials. This allows us to infer that the synthesized ionenes could undergo a solid–solid phase transition, also known as a glass transition. As they do not exhibit significant regularity (crystallinity), it is less likely that they will undergo a solid–liquid transition (melting).

**Figure 6:**
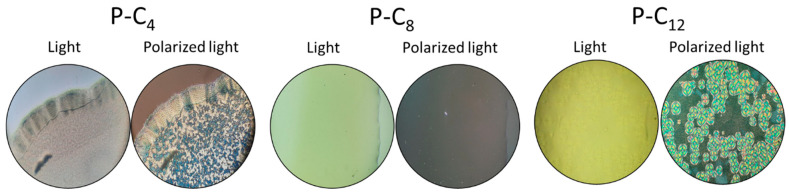
Evaluation of the crystalline structure of P-series ionenes using solvent casting.

The formation of crystalline structures in a material was influenced by various factors, including its molecular structure, as well as parameters such as time, temperature, concentration, pressure, and the method of processing [72,73]. For this reason, the possibility of generating crystalline structures of each ionene using the solvent-casting method was evaluated. The ionenes were dissolved in acetone, then deposited on a glass slide, and finally, the solvent was allowed to evaporate at room temperature. Following desiccation, the ionenes were analyzed once more by means of polarized light microscopy. It was determined that only the P-series was capable of forming crystalline structures (Figure 6).

It is believed that the aromatic core influences the ability of the P-series ionenes to form crystalline structures. As the smallest of the three studied, the aromatic core of this series may cause the chains to extend to avoid charge repulsion. Furthermore, the aromatic core was conducive to π–π stacking due to its rigid, flat structure, which exhibits a lower degree of freedom and consequently generates greater order. This suggests that these materials have the ability to form crystalline structures under defined conditions, which could be of interest for other applications.

#### 3.2.3. Thermal Properties

**Figure 7:**
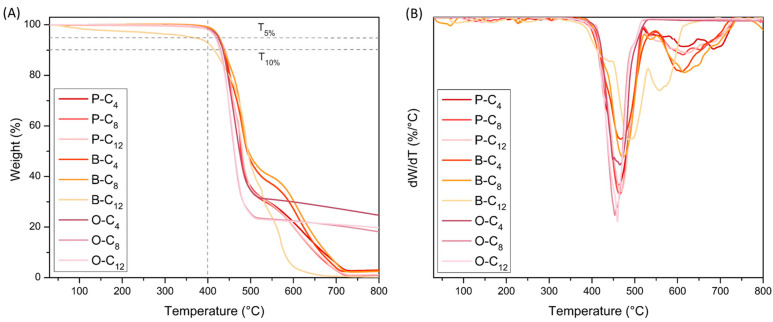
Thermograms of the ionenes. (**A**) TGA and (**B**) DTGA.

Thermal stability was determined by thermogravimetric analysis in a nitrogen atmosphere, as shown in Figure 7A. The main parameters recorded from these analyses are summarized in Table 2. From these analyses, all the synthesized ionenes were determined to be thermoresistant with onset decomposition temperature (T_onset_) between 421 °C and 432 °C, while the weight loss of 5% and 10% of the materials occurs between 365–427 °C and 415–440 °C, respectively. Furthermore, the temperature of the maximum degradation rate (T_d1_) of ionenes was found to be between 453 and 491 °C. Furthermore, it was noted that the B- and P-series ionenes exhibited a secondary maximum degradation temperature (T_d2_). This process can be linked to the degradation of the aromatic core. While the ionenes of the O-series do not exhibit T_d2_, it is possible that the degradation of the aromatic core is occurring at a temperature close to T_d1_. This breakdown is favored by the presence of oxygen in the diphenylether group (Figure 7B). From these results, it was observed that, despite structural modifications in the nature of the aromatic core or the progressive increase in aliphatic length chain promoted by the dibrominated compounds, the ionenes did not show significant differences in their decomposition temperatures. This fact shows that the thermoresistance of these materials can be attributed to the presence of charged or ionic groups, such as the imidazolium cation and the NTf_2_ anion, which provide strong ionic interactions that absorb much of the heat applied during the analysis.

Despite the non-oxidizing atmosphere used in the analysis, the residue values at 800 °C (R_800_) for the PMDA- and BPDA-derived ionene series are low (<3%), whereas the polymers formed from ODPA show substantially higher values (~20–25%). This could be explained by the incorporation of the C-O-C bond, which increases the polarity of the aromatic core and incorporates a higher C-O bond energy (351 KJ/mol) with respect to C-C (347 KJ/mol) [74]. While the diphenylether group may be favoring the initial rupture of the aromatic core, as mentioned in the observations on the degradation temperature, the higher energy required for bond rupture could be generating non-volatile residues at the temperature used in the analysis. In future work, the formation of these non-volatile groups in the residues will be determined using techniques such as GS mass, EDS, and XRD, among others, to determine the nature of these compounds. This information will facilitate the proposal of structures with better thermal resistance.

The thermal resistance results were of great importance for the application of these ionenes as SS-PCMs in heat sinks incorporated into electronic devices, given that these heat sinks usually operate at temperatures not exceeding 100 °C. Moreover, the small variation in these values opens up the possibility of modulating other thermal properties through structural modifications while maintaining their high thermal stability.

**Figure 8:**
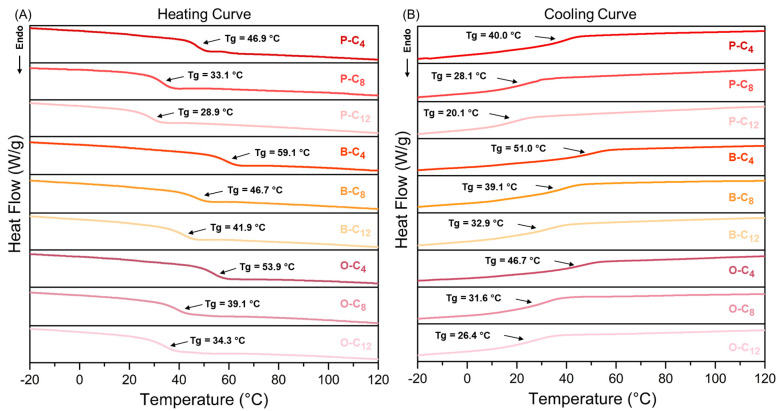
Heating and cooling curves obtained from the DSC analysis of the ionenes. (**A**) Heating curve and (**B**) Cooling curve.

In order to use these ionenes as SS-PCMs, it is necessary to determine the presence of a solid–solid transition, in this case, a glass transition. For this reason, all ionenes were analyzed by differential scanning calorimetry (DSC). Figure 8 shows the heating and cooling curves of all ionenes, where it was observed that all ionenes have a glass transition temperature (Tg) and no melting or crystallization point in the temperature range studied (−50 °C to 250 °C). Modification of the aromatic core, by the incorporation of a second ring, should result in biphenyl o diphenylether moieties, increasing the aromatic content in the polymer chain, causing the P-series ionenes to exhibit a lower Tg compared to the B- and O-series (Table 3). When comparing these last two series of ionenes, a difference in Tg was observed; B-series exhibits higher values compared to O-series. This is due to the higher stiffness and lower number of degrees of freedom presented by the biphenyl moiety in contrast to the aromatic rings linked by the oxyether function.

When considering the effect of the aliphatic chain contributed by the dibromide monomer on Tg, it was observed that there is a decrease in this parameter with increasing methylene units. This is due to the incorporation of flexible moieties with more degrees of freedom and to the increase in the spacing between charges within the chains. Furthermore, regardless of the aromatic core of the ionenes, it was observed that increasing the aliphatic chain length by 4 carbon atoms (from 4 to 8) produces an average decrease in Tg of 13 °C, while a further increase in the chain from 8 to 12 carbon atoms produced an average decrease in Tg value of 4.5 °C.

Given the way in which Tg decreased in relation to the increase in aliphatic chain length, it can be inferred that the contribution of these chains decreases drastically after eight carbon atoms. This phenomenon can be attributed to the fact that the initial increase in carbon atoms (from C4 to C8) causes a significant disruption in the packing of the chains and increases the free volume, resulting in a decrease in Tg. In contrast, the change from 8 to 12 carbons exerts a comparatively minor influence on the mobility of the polymer chain, resulting in a stabilization of the Tg, which is evident as a reduced decrease in the Tg value. This effect is comparable to that observed in other polymeric materials, in which the increase in chain length does not vary their thermal properties since they cease to depend on length and depend only on the type of group and its chemical properties [75,76]. In addition, the supercooling temperature of the ionenes was also determined, with an average value of 7.5 °C between the heating and cooling Tg. This variation is close to the values sought for materials applied in heat dissipation, which present a supercooling of 5 °C, being paraffins (pure or mixtures) generally used for this application [77].

As for the glass transition enthalpies (∆H_Tg_), these did not show large variations with respect to the structural modifications developed, with ∆H_Tg_ values of 3.24 to 5.00 J/g and 2.23 to 4.44 J/g for heating and cooling, respectively. Furthermore, the low values observed, compared to the enthalpies of fusion exhibited by PCMs such as paraffin (200–220 J/g), are mainly due to the difference in the thermal phenomena studied. While Tg uses heat to produce changes or movements in the polymer chain, fusion uses heat to break crystalline domains, thus requiring much more energy (heat). Although this result may seem negative, it actually opens the opportunity to propose other structural modifications that could improve or increase the enthalpy values in the glass transition. Such as the incorporation of longer chains that could favor a certain degree of ordering, which would generate an increase in intermolecular interactions such as London dispersion forces, generating an increase in the enthalpy value. Maintaining a balance between increasing the degree of order without reaching fusion.

**Figure 9:**
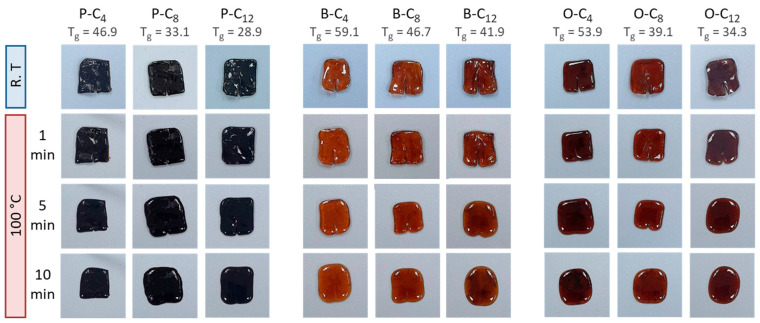
Photographs obtained during the ionene self-healing test.

Along with thermal analyses, self-healing tests were performed on all ionenes. For this purpose, the ionenes were thermoformed into squares, which were then cut longitudinally and subsequently heated on a Teflon plate. Figure 9 shows images of the ionenes with the cut at room temperature and heated at 100 °C for 1, 5, and 10 min. It is observed that all ionenes show some degree of repair after 1 min of heating, either because of the partial decrease in separation generated by the cut or because the size of the cut begins to reduce. Likewise, it is appreciated that the repair of the cut consists of the union of the sections separated by the cut and not by the displacement or flow of the material. Finally, after 10 min of heating, all ionenes were self-repaired. This self-repair capability is attractive for materials inside electronic devices since, in case of breakage of the device by impact, cutting, or vibration, they can self-repair, maintaining their functionality.

### 3.3. Characterization of Ionenes as SS-PCMs in Heat Sinks

To evaluate whether the synthesized ionenes have the ability to act as heat sinks, they were subjected to heating at 40 °C, simulating the maximum operating temperature of a real mobile device, and their responses were compared with those of the device without polymer. The working temperature was determined based on the evaluation temperature of the mobile devices [78]. At temperatures above this, the thermal comfort of the user is exceeded, and manual use is uncomfortable [79,80]. Moreover, this temperature is similar to the average Tg of the series of synthesized ionenes that will act as SS-PCMs.

**Figure 10:**
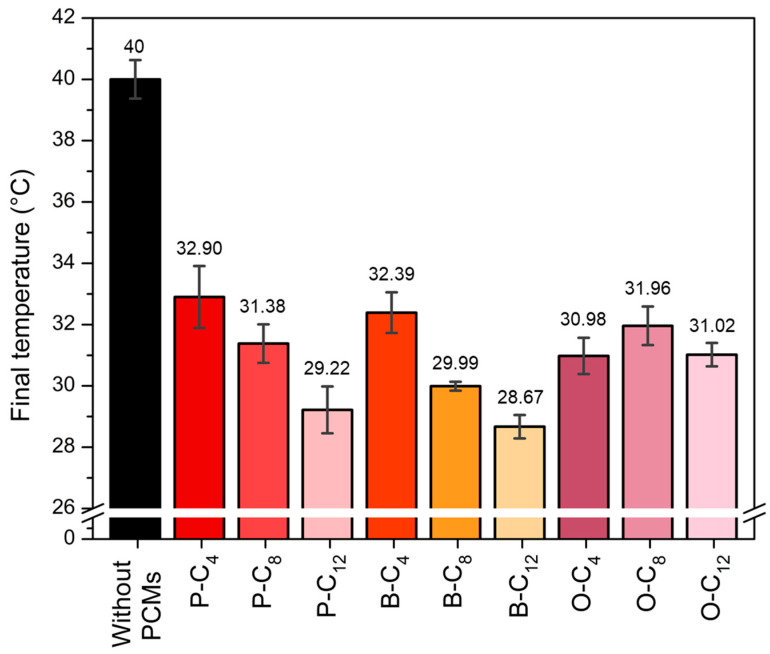
Final operating temperature reached in the device during heating at 40 °C.

The test was performed by heating the experimental device to 40 °C for 30 min and allowing it to cool freely for 1 h. Figure S15 shows the results of the tests performed comparing the heating of the device without PCM (without ionene) versus with PCM (with ionene) over the analysis time, while Figure 10 summarizes the final operating temperature reached by the ionenes after 30 min of heating at 40 °C. The use of ionenes as SS-PCM in the experimental device allowed an average reduction in operating temperature of 9 °C. P-C_4_ was the sample that reduced the temperature the least, reaching 32.90 °C, which corresponds to a decrease of 7.10 °C compared to the device without ionene. On the other hand, the ionene that reduced the temperature the most was B-C_12_, with a final temperature of 28.67 °C and a temperature reduction of 11.33 °C compared to the device without ionene. The observed variation in the temperatures obtained can be attributed to the length of the aliphatic chain. In a chain of 12 carbons, intermolecular interactions are favored, and it is more energy (heat) that is required to break these interactions. Although this decrease may be marginal, it is important to consider that, for electronic devices, decreasing the operating temperature by 1 °C reduces the temperature-related failure rate by 4% [81].

A comparative analysis shows that in the P- and B-series, there is a marked relationship between the length of the aliphatic chain and the maximum operating temperature. In other words, the longer the aliphatic section provided by dibromide monomers, the lower the maximum operating temperature reached by the device. This fact seems to be associated with the increased degrees of freedom of this section, which allows it to absorb more energy (heat), and with the fact that, for these two families, the flexible segment is spaced by a much longer rigid segment. The latter would be contributing to maximizing the effect of the aliphatic segment on its heat absorption capacity. However, in order to corroborate this, it is necessary to extend the family of ionenes studied and incorporate X-ray diffraction analyses that provide additional information on the crystalline domains and the organization of the chains in the solid state.

**Figure 11:**
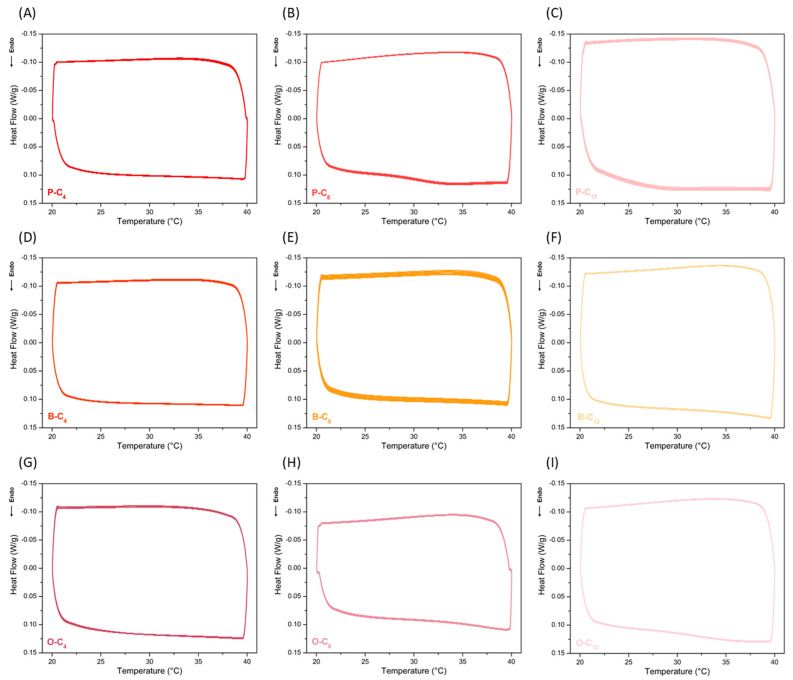
Heat storage–release cycles of the ionenes. Series P (**A**–**C**), series B (**D**–**F**), and series O (**G**–**I**).

The thermal stability of the ionenes was evaluated during storage and heat release cycles, emulating their operation as heat sinks. This was performed by calorimetry (DSC) over a range of 20 °C to 40 °C, the operating temperature interval, with a heating rate of 5 °C/min, under a nitrogen atmosphere, and for 10 heating–cooling cycles. Figure 11 shows the tests performed with all ionenes, in which no significant variations or additional thermal phenomena caused by material degradation or changes in the ionenes during the heat absorption and release processes were observed. This demonstrated the thermal stability of these materials, at least during the cycles evaluated, given that their decomposition temperature is well above their operating temperature. However, it is important to underline that in order to evaluate their long-term behavior, it will be necessary to perform tests with a larger number of cycles.

This study focuses on the use of ionenes as SS-PCMs in thermal management. It is therefore essential to acknowledge the limitations of this study. In order to apply these materials in the field of thermal management, it is essential to conduct long-term studies to evaluate their thermal stability during heating and cooling cycles. Incorporating these materials into a real (commercial) electronic device would allow their behavior to be evaluated under non-simulated conditions. In conjunction with the above, future research will explore in greater detail the self-healing capacity of these materials and the implications for their mechanical properties. X-ray diffraction (XRD) analysis will also be used to make a conclusive correlation between the trends observed in this work regarding the molecular structure of ionenes and their thermal properties.

## 4. Conclusions

Three series of ionenes with variations in aliphatic chain length and aromatic core architecture were obtained from the polymerization of diimidazolium monomers and linear dibrominated compounds of 4, 8, and 12 methylene units. By means of an exchange reaction, the substitution of the bromide counterion by the bis(trifluoromethane)sulfonamide anion was achieved. Furthermore, the synthesized ionenes proved to be soluble in acetone, which is an advantage for their processing, as this is a low-cost solvent compared to DMF and DMSO. The ability to modulate the thermal properties of imidazolium ionenes through specific structural modifications was demonstrated. It was possible to obtain materials with a variety of solid–solid transition temperatures (Tg between 28.9 °C and 59.1 °C) by varying the aromatic core and the length of the aliphatic chain while maintaining their high thermal stability (T_10%_ between 415 °C and 440 °C). In addition, the ability of ionenes to act as SS-PCMs in heat dissipation was evaluated, demonstrating that they can decrease the maximum operating temperature of the device by an average of 9 °C compared to not using ionenes. The ionenes that were employed demonstrated consistent thermal stability across a minimum of 10 heating and cooling cycles. This research demonstrated the potential of ionenes to function as SS-PCMs in the context of heat dissipation for the purpose of thermal management in electronic devices.

## Figures and Tables

**Table 1:** Ionene solubility test results.

Solvent	P-C_4_	P-C_8_	P-C_12_	B-C_4_	B-C_8_	B-C_12_	O-C_4_	O-C_8_	O-C_12_
Water	−	−	−	−	−	−	−	−	−
DMSO	+	+	+	+	+	+	+	+	+
DMF	+	+	+	+	+	+	+	+	+
Acetonitrile	+/−	+	+	+	+	+	+	+	+
Acetone	+/−	+	+	+	+	+	+	+	+
Ethanol	−	−	−	−	−	−	−	−	−
Methanol	−	−	−	−	−	−	−	−	−
Ethyl acetate	−	−	−	−	−	−	−	−	−
THF	−	−	−	−	−	−	−	−	−
Chloroform	−	−	−	−	−	−	−	−	−
Ethyl ether	−	−	−	−	−	−	−	−	−

+: Soluble, −: Insoluble, +/−: Partially soluble.

**Table 2:** Summary of thermal parameters from thermogravimetric analysis.

Ionene	T_onset_ (°C) ^a^	T_5%_ (°C) ^b^	T_10%_ (°C) ^b^	T_d1_ (°C) ^c^	T_d2_ (°C) ^c^	R_800_ (%) ^d^
**P-C_4_**	426	424	436	462	618	2.98
**P-C_8_**	430	426	438	464	614	1.01
**P-C_12_**	432	424	440	468	626	1.18
**B-C_4_**	424	427	438	467	606	2.83
**B-C_8_**	427	427	438	473	618	2.52
**B-C_12_**	421	365	415	491	561	0.01
**O-C_4_**	427	425	435	459	-	24.7
**O-C_8_**	429	419	429	453	-	18.1
**O-C_12_**	428	417	428	459	-	19.8

^a^: Temperature at which degradation of ionene begins. ^b^: Temperature at which 5% and 10% weight is lost, respectively. ^c^: Temperature at which material is degraded in one and/or two stages, respectively. ^d^: Percentage of residual material at 800 °C.

**Table 3:** Main parameters obtained from DSC analysis of the ionenes.

	Heating Curve			Cooling Curve			
Ionene	T_g_ (°C)	Cp (J/g°C)	∆H_Tg_ (J/g)	T_g_ (°C)	Cp (J/g°C)	∆H_Tg_ (J/g)	∆T_g_ (°C)
**P-C_4_**	46.9	0.247	3.74	40.0	0.258	2.89	6.9
**P-C_8_**	33.1	0.248	4.08	28.1	0.319	3.63	5.0
**P-C_12_**	28.9	0.229	3.42	20.1	0.317	2.41	8.8
**B-C_4_**	59.1	0.222	4.11	51.0	0.220	2.99	8.1
**B-C_8_**	46.7	0.249	3.99	39.1	0.299	3.39	7.6
**B-C_12_**	41.9	0.281	5.00	32.9	0.362	4.44	9.0
**O-C_4_**	53.9	0.243	3.24	46.7	0.247	2.23	7.2
**O-C_8_**	39.1	0.307	3.87	31.6	0.341	3.37	7.5
**O-C_12_**	34.3	0.294	4.51	26.4	0.333	3.28	7.9

Tg: glass transition temperature. Cp: specific heat capacity. ∆H_Tg_: enthalpy glass transition. ∆Tg: difference between Tg obtained by heating and cooling (supercooling temperature).

## Data Availability

The original contributions presented in this study are included in the article/Supplementary Materials. Further inquiries can be directed to the corresponding author.

## Supplementary Materials

The following supporting information can be downloaded at: https://www.mdpi.com/article/10.3390/polym17131782/s1, Figure S1: Synthesis scheme for obtaining diimidazole monomers; Figure S2: Synthesis scheme of the ionenes; Figure S3: Dimensions of the structural part of the experimental setup; Figure S4: Connection diagram of the electronic section; Figure S5: FT-IR-ATR and 1H, 13C, DEPT 135°, COSY, HSQC, HMBC, and 19F NMR spectra of ionene P-C4; Figure S6: FT-IR-ATR and 1H, 13C, DEPT 135°, COSY, HSQC, HMBC, and 19F NMR spectra of ionene P-C8; Figure S7: FT-IR-ATR and 1H, 13C, DEPT 45°, COSY, HSQC, HMBC, and 19F NMR spectra of ionene P-C12; Figure S8: FT-IR-ATR and 1H, 13C, DEPT 135°, COSY, HSQC, HMBC, and 19F NMR spectra of ionene B-C4; Figure S9: FT-IR-ATR and 1H, 13C, DEPT 45°, COSY, HSQC, HMBC, and 19F NMR spectra of ionene B-C8; Figure S10: FT-IR-ATR and 1H, 13C, DEPT 135°, COSY, HSQC, HMBC, and 19F NMR spectra of ionene B-C12; Figure S11: FT-IR-ATR and 1H, 13C, DEPT 135°, COSY, HSQC, HMBC, and 19F NMR spectra of ionene O-C4; Figure S12: FT-IR-ATR and 1H, 13C, DEPT 45°, COSY, HSQC, HMBC, and 19F NMR spectra of ionene O-C8; Figure S13: FT-IR-ATR and 1H, 13C, DEPT 45°, COSY, HSQC, HMBC, and 19F NMR spectra of ionene O-C12; Figure S14: Evaluation of the crystalline structure of ionenes using polarized light microscopy; Figure S15: Results of the evaluation of the ionenes as SS-PCMs in heat dissipation.
