# Supplementary material for: Evaluation of Imidazolium Ionenes: Solid–Solid Phase Change Materials as Heat Sinks
Source: Polymers (Basel). 2025 Jun 27;17(13):1782. doi: 10.3390/polym17131782 (PMC12252075; doi:10.3390/polym17131782)
Supplement: Supplementary file 1 [file polymers-17-01782-s001.zip › polymers-3712253-supplementary.pdf]

Evaluation of imidazolium ionenes: Solid-Solid Phase Change Materials as heat sinks

Carolina Arriaza-Echanes[1], Gabriel I. Krüger[2], Bibiana Comesaña-Gándara[3], Claudio A. Terraza[4], Loreto Sanhueza[5] and Pablo A. Ortiz[1,6*]

[1] Centro de Nanotecnología Aplicada, Facultad de Ciencias, Ingeniería y Tecnología, Universidad Mayor, Camino La Pirámide 5750, 8580745, Huechuraba, Santiago, RM, Chile.

[2] Departamento de Ingeniería Química, Universidad Católica del Norte, 12900, Antofagasta, Chile.

[3] IU CINQUIMA, University of Valladolid, Paseo Belén 5, 47011, Valladolid, Spain.

[4] Research Laboratory for Organic Polymers (RLOP), Department of Organic Chemistry, Pontificia Universidad Católica de Chile, 7820436, Santiago, RM, Chile.

[5] Núcleo de Química y Bioquímica, Facultad de Ciencias, Ingeniería y Tecnología, Universidad Mayor, Camino La Pirámide 5750, 8580745, Huechuraba, Santiago, RM, Chile.

[6] Escuela de Ingeniería en Medio Ambiente y Sustentabilidad, Facultad de Ciencias, Ingeniería y Tecnología, Universidad Mayor, Camino La Pirámide 5750, 8580745, Huechuraba, Santiago, RM, Chile.

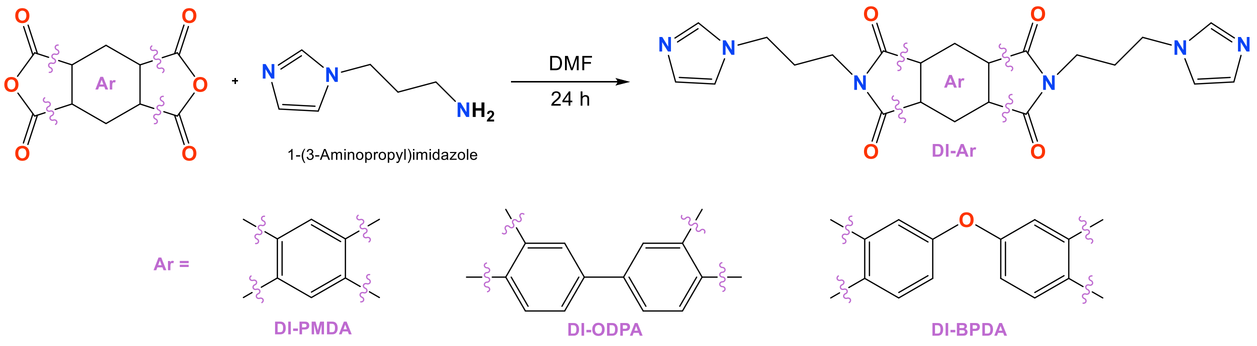

Figure S1: Synthesis scheme for obtaining diimidazole monomers.

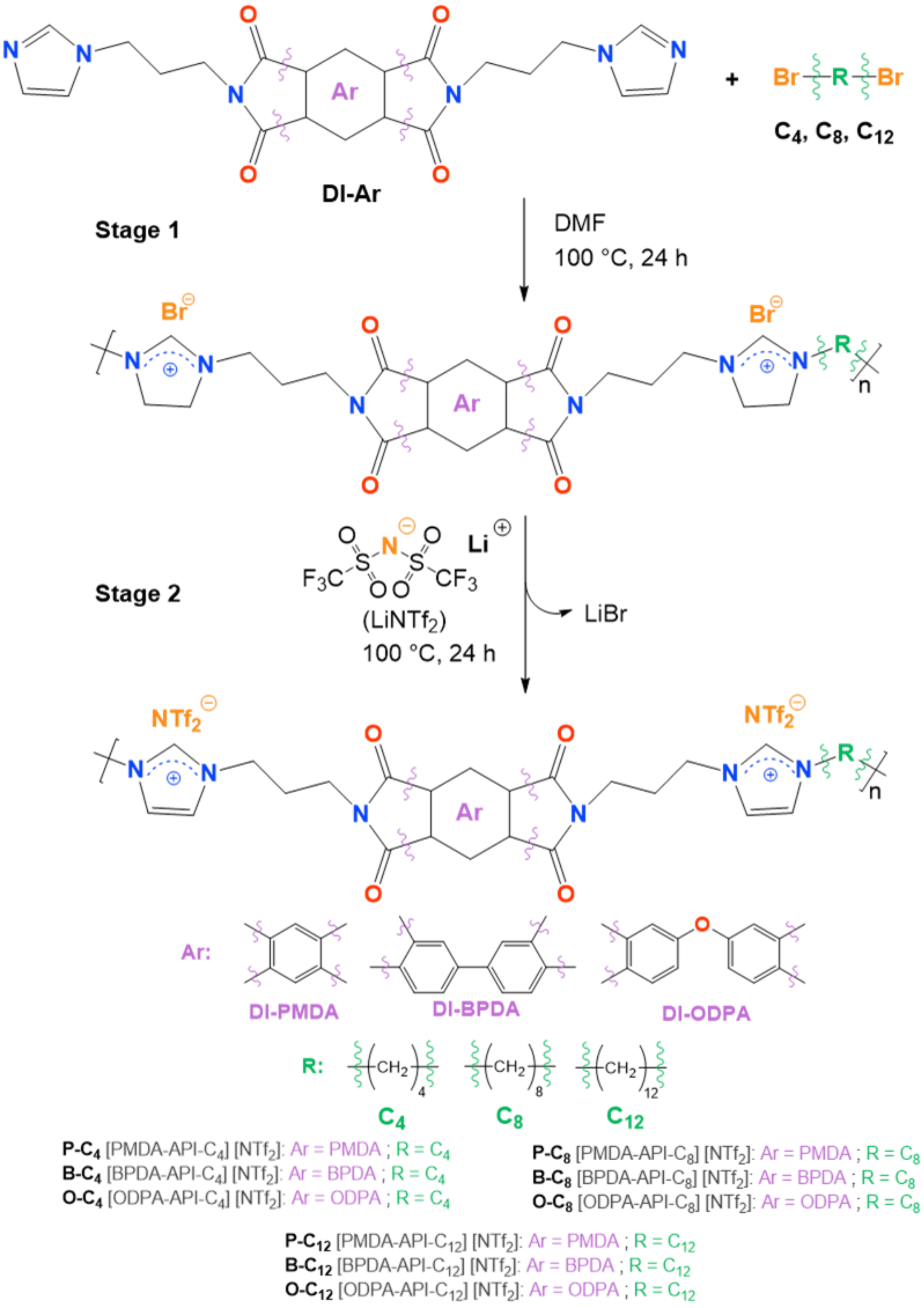

Figure S2: Synthesis scheme of the ionenes.

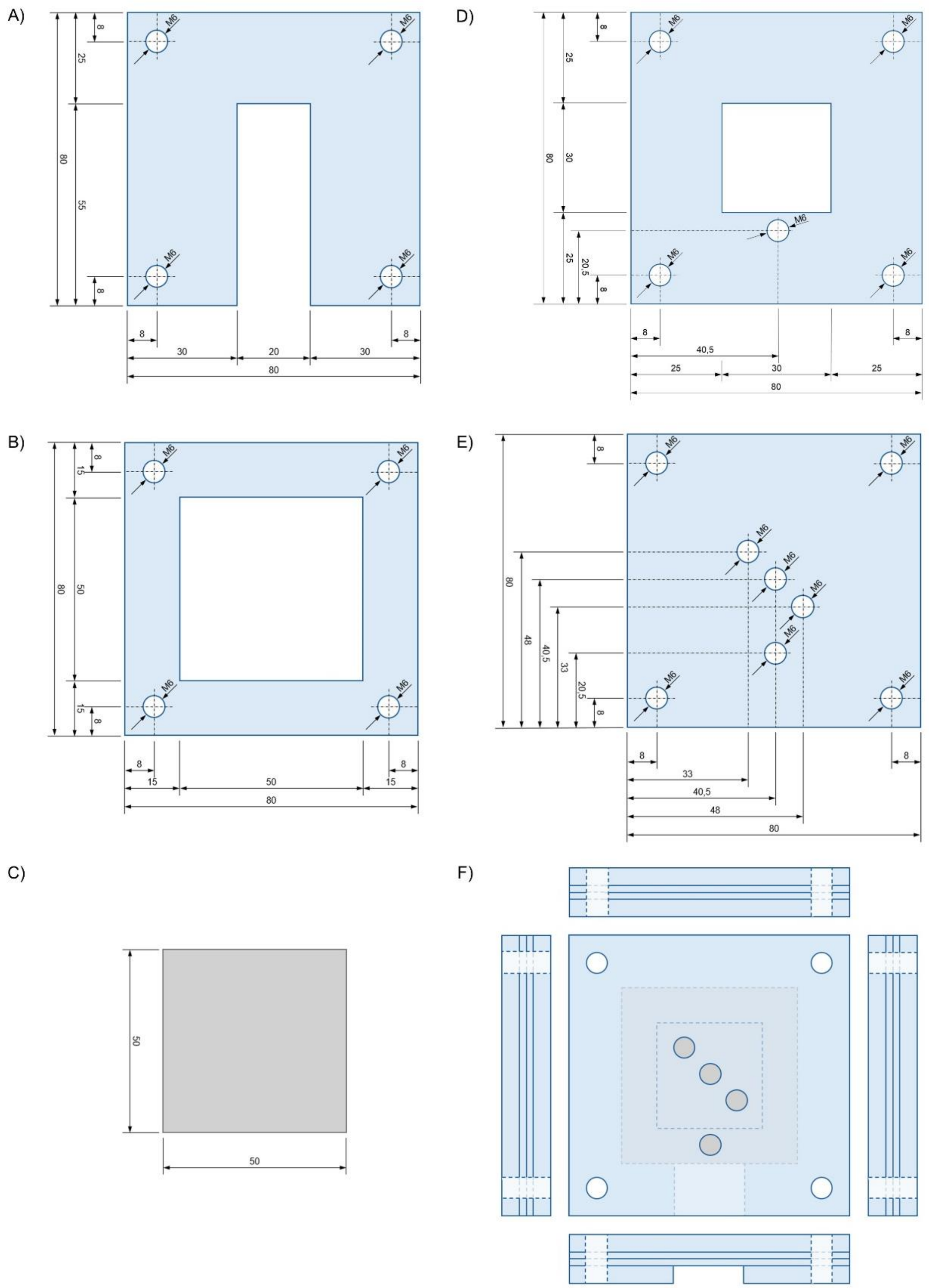

Figure S3: Dimensions of the structural part of the experimental setup.

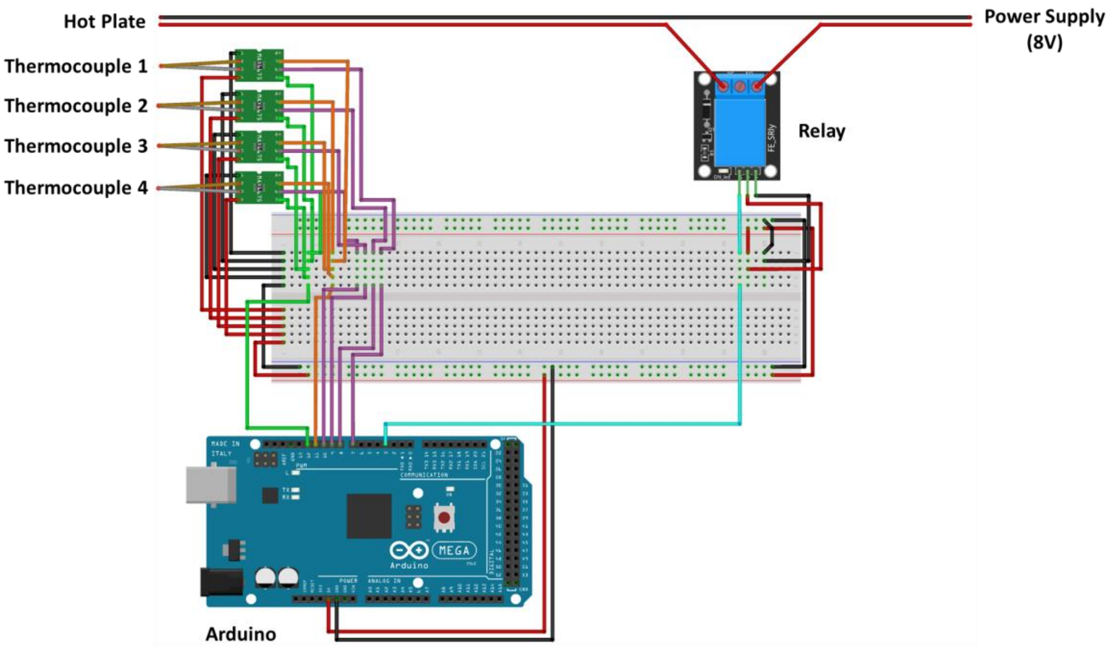

Figure S4: Connection diagram of the electronic section.

Spectroscopic data

P-C$_4$ [PMDA-API-C$_4$] [NTf_2]

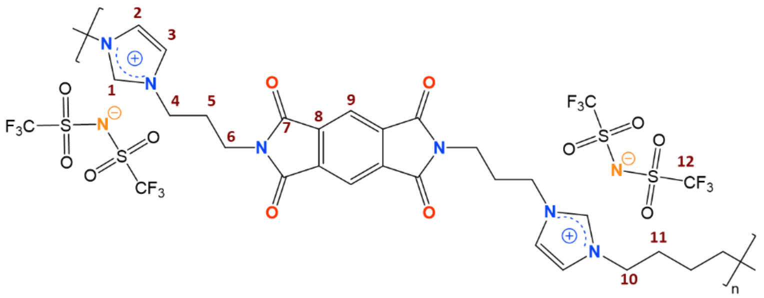

P-C$_4$ spectroscopic data. Yield: 91 %. IR (KBr, cm^{-1}): 3150, 3117, 3092 (C-H, arom.); 2956, 2879 (C-H, aliph); 1771, 1711 (C=O); 1564 (C=C); 1452 (C=N); 1398, 1177 (C-N); 1350, 1132 (SO_2); 1229 (CF_3); 1046 (S-N-S); 730 (subts. imide ring). ^{1}H NMR (DMSO-d_6, δ, ppm): 9.15 (m, 2H, **1**); 8.24 (m, 2H, **9**); 7.81 (t, J = 1.7 Hz, 2H, **3**); 7.81 (t, J = 1.8 Hz, 2H, **2**); 4.26 (m, 8H, **4,10**); 3.68 (m, 4H, **6**); 2.21 (q, J = 6.7 Hz, 4H, **5**); 1.82 (m, 4H, **11**). ^{13}C NMR (DMSO-d_6, δ, ppm): 166.42 (**7**); 137.09 (**8**); 136.23 (**1**); 122.55 (**3**); 122.46 (**2**); 120.75, 118.18 (**12**); 117.19 (**9**); 48.16 (**10**); 46.63 (**4**), 34.92 (**6**), 28.47 (**5**), 26.07 (**11**). ^{19}F NMR (DMSO-d_6, δ, ppm): -78.72 (**12**).

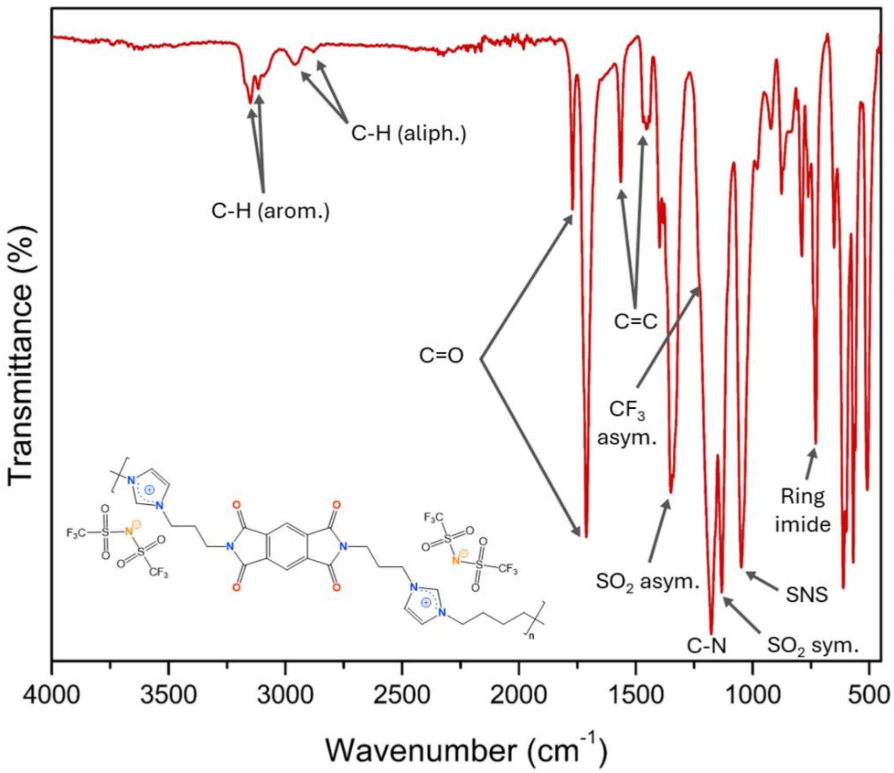
Transmittance (%)
Wavenumber (cm-1)
C-H (arom.)
C-H (aliph.)
C=O
C=C
CF3 asym.
SO2 asym.
C-N
SO2 sym.
SNS
Ring imide
4000
3500
3000
2500
2000
1500
1000
500

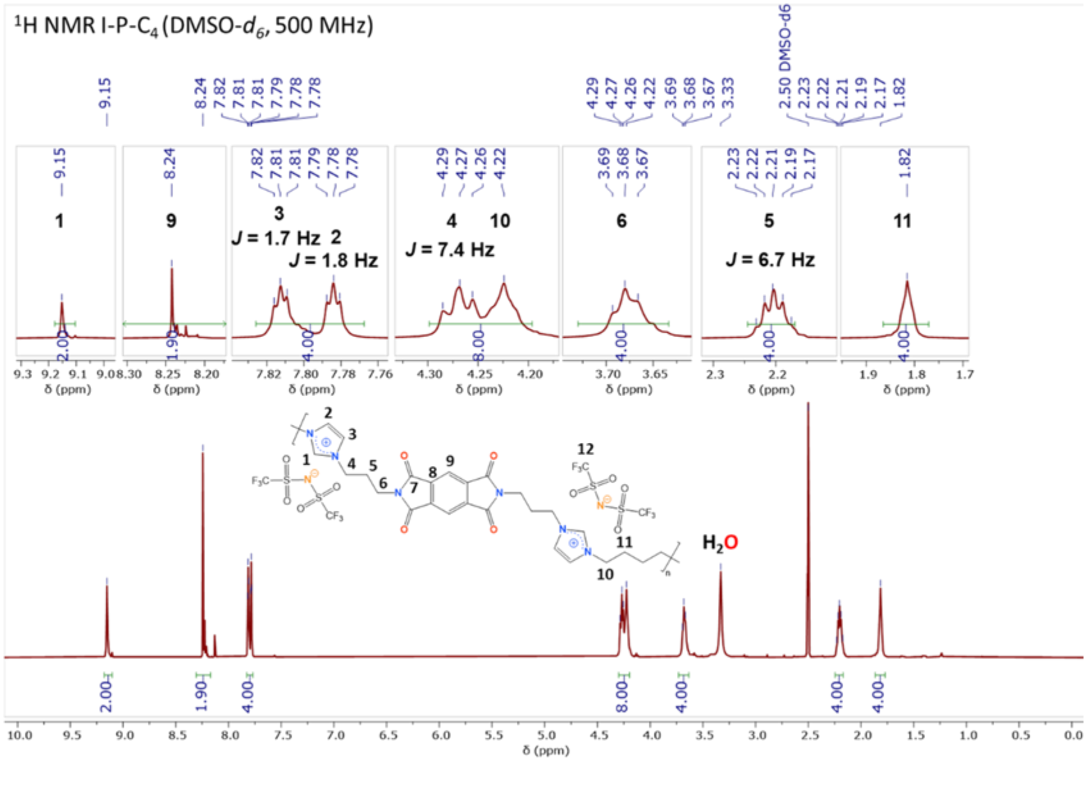
1H NMR I-P-C4 (DMSO-d6, 500 MHz)
DMSO-d6
H2O
1
9
3
J = 1.7 Hz
2
J = 1.8 Hz
4
10
J = 7.4 Hz
6
5
J = 6.7 Hz
11
12
δ (ppm)

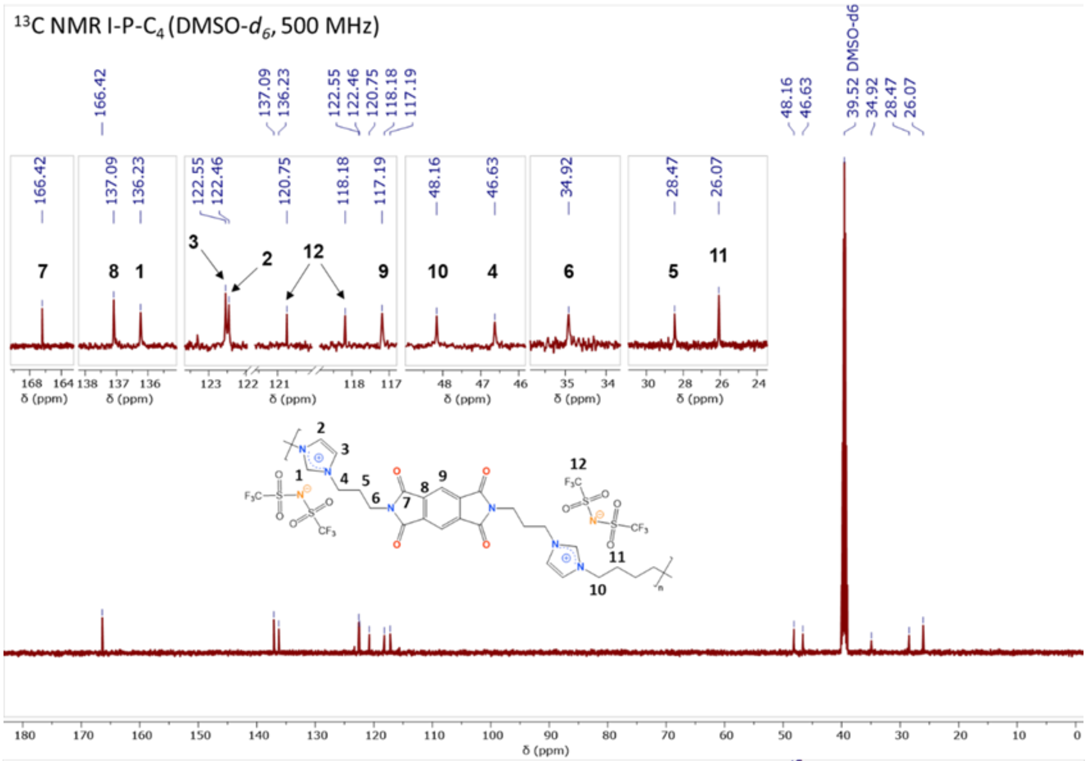

13C NMR I-P-C4 (DMSO-d6, 500 MHz)
166.42
137.09
136.23
122.55
122.46
120.75
118.18
117.19
48.16
46.63
39.52 DMSO-d6
34.92
28.47
26.07
δ (ppm)

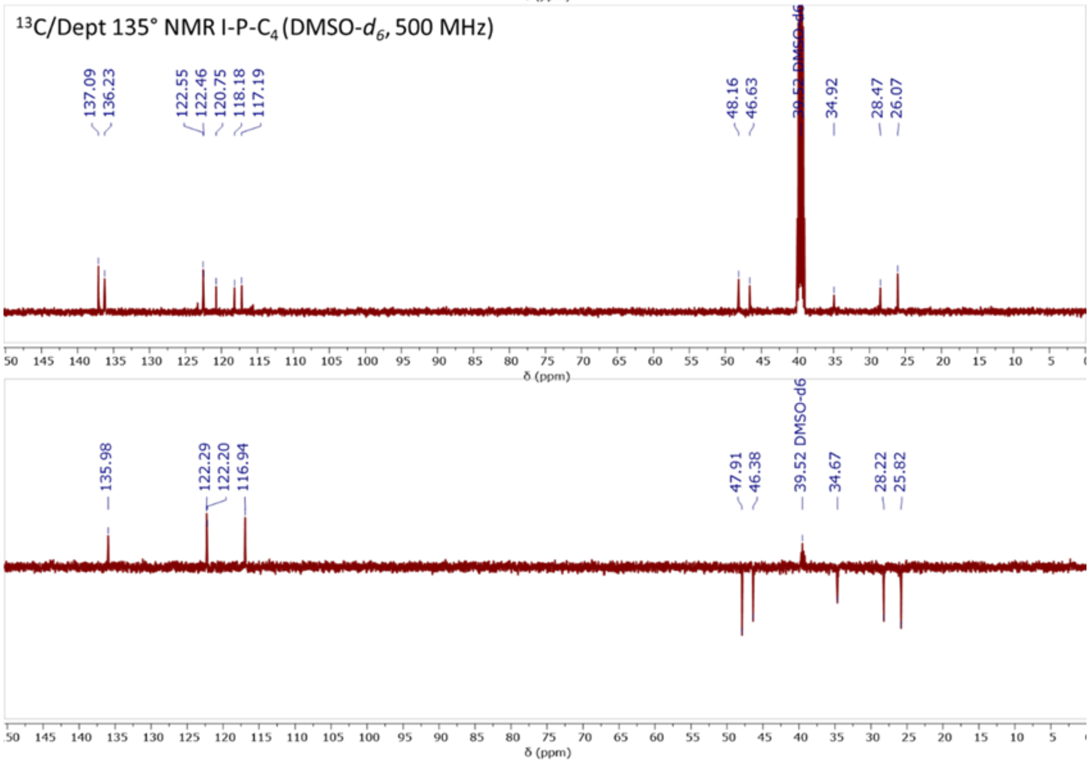

13C/Dept 135° NMR I-P-C4 (DMSO-d6, 500 MHz)
137.09
136.23
122.55
122.46
120.75
118.18
117.19
48.16
46.63
34.92
28.47
26.07
135.98
122.29
122.20
116.94
47.91
46.38
39.52 DMSO-d6
34.67
28.22
25.82
δ (ppm)

COSY NMR I-P-C_4 (DMSO-d_6, 500 MHz)

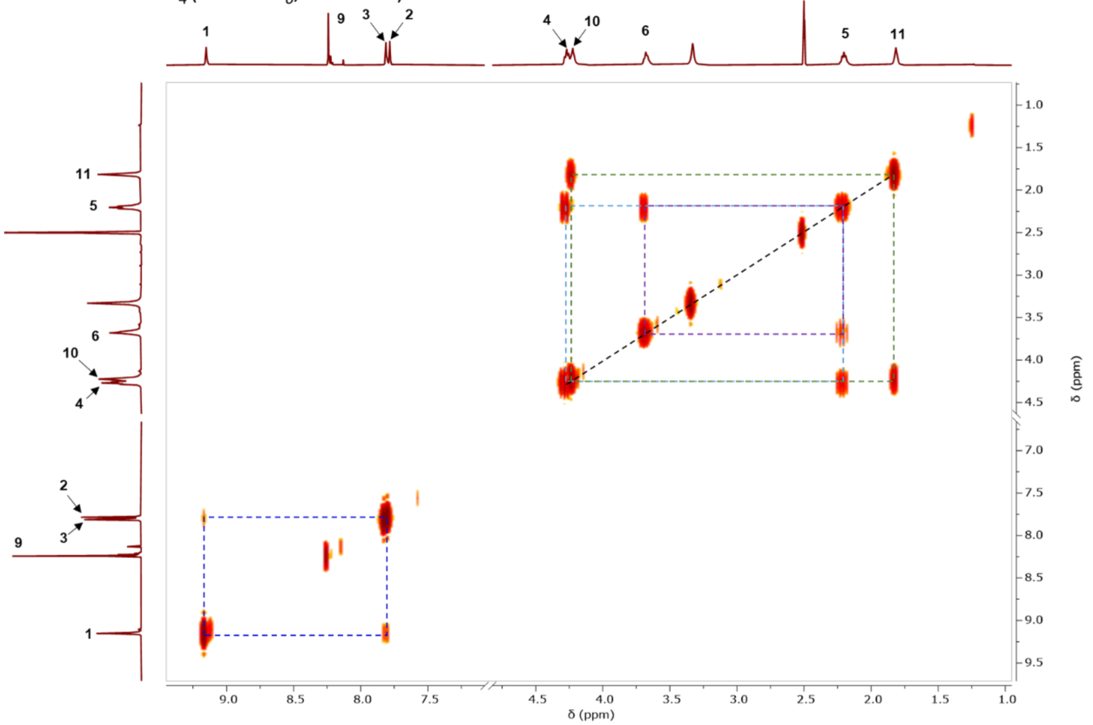

HSQC NMR I-P-C_4 (DMSO-d_6, 500 MHz)

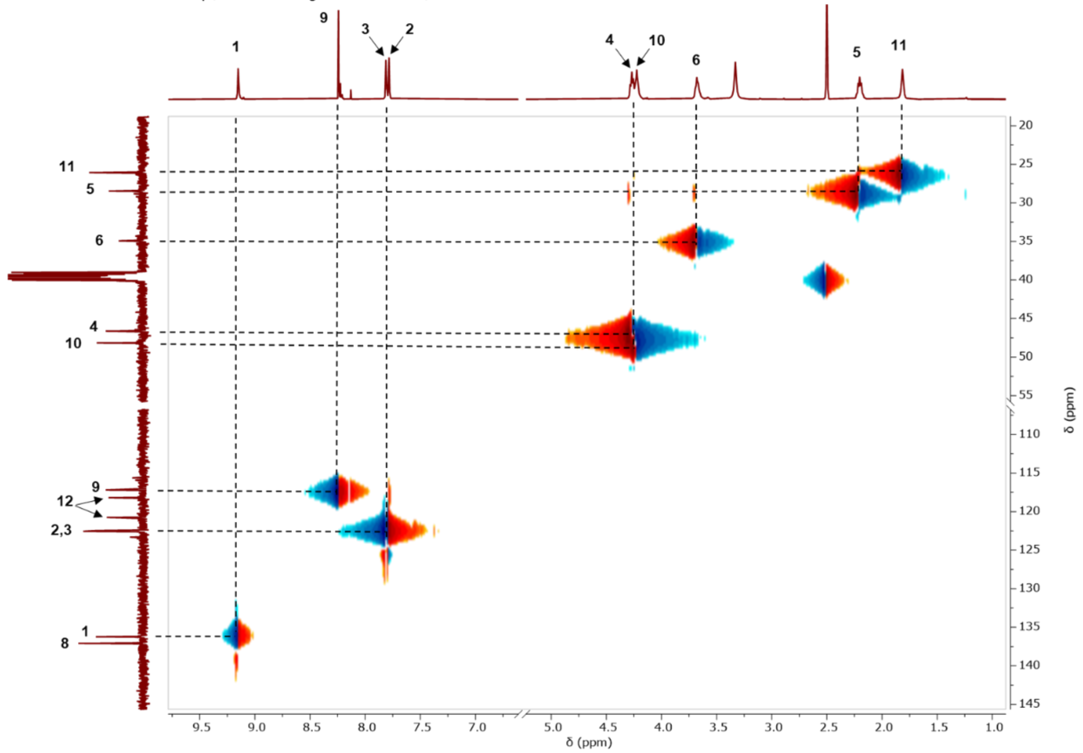

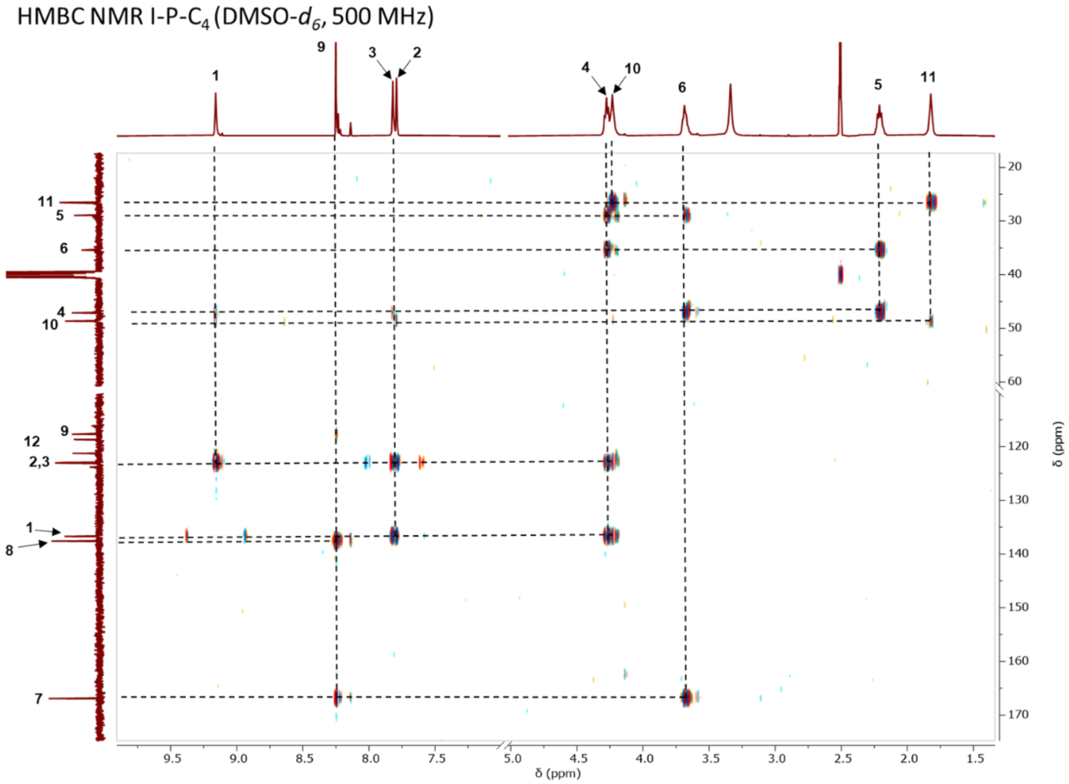

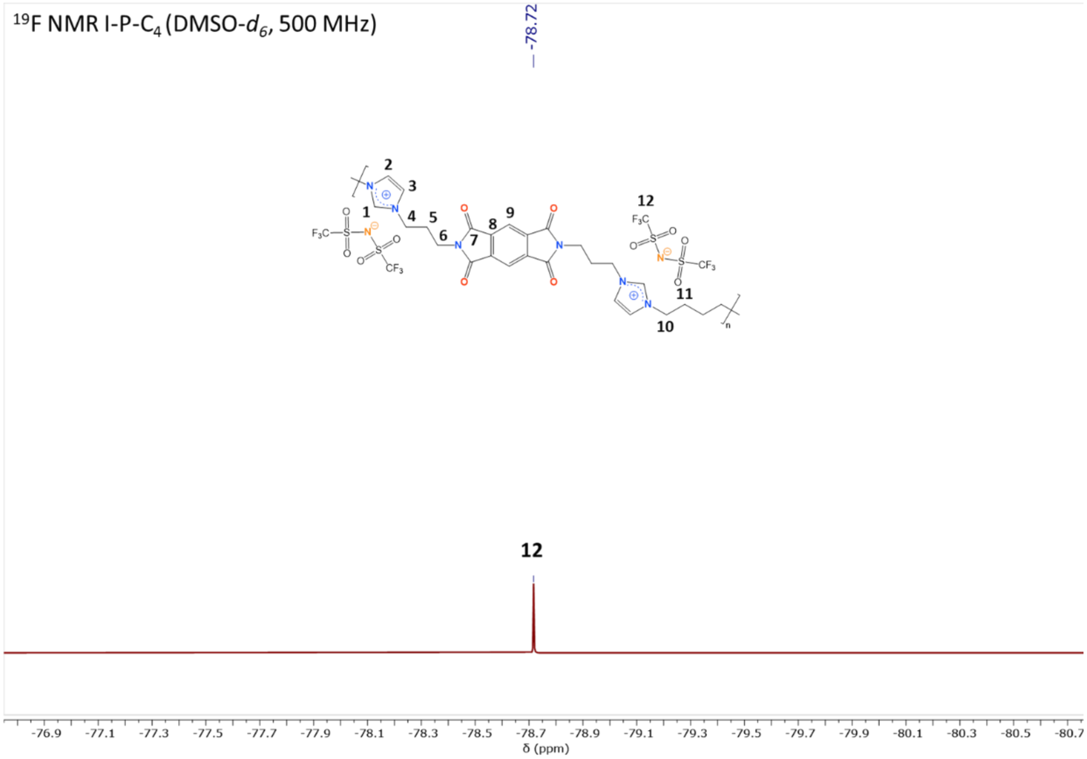

Figure S5: FT-IR-ATR and ^{1}H, ^{13}C, DEPT 135°, COSY, HSQC, HMBC and ^{19}F NMR spectra of ionene P-C_4

P-C$_8$ [PMDA-API-C$_8$] [NTf_2]

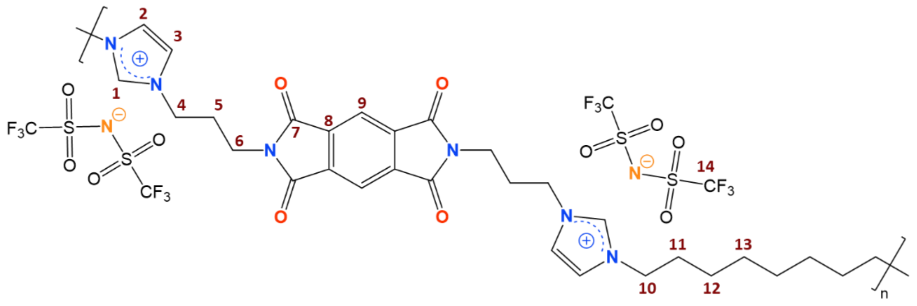

P-C$_8$ spectroscopic data. Yield: 94 %. IR (KBr, cm^{-1}): 3152, 3116, 3096 (C-H, arom.); 2938, 2862 (C-H, aliph); 1772, 1712 (C=O); 1564 (C=C); 1459 (C=N); 1396, 1177 (C-N); 1346, 1132 (SO_2); 1226 (CF_3); 1051 (S-N-S); 728 (subts. imide ring). ^{1}H NMR (DMSO-d_6, δ, ppm): 9.16 (m, 2H, **1**); 8.23 (m, 2H, **9**); 7.79 (m, 4H, **2**,**3**); 4.26 (t, J = 7.3, 4H, **4**); 4.15 (t, J = 7.2, 4H, **10**); 3.66 (m, 4H, **6**); 2.20 (q, J = 6.7 Hz, 4H, **5**); 1.79 (q, J = 7.1 Hz, 4H, **11**); 1.29 (m, 8H, **12,13**). ^{13}C NMR (DMSO-d_6, δ, ppm): 166.40 (**7**); 137.08 (**8**); 136.09 (**1**); 122.44 (**2,3**); 120.75, 118.19 (**14**); 117.15 (**9**); 48.89 (**10**); 46.60 (**4**), 34.93 (**6**), 29.35 (**11**); 28.45 (**5**), 28.27 (**12**); 25.54 (**13**). ^{19}F NMR (DMSO-d_6, δ, ppm): -78.77 (**14**).

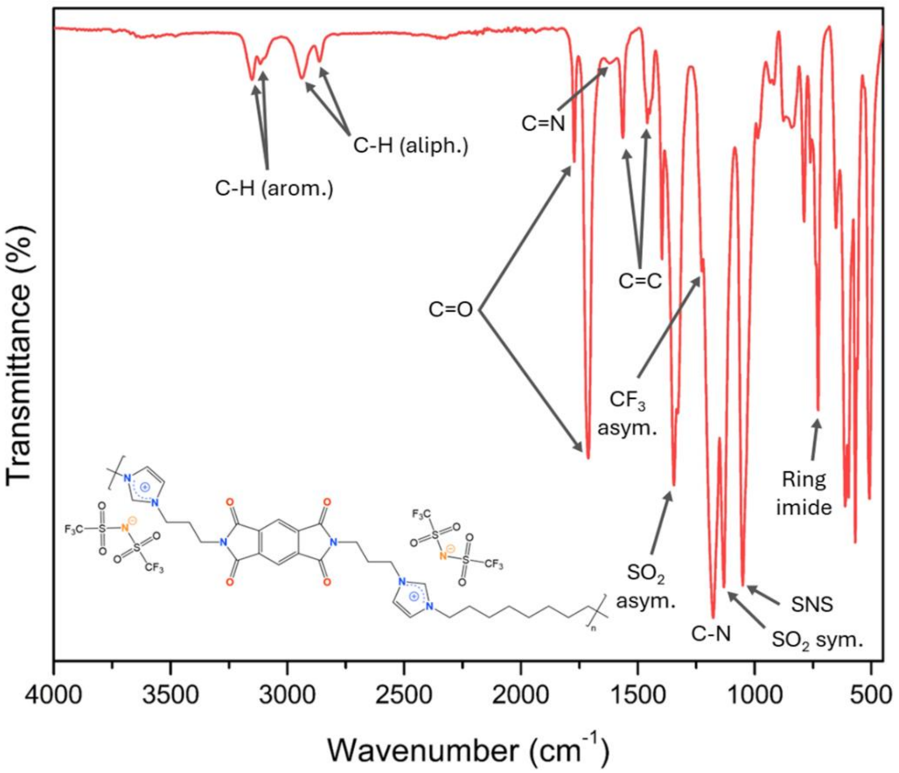

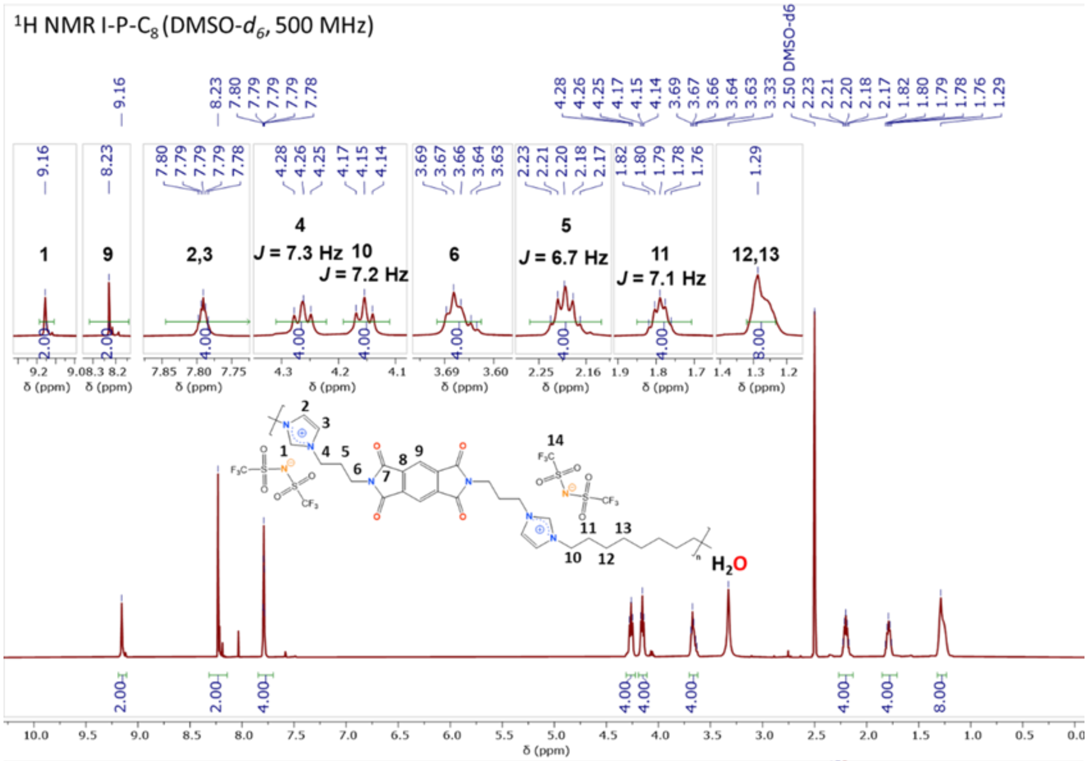

¹H NMR I-P-C8 (DMSO-d6, 500 MHz)
4
J = 7.3 Hz
10
J = 7.2 Hz
5
J = 6.7 Hz
11
J = 7.1 Hz
12,13
2,3
6
H2O
δ (ppm)

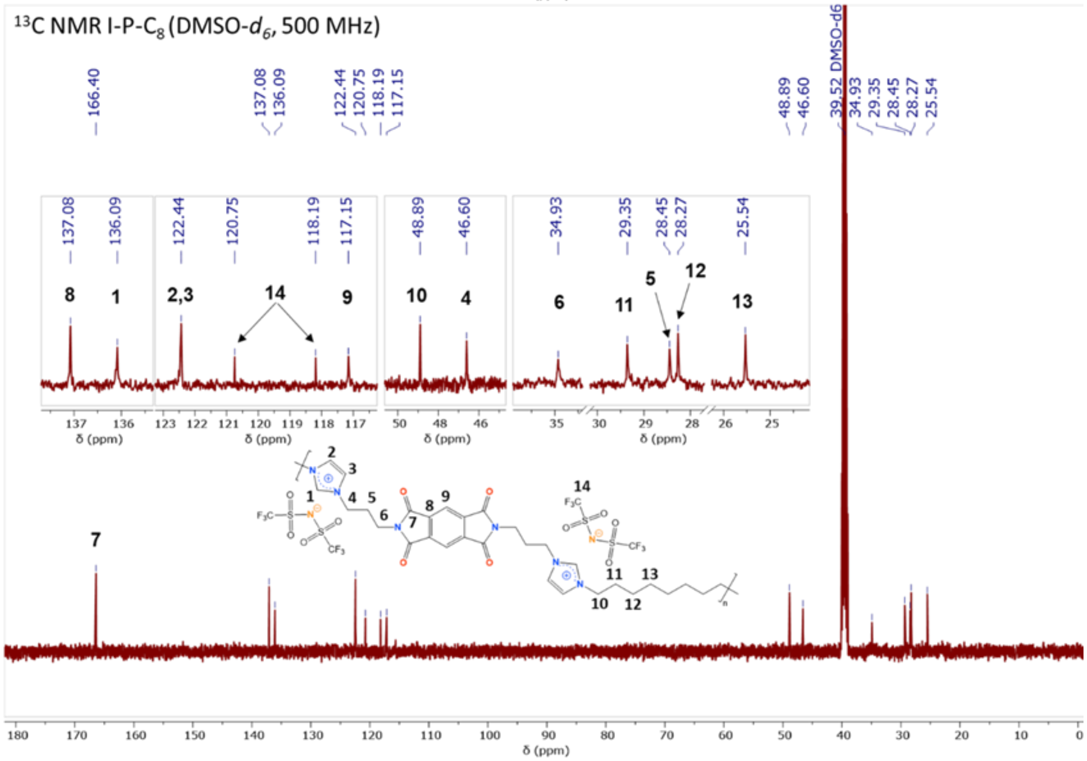

¹³C NMR I-P-C8 (DMSO-d6, 500 MHz)
8
1
2,3
14
9
10
4
6
11
5
12
13
7
δ (ppm)

^{13}C/Dept 45° NMR I-P-C$_8$ (DMSO-d_6, 500 MHz)

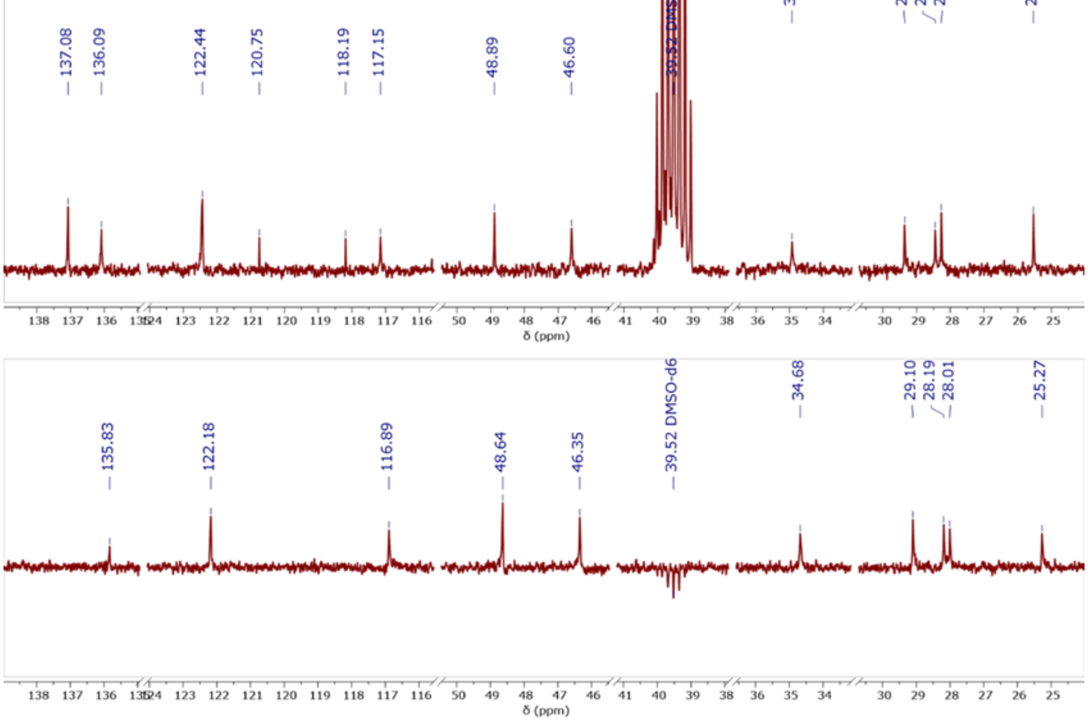

COSY NMR I-P-C$_8$ (DMSO-d_6, 500 MHz)

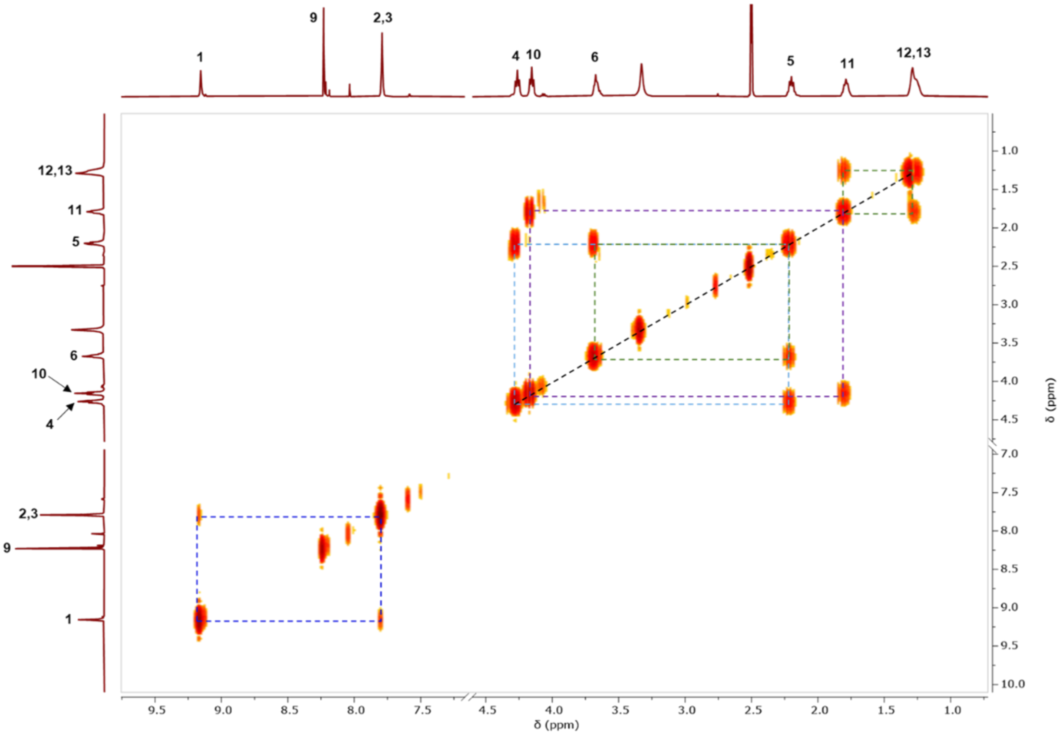

HSQC NMR I-P-C_8 (DMSO-d_6, 500 MHz)

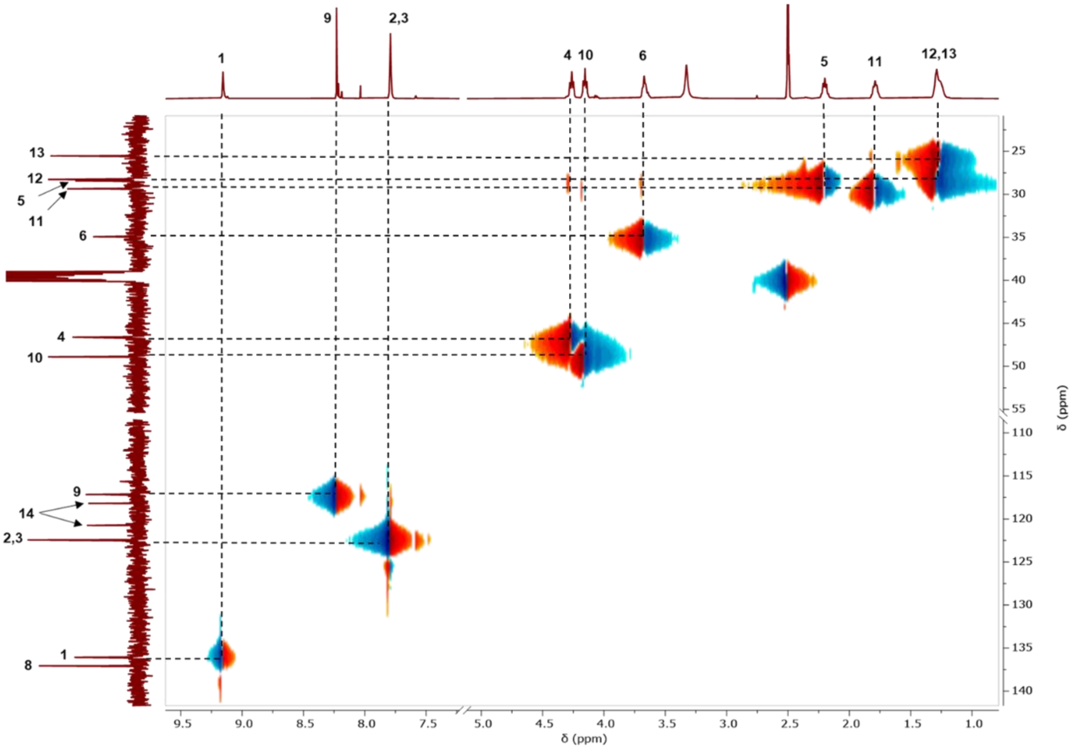

HMBC NMR I-P-C_8 (DMSO-d_6, 500 MHz)

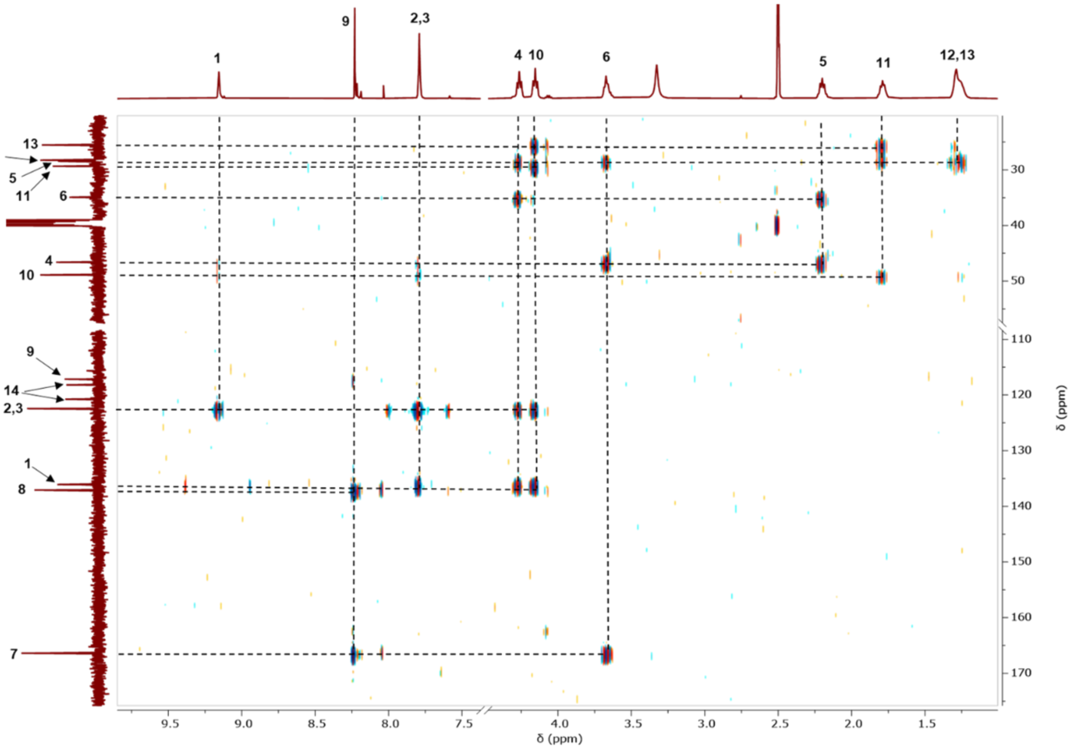

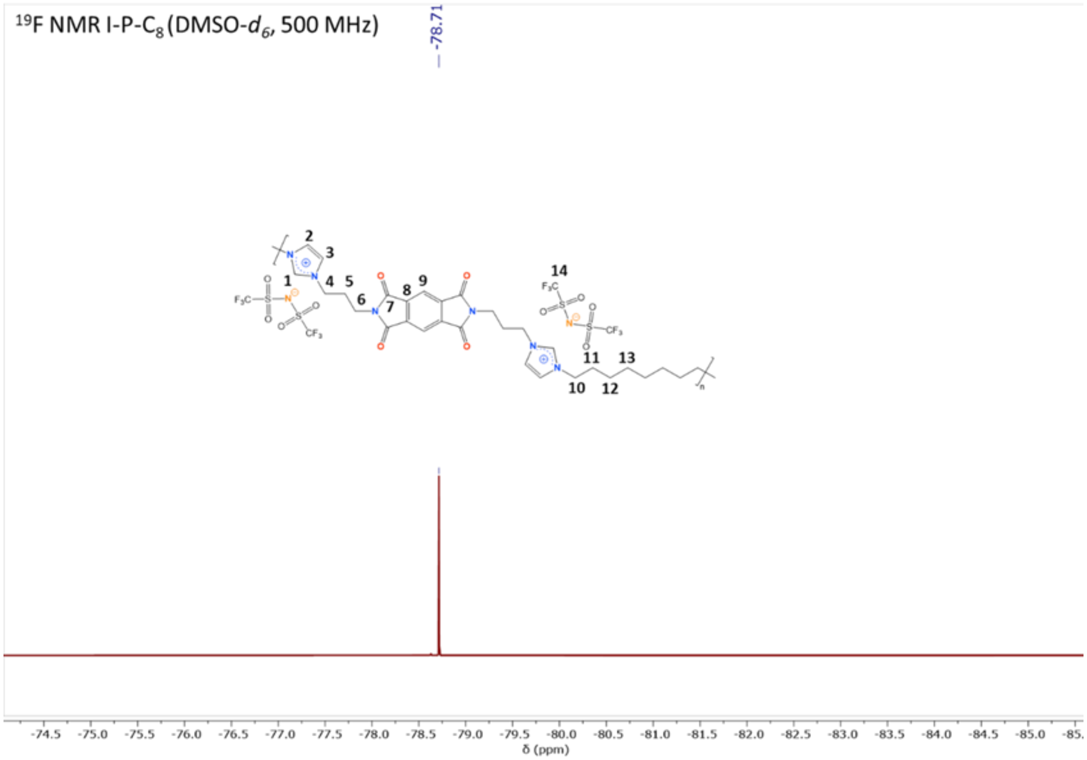

Figure S6: FT-IR-ATR and ^{1}H, ^{13}C, DEPT 135°, COSY, HSQC, HMBC and ^{19}F NMR spectra of ionene P-C8.

P-C12 [PMDA-API-C12] [NTf2]

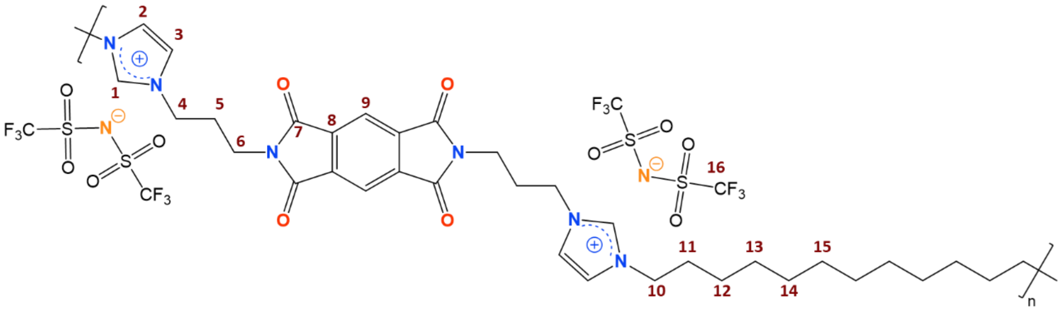

P-C12 spectroscopic data. Yield: 97 %. IR (KBr, cm^{-1}): 3151, 3115, 3095 (C-H, arom.); 2929, 2857 (C-H, aliph); 1772, 1715 (C=O); 1564 (C=C); 1460 (C=N); 1395, 1179 (C-N); 1346, 1133 (SO_2); 1226 (CF_3); 1052 (S-N-S); 728 (subts. imide ring). ^{1}H NMR (DMSO-*d*6, δ, ppm): 9.15 (m, 2H, **1**); 8.23 (m, 2H, **9**); 7.79 (m, 4H, **2**,**3**); 4.26 (t, *J* = 7.4, 4H, **4**); 4.15 (t, *J* = 7.3, 4H, **10**); 3.66 (m, 4H, **6**); 2.20 (q, *J* = 7.0Hz, 4H, **5**); 1.78 (q, *J* = 7.7 Hz, 4H, **11**); 1.25 (m, 16H, **12-15**). ^{13}C NMR (DMSO-*d*6, δ, ppm): 166.39 (**7**); 137.07 (**8**); 136.09 (**1**); 122.46 (**2**); 122.42 (**3**); 120.75, 118.19 (**16**); 117.14 (**9**); 48.91 (**10**); 46.60 (**4**), 34.92 (**6**), 29.38 (**11**); 29.02 (**12**); 28.92 (**5**), 28.46 (**13**); 28.26 (**14**); 25.58 (**15**). ^{19}F NMR (DMSO-*d*6, δ, ppm): -78.72 (**16**).

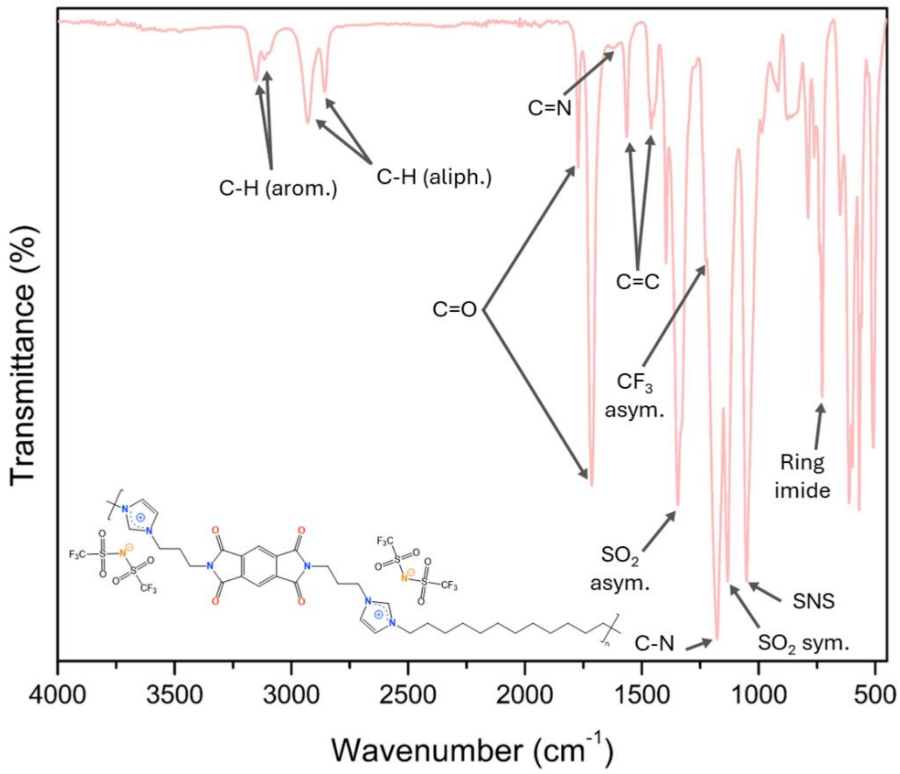

Transmittance (%)
Wavenumber (cm^{-1})
4000
3500
3000
2500
2000
1500
1000
500
C-H (arom.)
C-H (aliph.)
C=N
C=O
C=C
CF_3 asym.
Ring imide
SO_2 asym.
SNS
C-N
SO_2 sym.

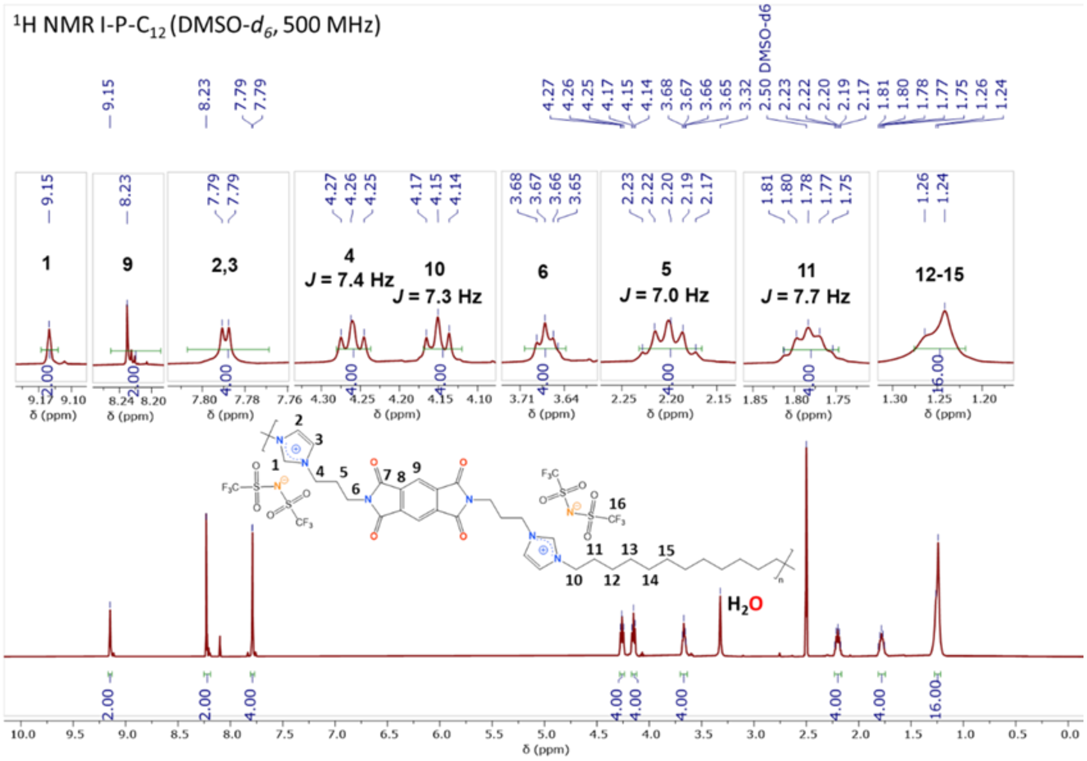

^{1}H NMR I-P-C_{12} (DMSO-d_6, 500 MHz)
DMSO-d6
H$_2$O
1
9
2,3
4 J = 7.4 Hz
10 J = 7.3 Hz
6
5 J = 7.0 Hz
11 J = 7.7 Hz
12-15
δ (ppm)

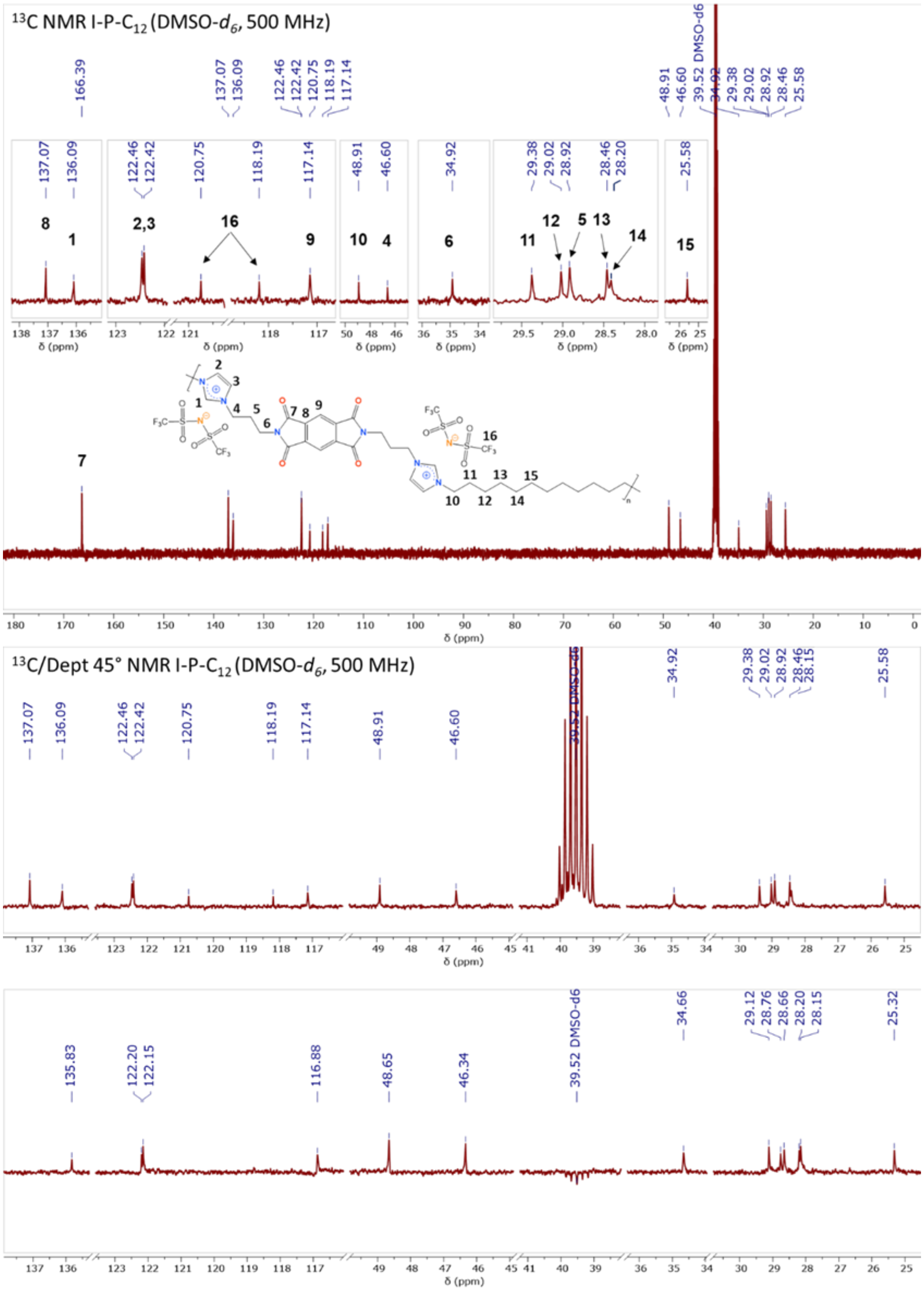

^{13}C NMR I-P-C$_{12}$ (DMSO-d_6, 500 MHz)
^{13}C/Dept 45° NMR I-P-C$_{12}$ (DMSO-d_6, 500 MHz)
δ (ppm)

COSY NMR I-P-C_{12} (DMSO-d_6, 500 MHz)

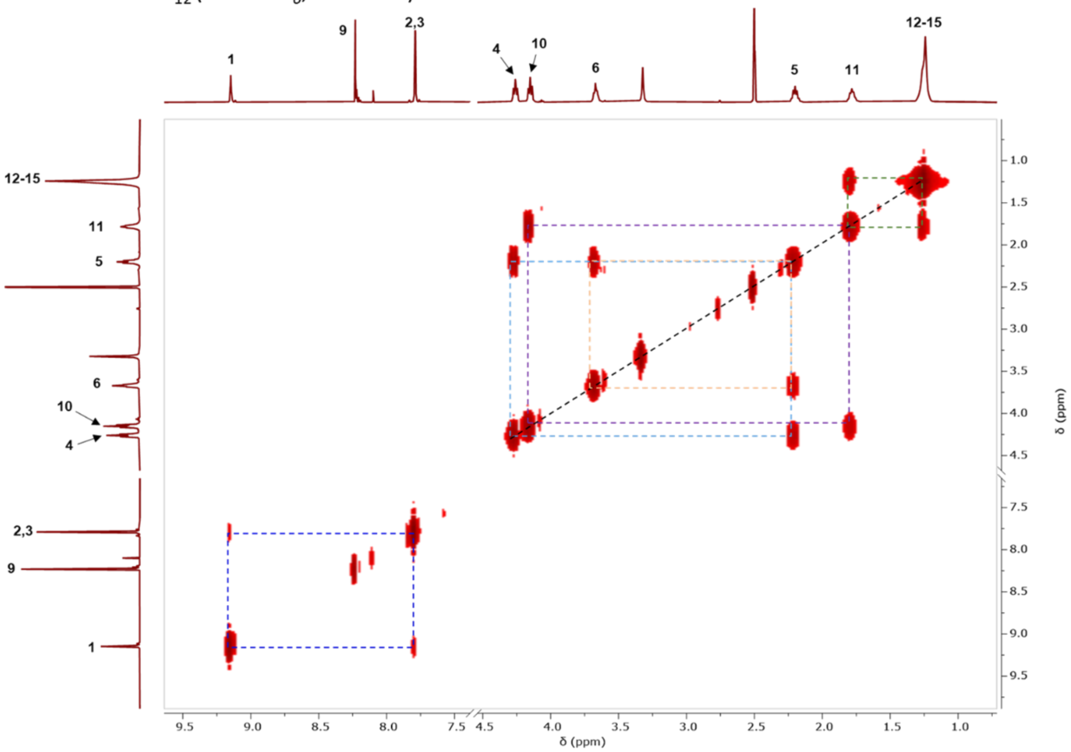

HSQC NMR I-P-C_{12} (DMSO-d_6, 500 MHz)

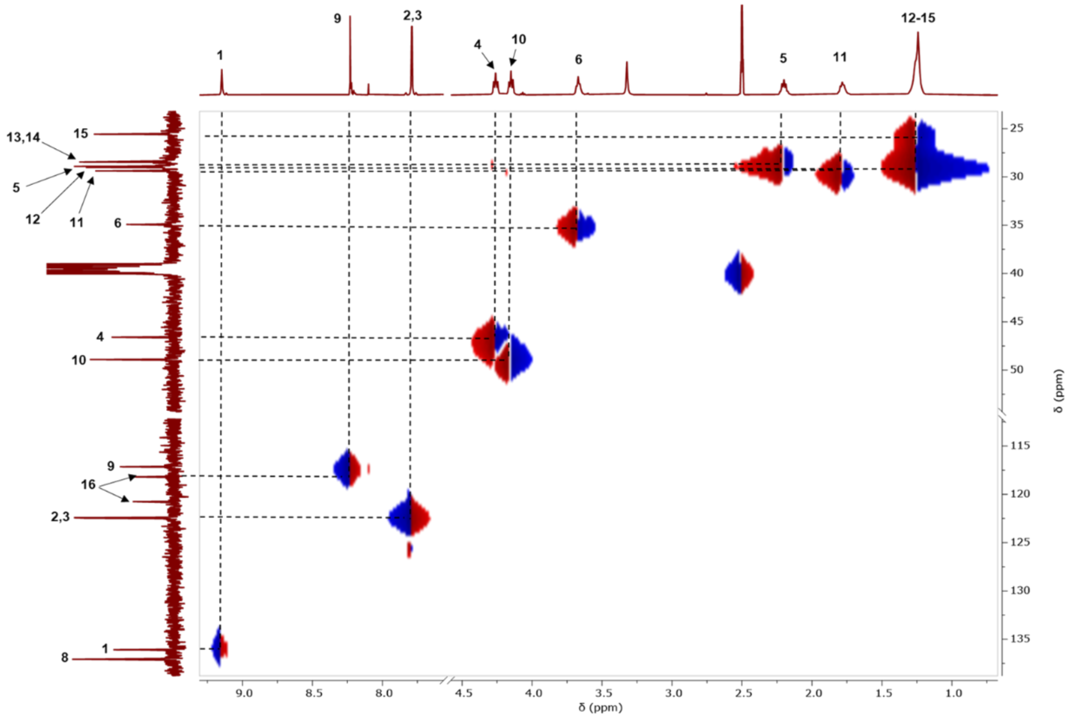

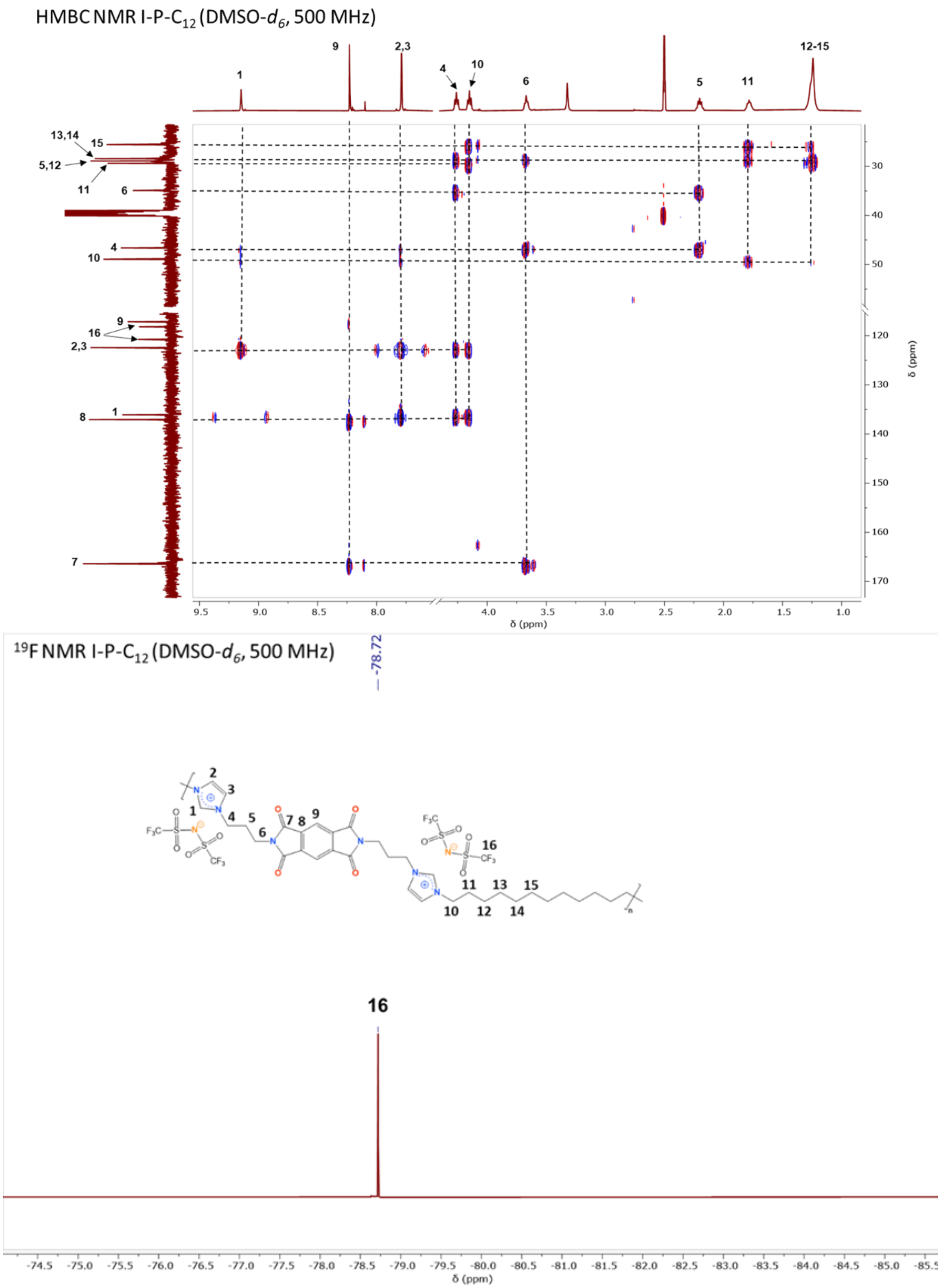

Figure S7: FT-IR-ATR and ^{1}H, ^{13}C, DEPT 45°, COSY, HSQC, HMBC and ^{19}F NMR spectra of ionene P-C_{12}

B-C_4 [BPDA-API-C_4] [NTf_2]

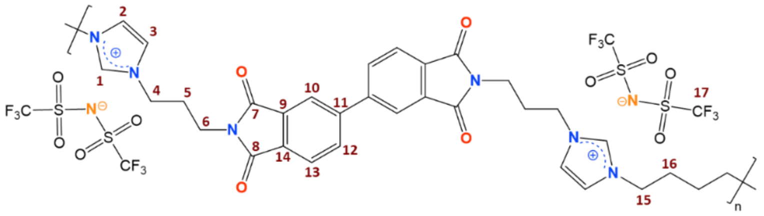

B-C_4 spectroscopic data. Yield: 94 %. IR (KBr, cm^{-1}): 3152, 3117, 3093 (C-H, arom.); 2952, 2851 (C-H, aliph); 1770, 1705 (C=O); 1622, 1565 (C=C); 1444 (C=N); 1395, 1175 (C-N); 1346, 1131 (SO_2); 1226 (CF_3); 1050 (S-N-S); 739 (subts. imide ring). ^{1}H NMR (DMSO-d_6, δ, ppm): 9.15 (m, 2H, **1**); 8.27 (m, 4H, **10,12**); 8.00 (m, 2H, **13**); 7.81 (m, 4H, **2,3**); 4.25 (m, 8H, **4,15**); 3.65 (m, 4H, **6**); 2.19 (q, J = 6.8 Hz, 4H, **5**); 1.82 (m, 4H, **16**). ^{13}C NMR (DMSO-d_6, δ, ppm): 166.64, 166.60 (**7,8**); 144.19 (**11**); 136.24 (**1**); 133.39 (**12**); 133.91 (**9**); 131.58 (**14**); 123.82 (**13**); 122.57 (**2**); 122.44 (**3**); 121.83 (**10**); 120.75, 118.19 (**19**); 48.16 (**15**); 46.64 (**4**), 34.50 (**6**), 28.65 (**5**), 26.07 (**16**). ^{19}F NMR (DMSO-d_6, δ, ppm): -78.71 (**19**).

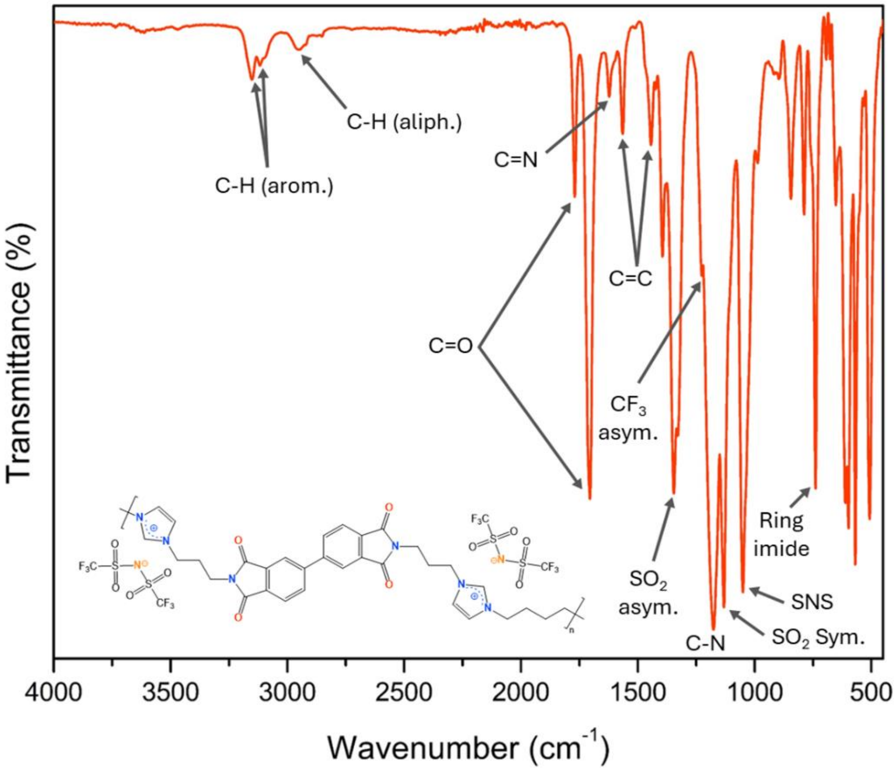

^1H NMR I-B-C_4 (DMSO-d_6, 500 MHz)

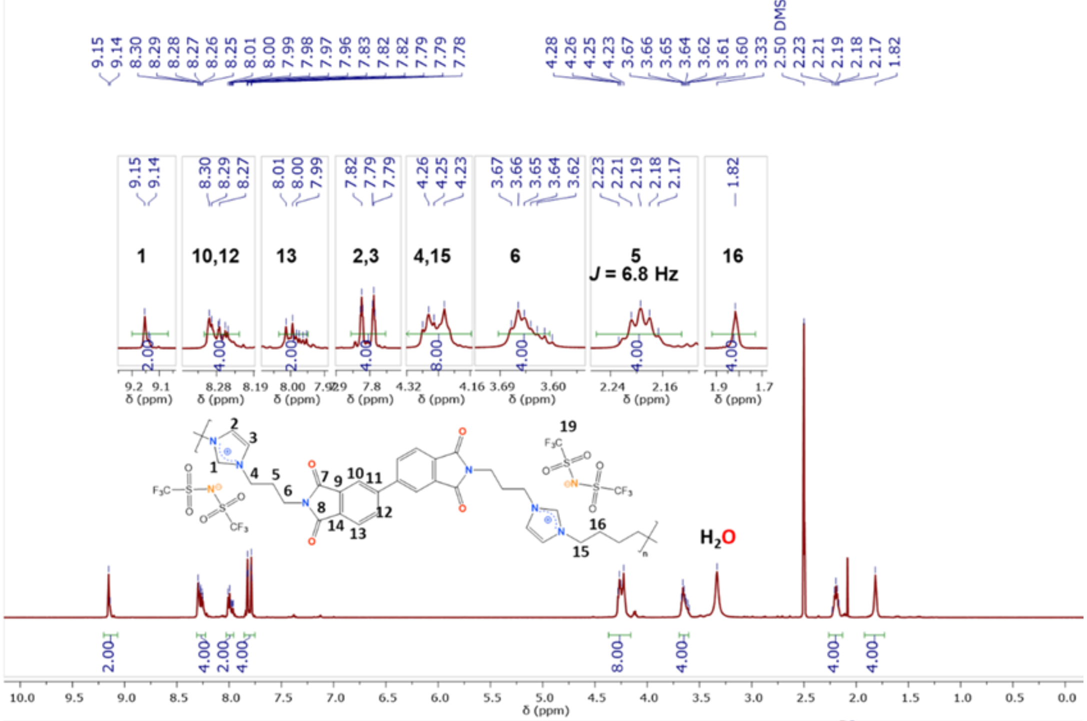

^{13}C NMR I-B-C_4 (DMSO-d_6, 500 MHz)

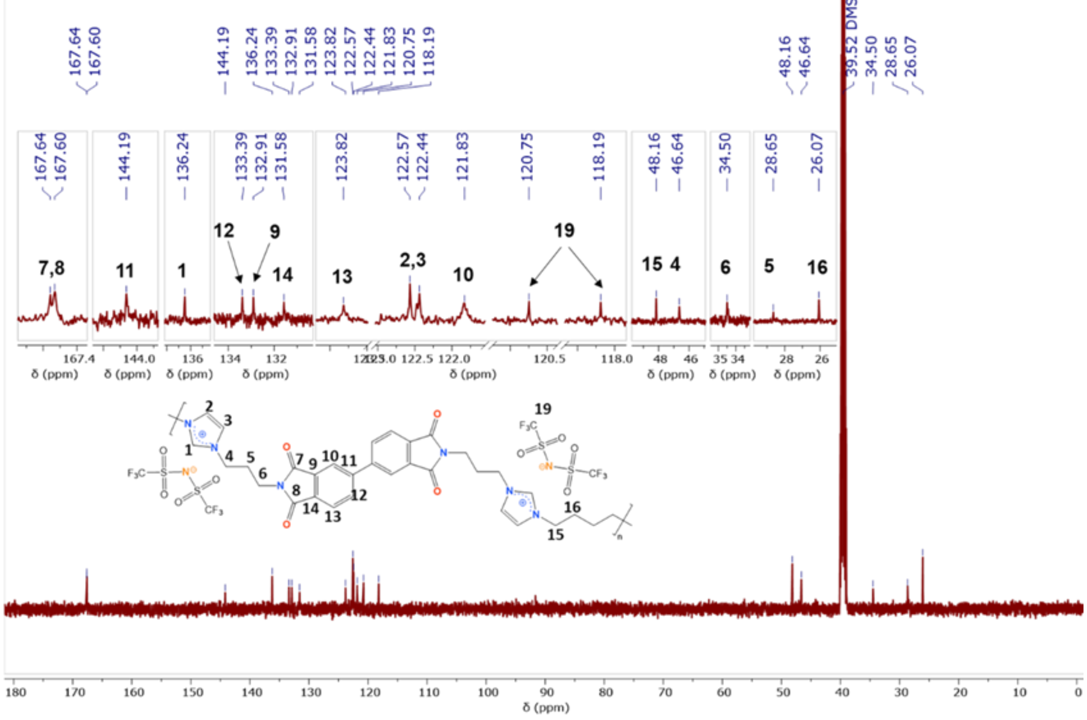

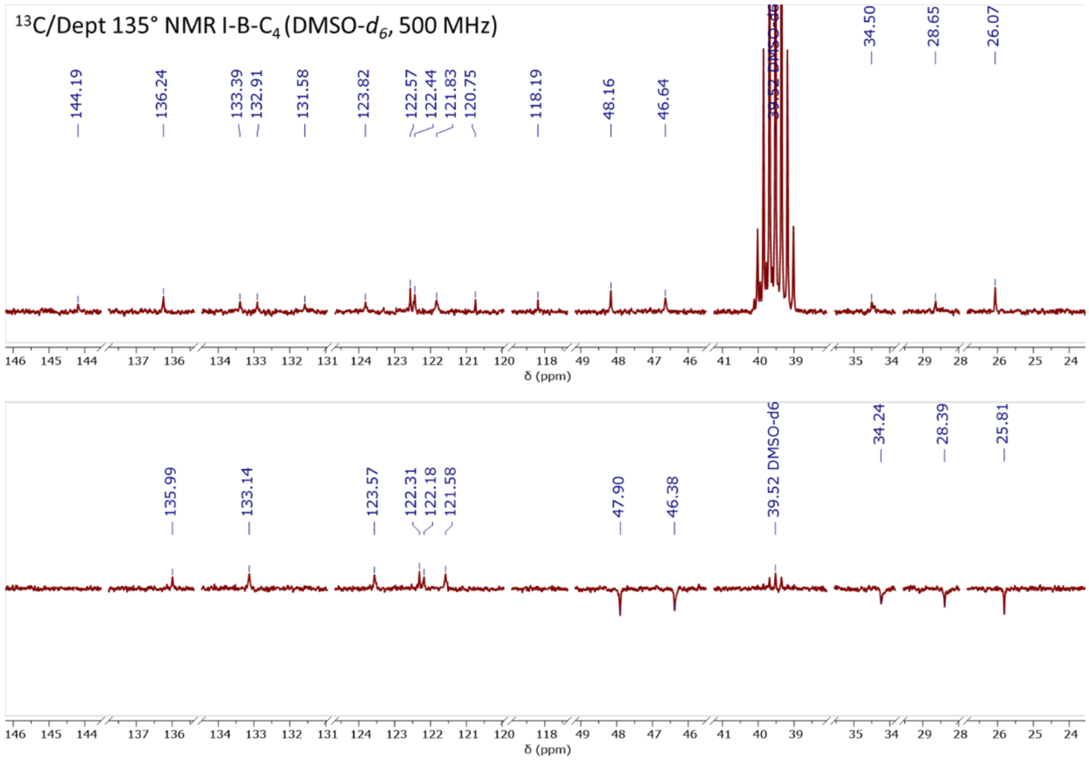

^{13}C/Dept 135° NMR I-B-C$_4$ (DMSO-d_6, 500 MHz)
144.19
136.24
133.39
132.91
131.58
123.82
122.57
122.44
121.83
120.75
118.19
48.16
46.64
39.52 DMSO-d6
34.50
28.65
26.07
135.99
133.14
123.57
122.31
122.18
121.58
47.90
46.38
39.52 DMSO-d6
34.24
28.39
25.81
δ (ppm)

COSY NMR I-B-C_4 (DMSO-d_6, 500 MHz)

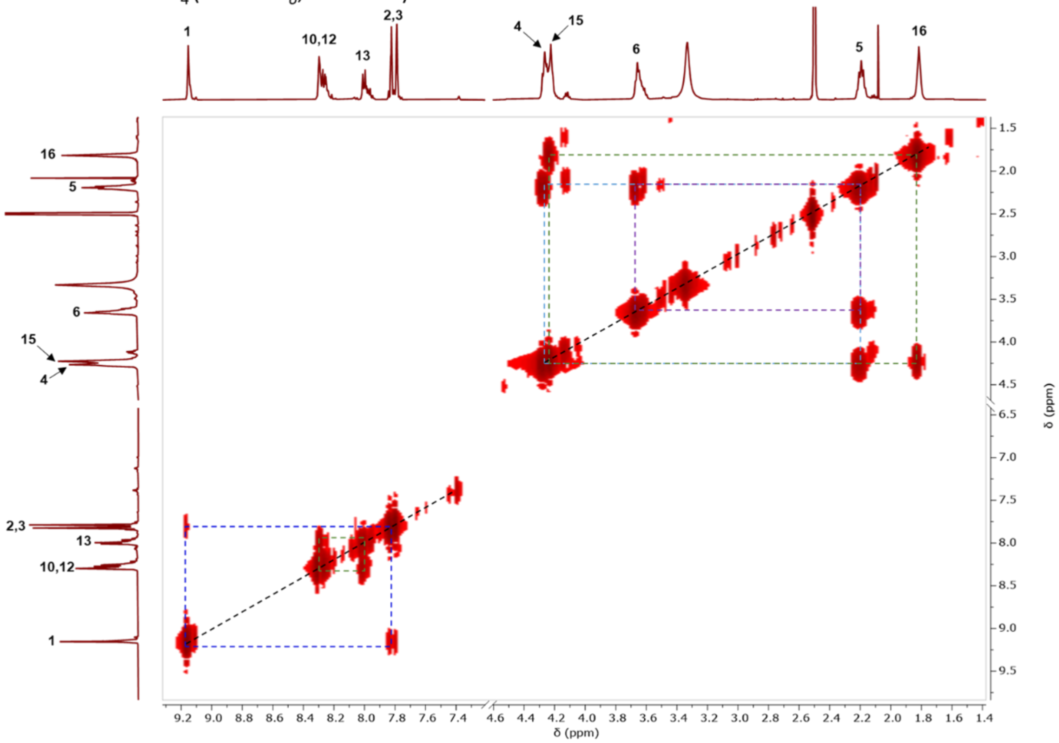

HSQC NMR I-B-C_4 (DMSO-d_6, 500 MHz)

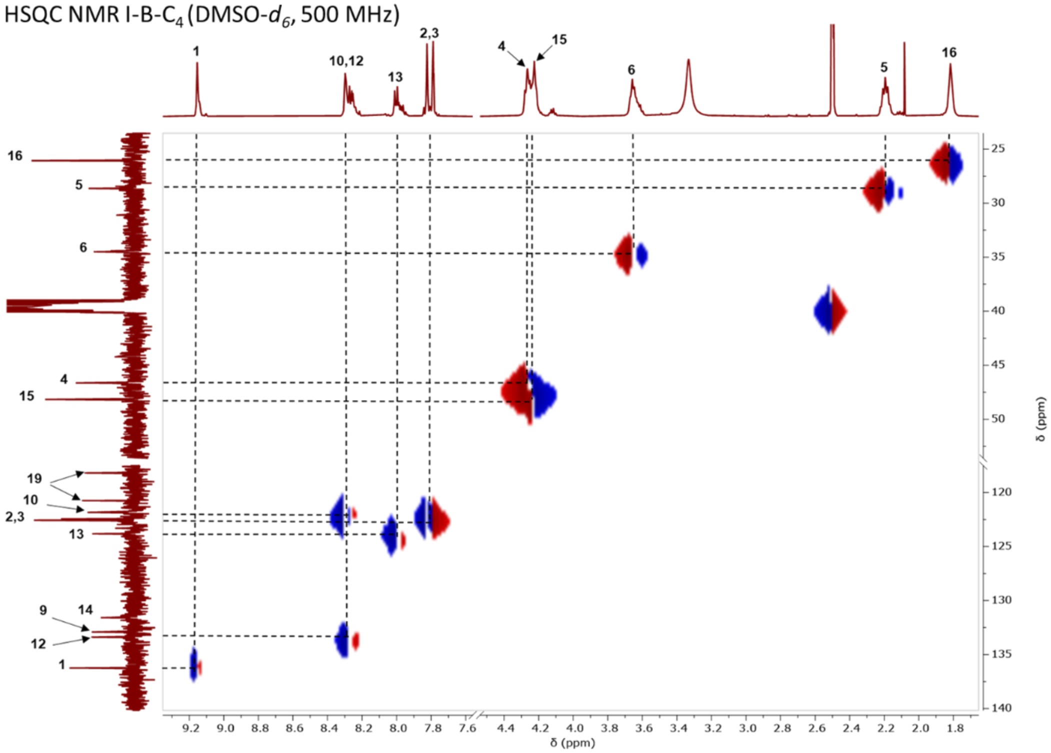

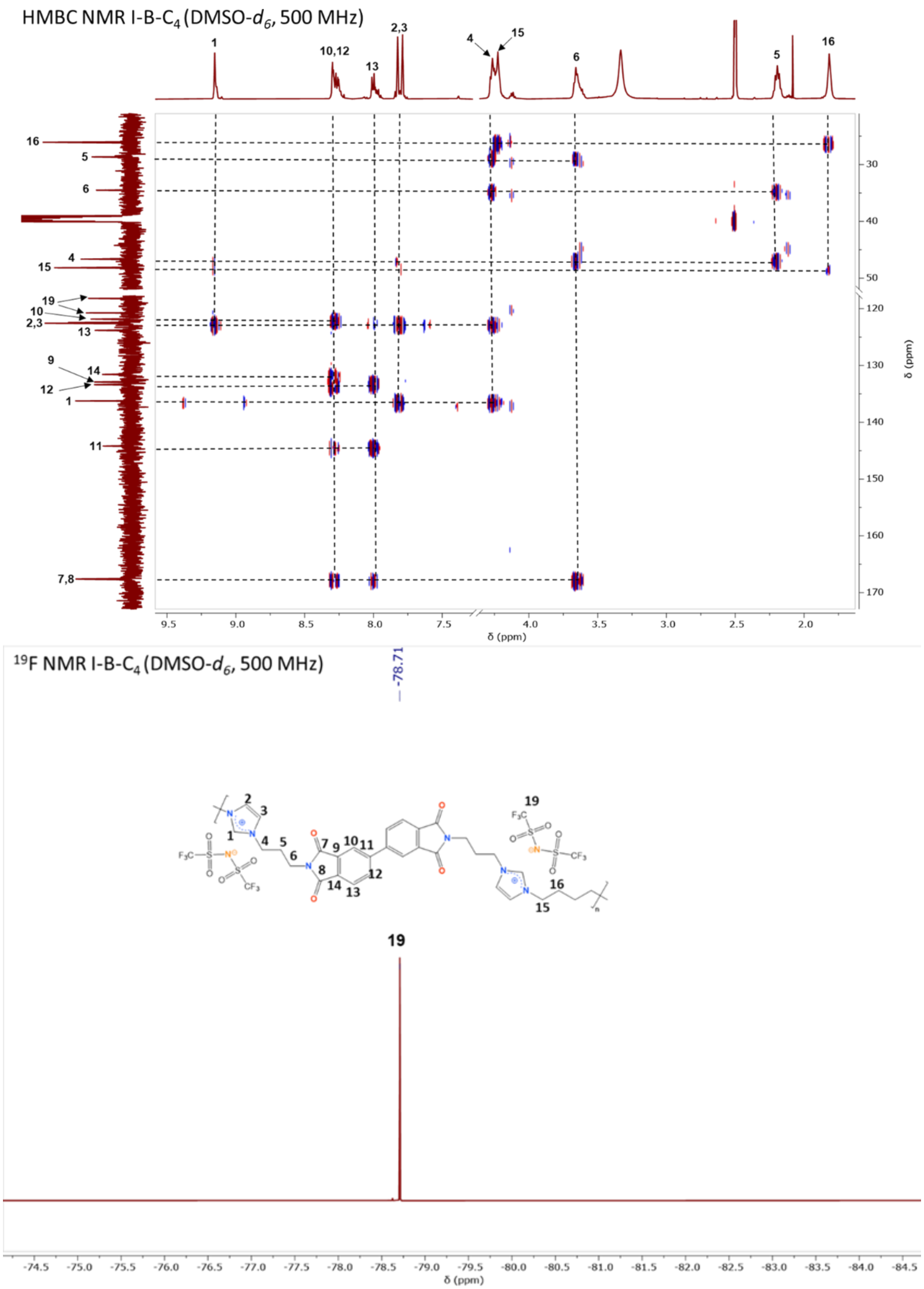

Figure S8: FT-IR-ATR and ^{1}H, ^{13}C, DEPT 135°, COSY, HSQC, HMBC and ^{19}F NMR spectra of ionene B-C_4

B-C$_8$ [BPDA-API-C$_8$] [NTf$_2$]

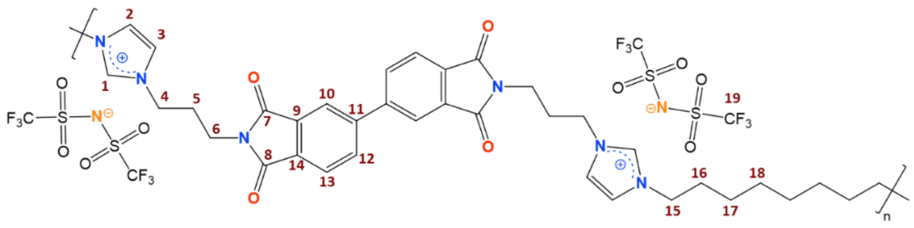

B-C$_8$ spectroscopic data. Yield: 97 %. IR (KBr, cm^{-1}): 3150, 3116, 3093 (C-H, arom.); 2939, 2862 (C-H, aliph); 1770, 1707 (C=O); 1622, 1563 (C=C); 1442 (C=N); 1394, 1177 (C-N); 1345, 1132 (SO_2); 1228 (CF_3); 1051 (S-N-S); 739 (subts. imide ring). ^{1}H NMR (DMSO-*d*$_6$, δ, ppm): 9.15 (m, 2H, **1**); 8.30 (m, 4H, **10,12**); 8.00 (m, 2H, **13**); 7.80 (m, 4H, **2,3**); 4.26 (t, *J* = 7.4 Hz, 4H, **4**); 4.15 (t, *J* = 7.4 Hz, 4H, **15**); 3.66 (t, *J* = 6.6 Hz, 4H, **6**); 2.19 (q, *J* = 7.3 Hz, 4H, **5**); 1.79 (q, *J* = 6.6 Hz, 4H, **16**); 1.27 (m, 8H, **17,18**). ^{13}C NMR (DMSO-*d*$_6$, δ, ppm): 167.62, 167.60 (**7,8**); 144.18 (**11**); 136.09 (**1**); 133.38 (**12**); 133.92 (**9**); 131.60 (**14**); 123.82 (**13**); 122.45 (**2,3**); 121.84 (**10**); 120.75, 118.19 (**19**); 48.89 (**15**); 46.62 (**4**), 34.53 (**6**), 29.33 (**16**); 28.64 (**5**), 28.26 (**17**); 26.52 (**18**). ^{19}F NMR (DMSO-*d*$_6$, δ, ppm): -78.71 (**19**).

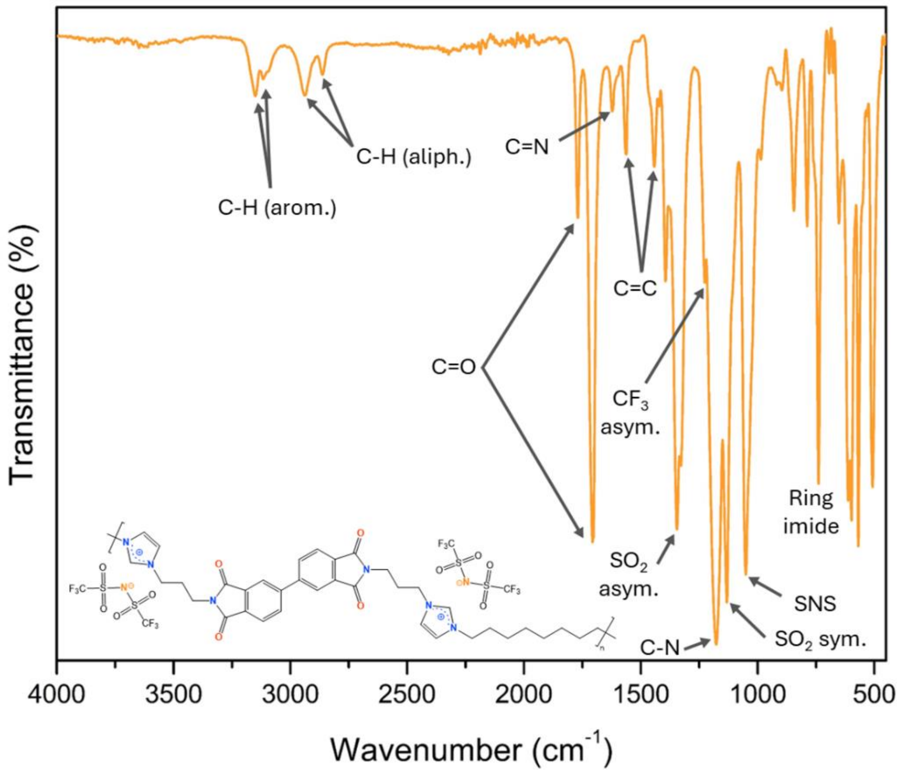

^{1}H NMR I-B-C$_8$ (DMSO-d_6, 500 MHz)

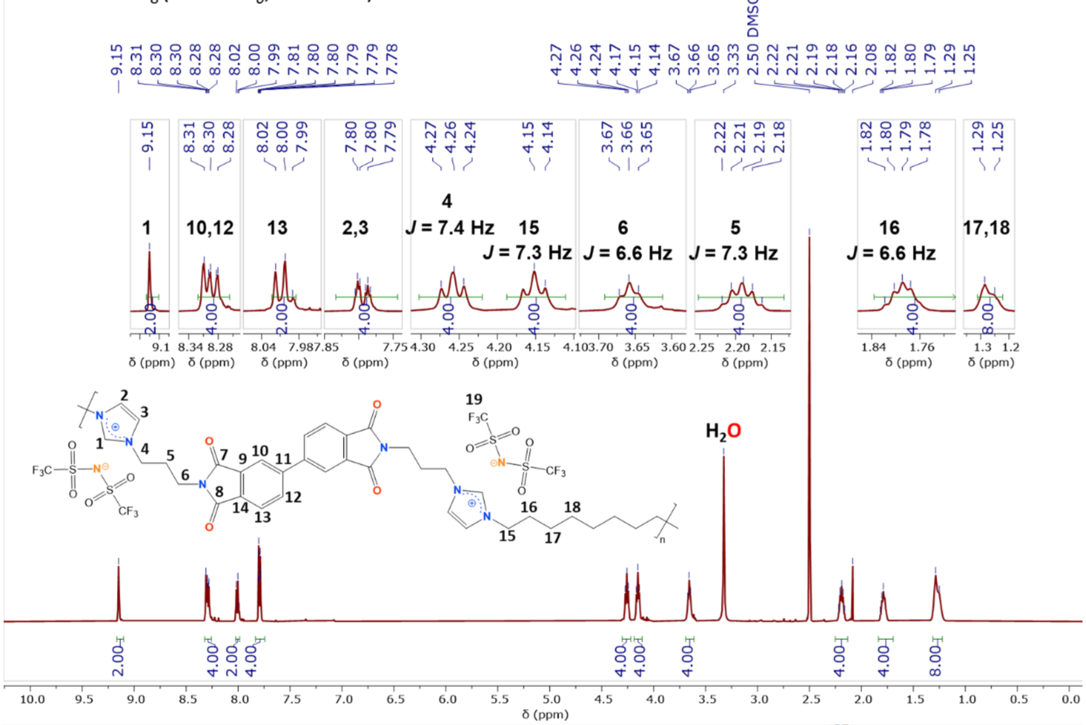

^{13}C NMR I-B-C$_8$ (DMSO-d_6, 500 MHz)

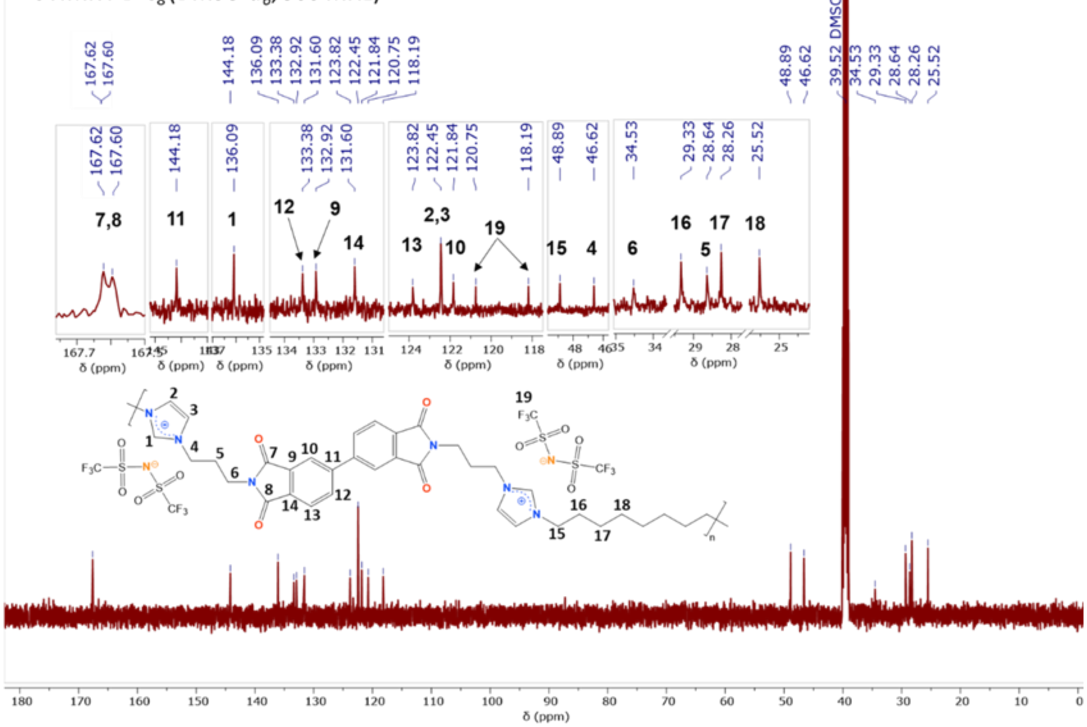

^{13}C/Dept 45° NMR I-B-C_8 (DMSO-d_6, 500 MHz)

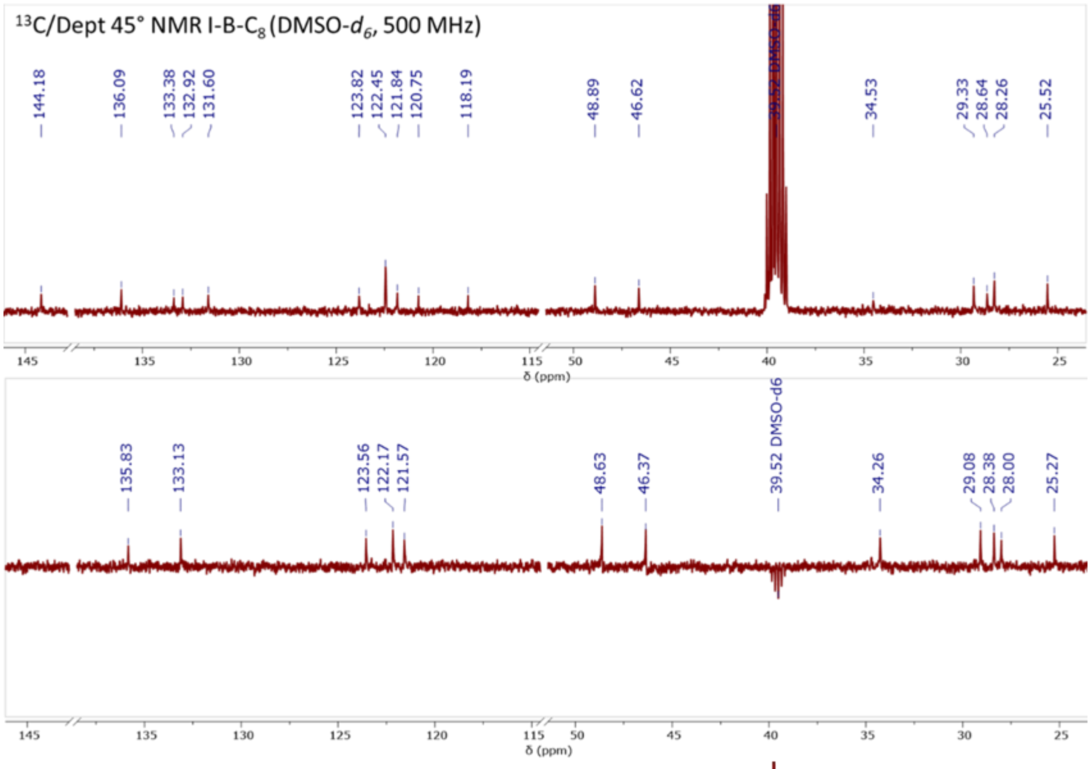

COSY NMR I-B-C_8 (DMSO-d_6, 500 MHz)

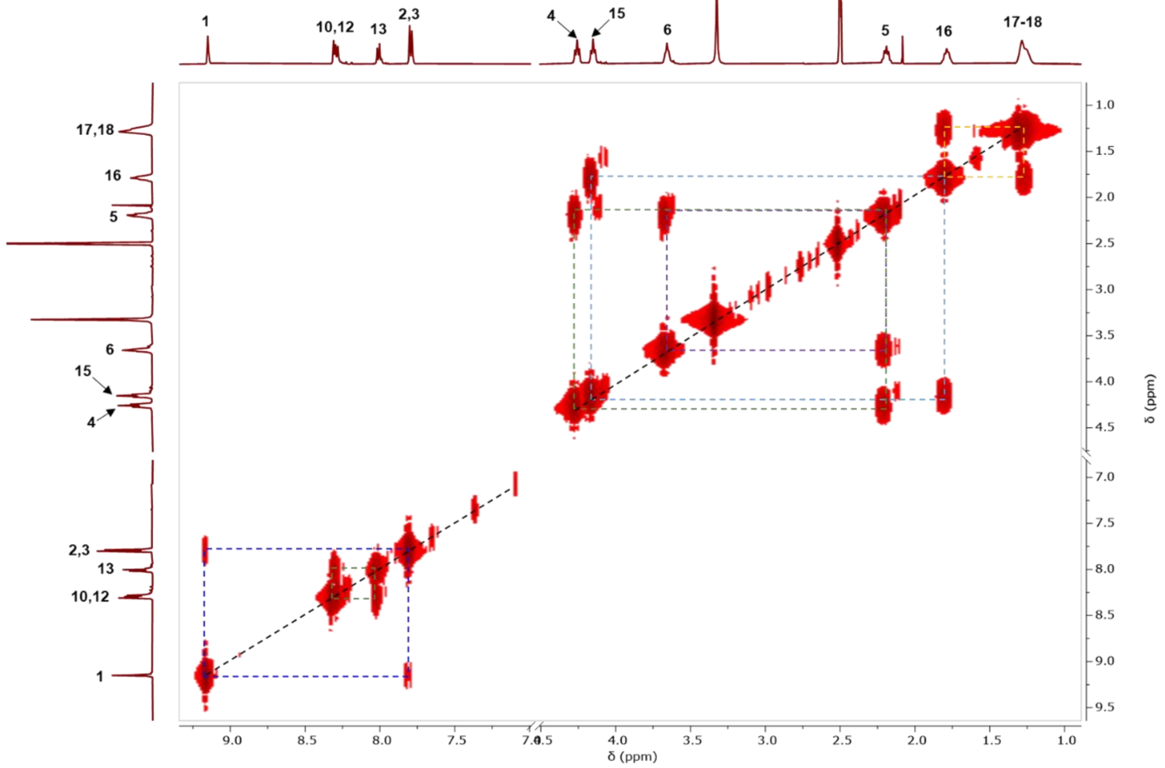

HSQC NMR I-B-C_8 (DMSO-d_6, 500 MHz)

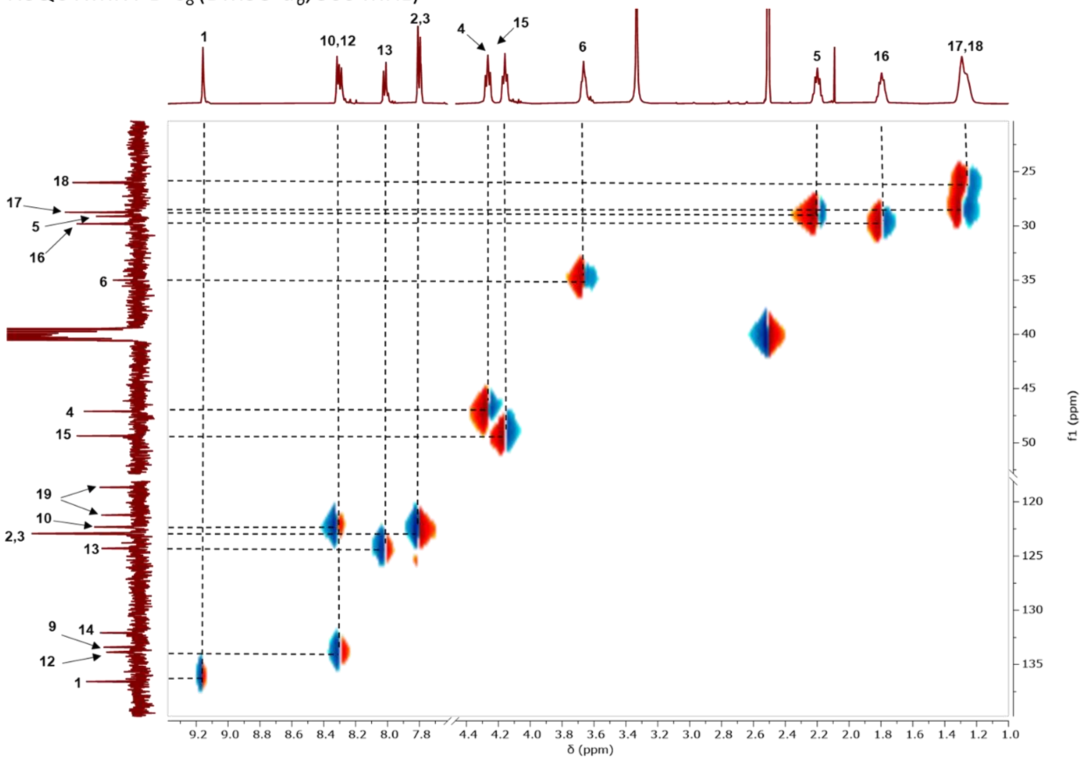

HMBC NMR I-B-C_8 (DMSO-d_6, 500 MHz)

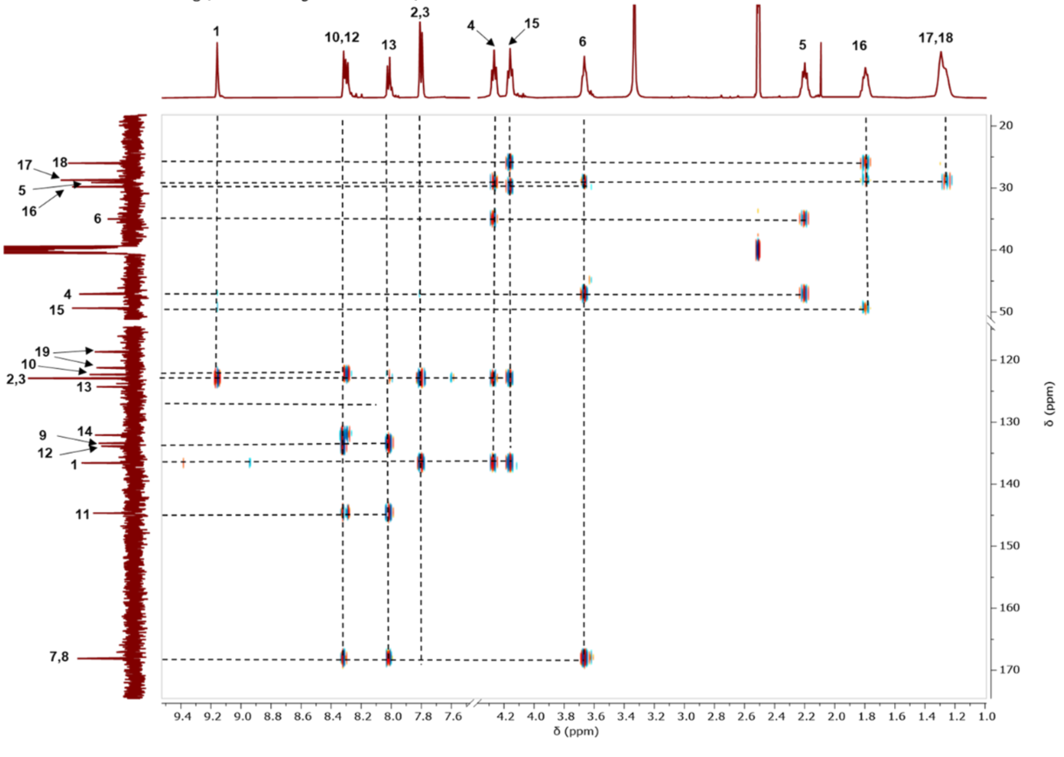

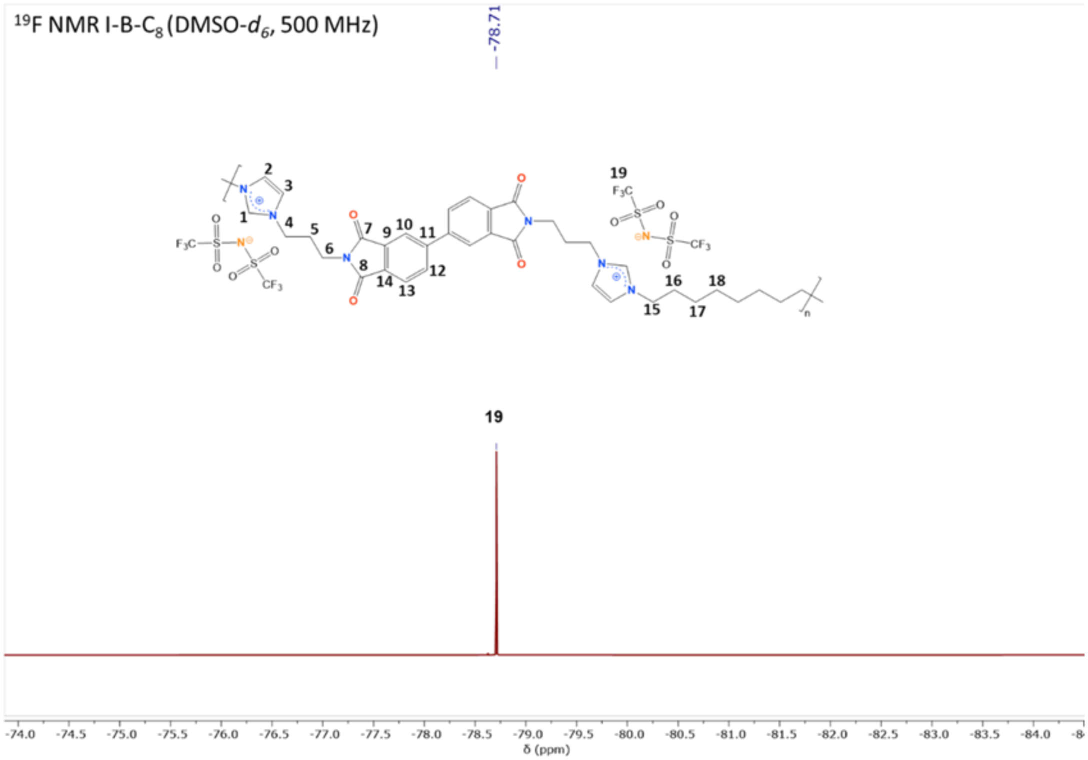

Figure S9: FT-IR-ATR and ^{1}H, ^{13}C, DEPT 45°, COSY, HSQC, HMBC and ^{19}F NMR spectra of ionene B-C_8.

B-C_{12} [BPDA-API-C_{12}] [NTf_2]

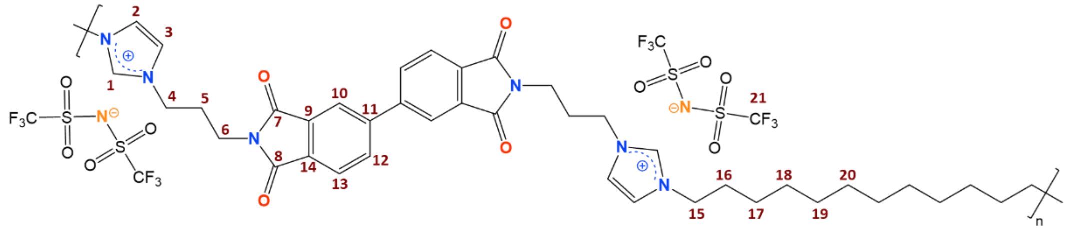

B-C_{12} spectroscopic data. Yield: 94 %. IR (KBr, cm^{-1}): 3150, 3114, 3091 (C-H, arom.); 2925, 2854 (C-H, aliph); 1770, 1707 (C=O); 1621, 1564 (C=C); 1442 (C=N); 1394, 1179 (C-N); 1347, 1130 (SO_2); 1227 (CF_3); 1051 (S-N-S); 739 (subts. imide ring). ^{1}H NMR (DMSO-d_6, δ, ppm): 9.15 (m, 2H, **1**); 8.30 (m, 4H, **10,12**); 8.00 (m, 2H, **13**); 7.79 (m, 4H, **2,3**); 4.26 (t, J = 7.3 Hz, 4H, **4**); 4.15 (t, J = 7.2 Hz, 4H, **15**); 3.66 (t, J = 5.9 Hz, 4H, **6**); 2.19 (q, J = 6.6 Hz, 4H, **5**); 1.78 (m, 4H, **16**); 1.23 (m, 8H, **17-20**). ^{13}C NMR (DMSO-d_6, δ, ppm): 167.61, 167.59 (**7,8**); 144.17 (**11**); 136.10 (**1**); 133.39 (**12**); 133.92 (**9**); 131.59 (**14**); 123.82 (**13**); 122.43 (**2,3**); 121.84 (**10**); 120.75, 118.19 (**21**); 48.90 (**15**); 46.62 (**4**), 34.52 (**6**), 29.36 (**16**); 29.02 (**17**); 28.92 (**18**); 28.61 (**19**); 28.46 (**5**), 25.58 (**18**). ^{19}F NMR (DMSO-d_6, δ, ppm): -78.71 (**21**).

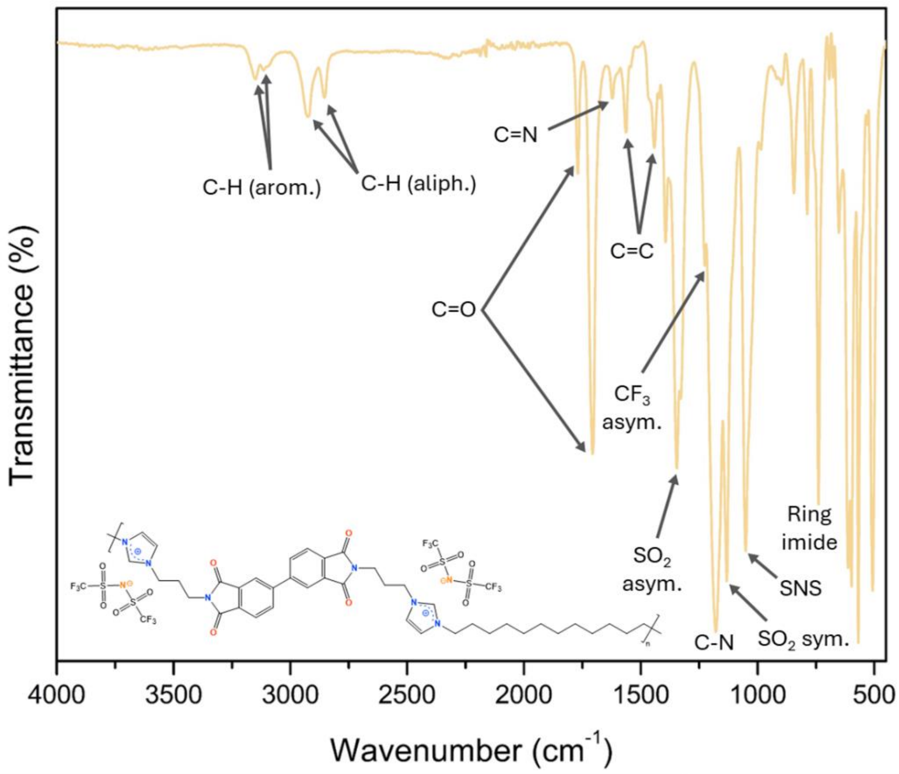
Transmittance (%)
Wavenumber (cm^{-1})
C-H (arom.)
C-H (aliph.)
C=N
C=C
C=O
CF_3 asym.
SO_2 asym.
C-N
SO_2 sym.
SNS
Ring imide
4000
3500
3000
2500
2000
1500
1000
500

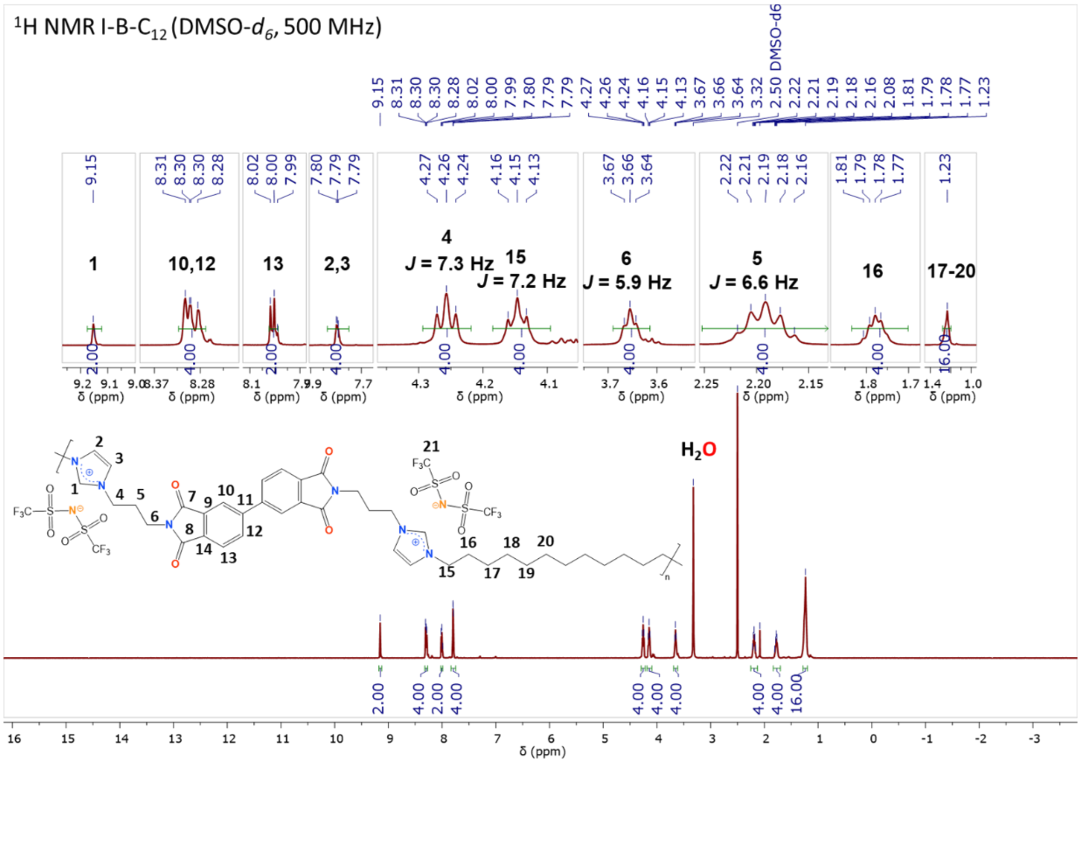
^{1}H NMR I-B-C_{12} (DMSO-d_6, 500 MHz)
1
10,12
13
2,3
4 J = 7.3 Hz
15 J = 7.2 Hz
6 J = 5.9 Hz
5 J = 6.6 Hz
16
17-20
H_2O
DMSO-d6
δ (ppm)

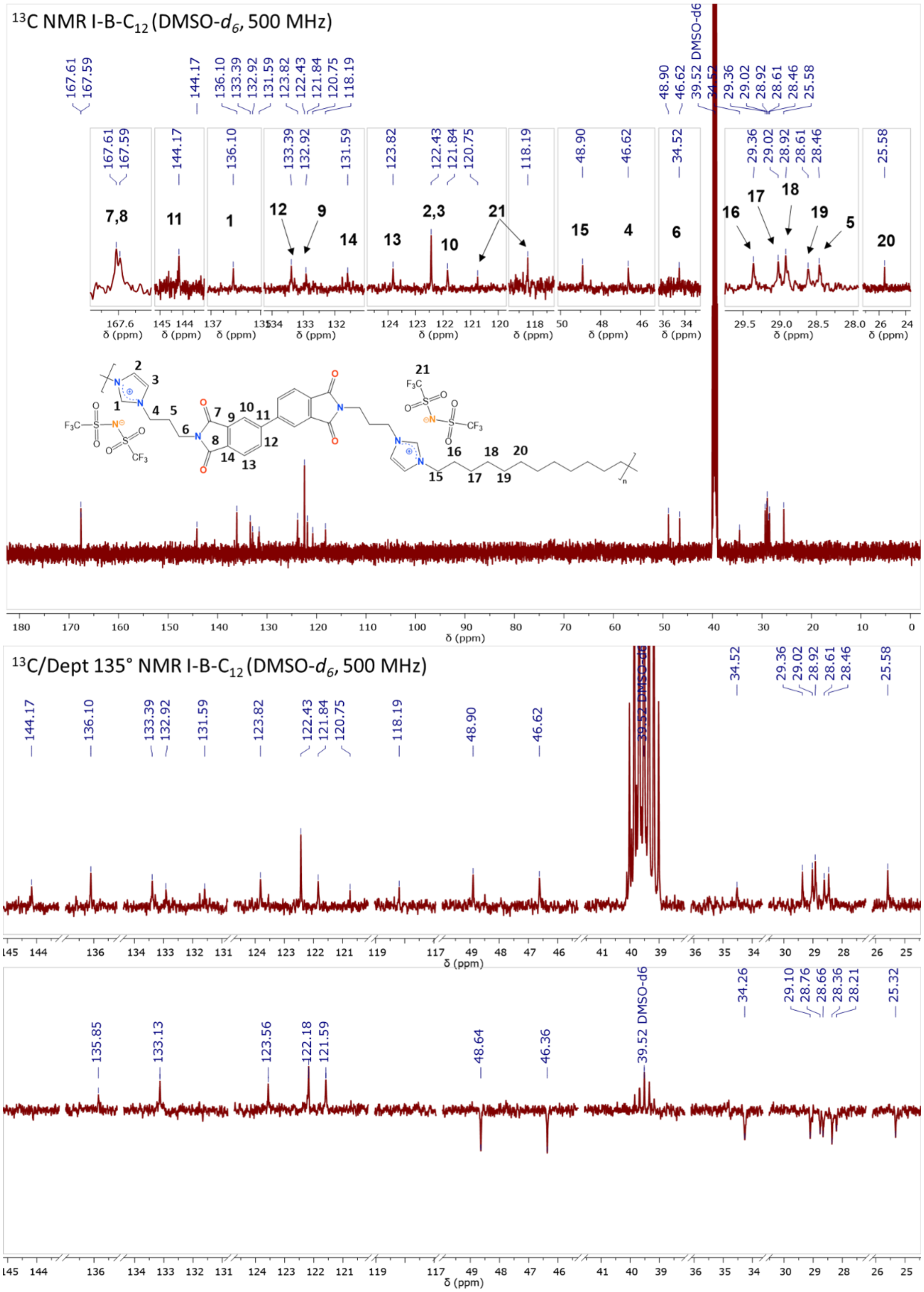

13C NMR I-B-C12 (DMSO-d6, 500 MHz)
167.61 167.59 144.17 136.10 133.39 132.92 131.59 123.82 122.43 121.84 120.75 118.19
48.90 46.62 39.52 DMSO-d6 34.52 29.36 29.02 28.92 28.61 28.46 25.58
7,8 11 1 12 9 14 13 2,3 10 21 15 4 6 16 17 18 19 5 20
δ (ppm)
13C/Dept 135° NMR I-B-C12 (DMSO-d6, 500 MHz)
144.17 136.10 133.39 132.92 131.59 123.82 122.43 121.84 120.75 118.19 48.90 46.62 39.52 DMSO-d6 34.52 29.36 29.02 28.92 28.61 28.46 25.58
135.85 133.13 123.56 122.18 121.59 48.64 46.36 39.52 DMSO-d6 34.26 29.10 28.76 28.66 28.36 28.21 25.32
δ (ppm)

COSY NMR I-B-C_{12} (DMSO-d_6, 500 MHz)

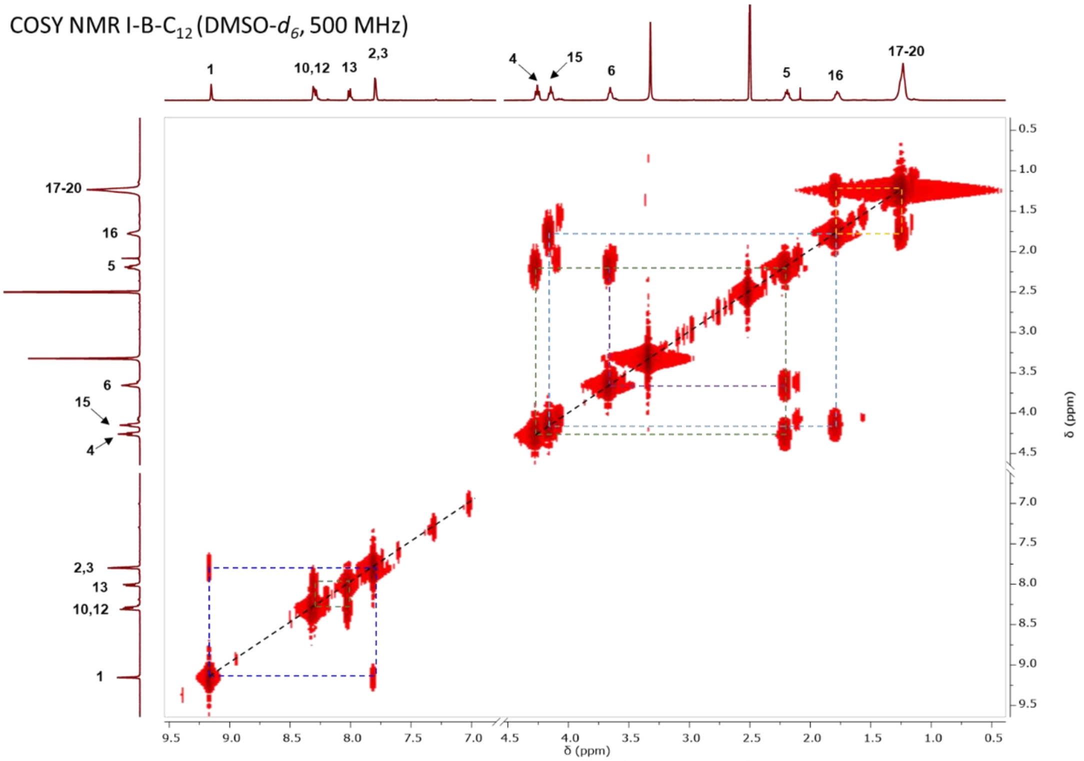

HSQC NMR I-B-C_{12} (DMSO-d_6, 500 MHz)

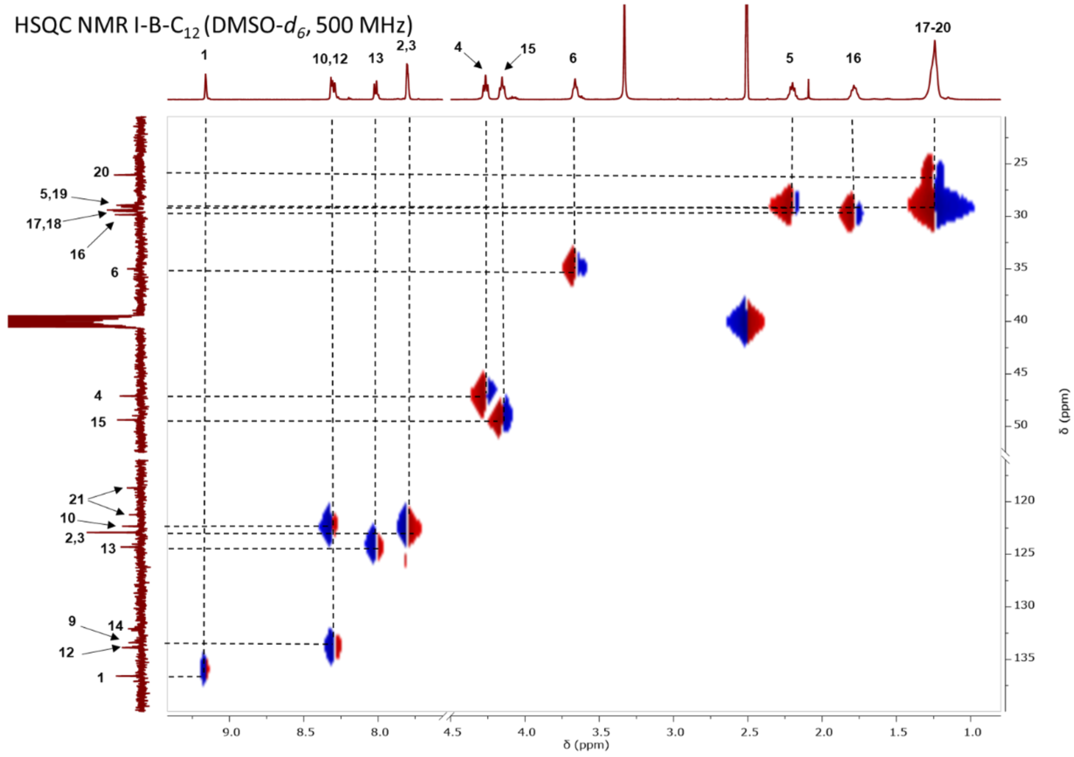

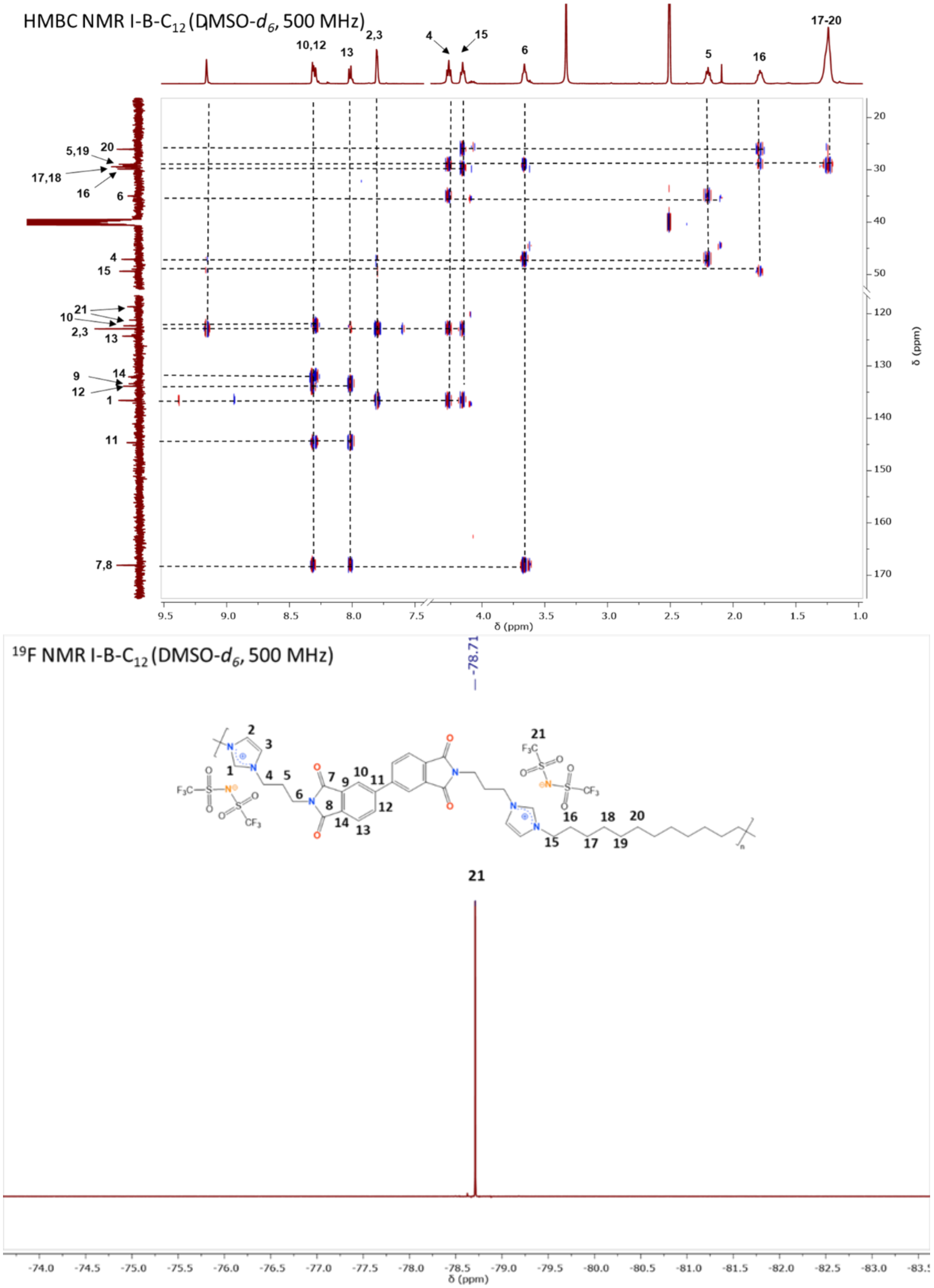

Figure S10: FT-IR-ATR and ^{1}H, ^{13}C, DEPT 135°, COSY, HSQC, HMBC and ^{19}F NMR spectra of ionene B-C_{12}.

O-C_4 [ODPA-API-C_4] [NTf_2]

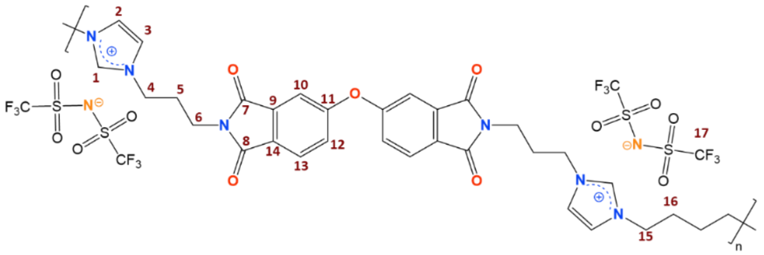

O-C_4 spectroscopic data. Yield: 92 %. IR (KBr, cm^{-1}): 3151, 3116 (C-H, arom.); 2926, 2852 (C-H, aliph); 1772, 1707 (C=O); 1610, 1562 (C=C); 1443 (C=N); 1396, 1173 (C-N); 1346, 1131 (SO_2); 1229 (CF_3); 1050 (S-N-S); 740 (subts. imide ring). ^{1}H NMR (DMSO-d_6, δ, ppm): 9.14 (m, 2H, **1**); 7.97 (d, J = 8.3 Hz, 2H, **13**); 7.81, 7.78 (m/m, 2H/2H, **2,3**); 7.53 (m, 4H, **10,12**); 4.24 (m, 8H, **4,15**); 3.61 (t, J = 6.3 Hz, 4H, **6**); 2.16 (q, J = 6.3 Hz, 4H, **5**); 1.81 (m, 4H, **16**). ^{13}C NMR (DMSO-d_6, δ, ppm): 167.29 (**8**), 167.07 (**7**); 160.60 (**11**); 136.23 (**1**); 134.66 (**9**); 127.36 (**14**); 125.66 (**13**); 124.35 (**12**); 122.54, 122.42 (**2,3**); 120.75, 118.19 (**17**); 113.55 (**10**); 48.14 (**15**); 46.60 (**4**); 34.47 (**6**), 28.60 (**5**); 26.06 (**16**). ^{19}F NMR (DMSO-d_6, δ, ppm): -78.71 (**17**).

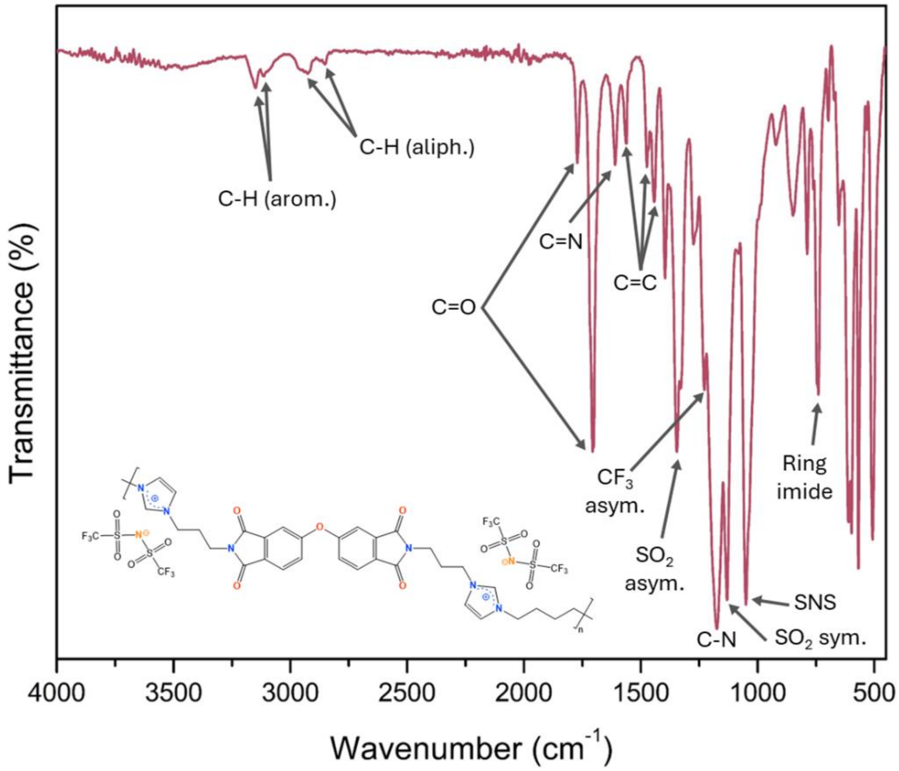

^{1}H NMR I-O-C_4 (DMSO-d_6, 500 MHz)

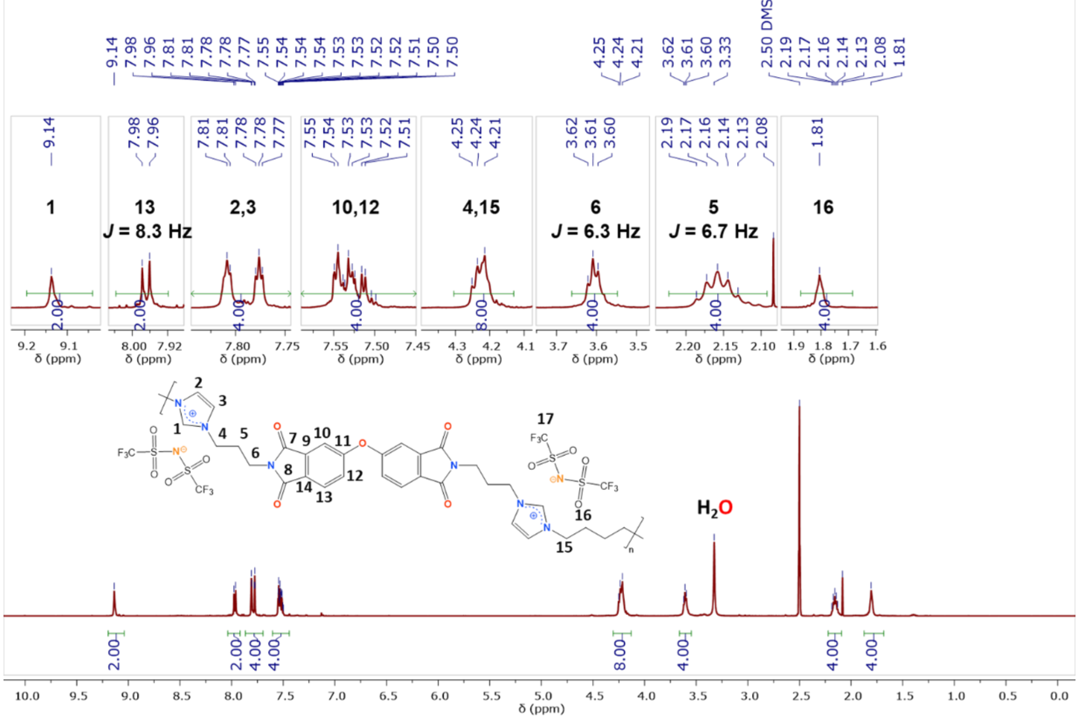

^{13}C NMR I-O-C_4 (DMSO-d_6, 500 MHz)

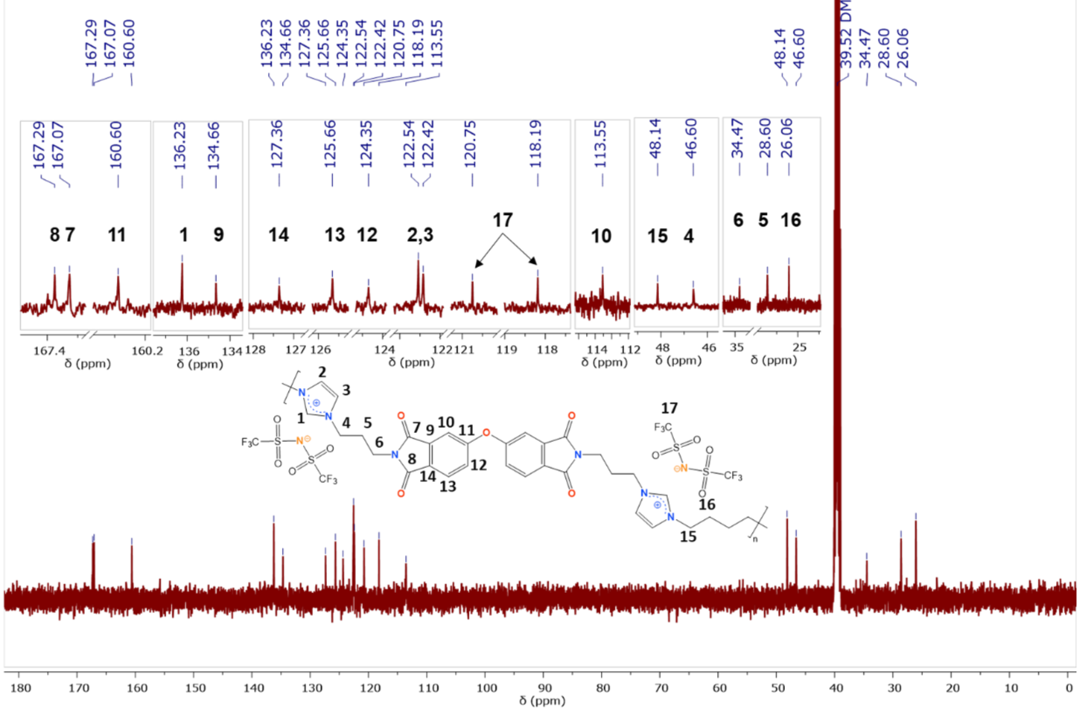

^{13}C/Dept 135° NMR I-O-C_4 (DMSO-d_6, 500 MHz)

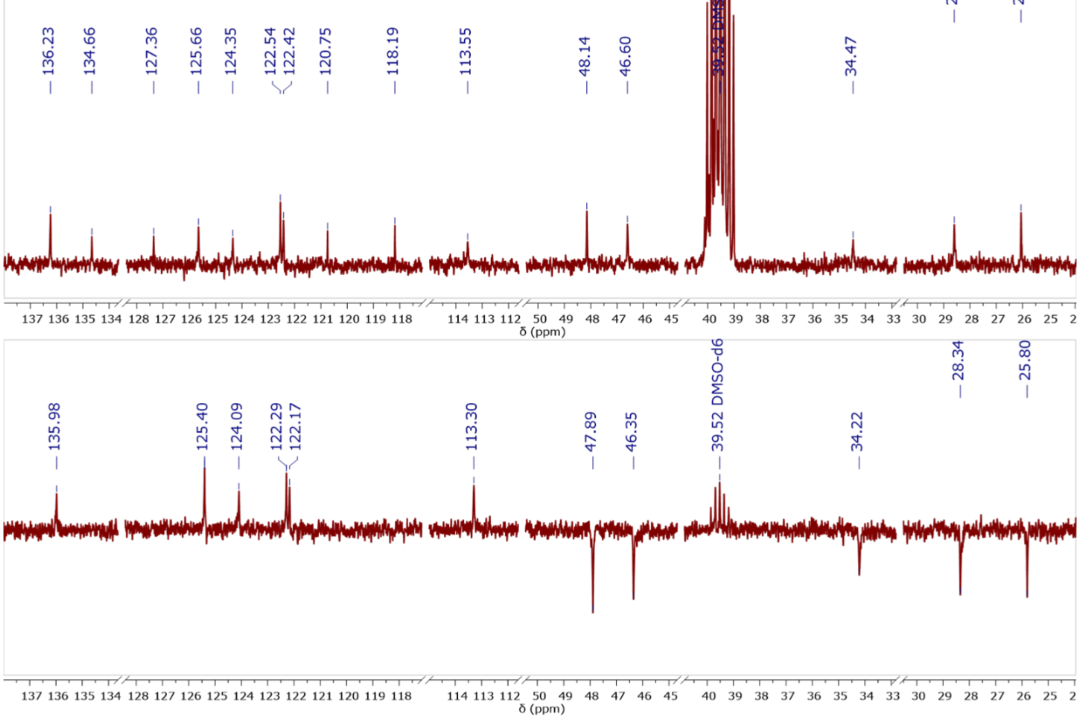

COSY 135° NMR I-O-C_4 (DMSO-d_6, 500 MHz)

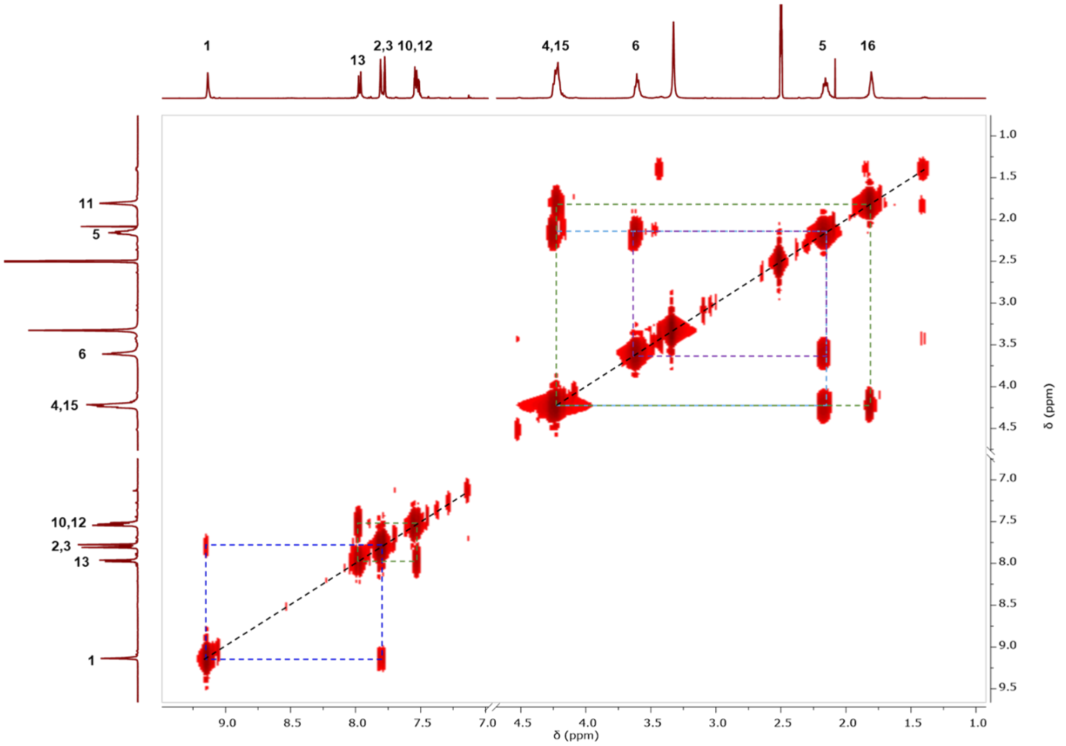

HSQC NMR I-O-C_4 (DMSO-d_6, 500 MHz)

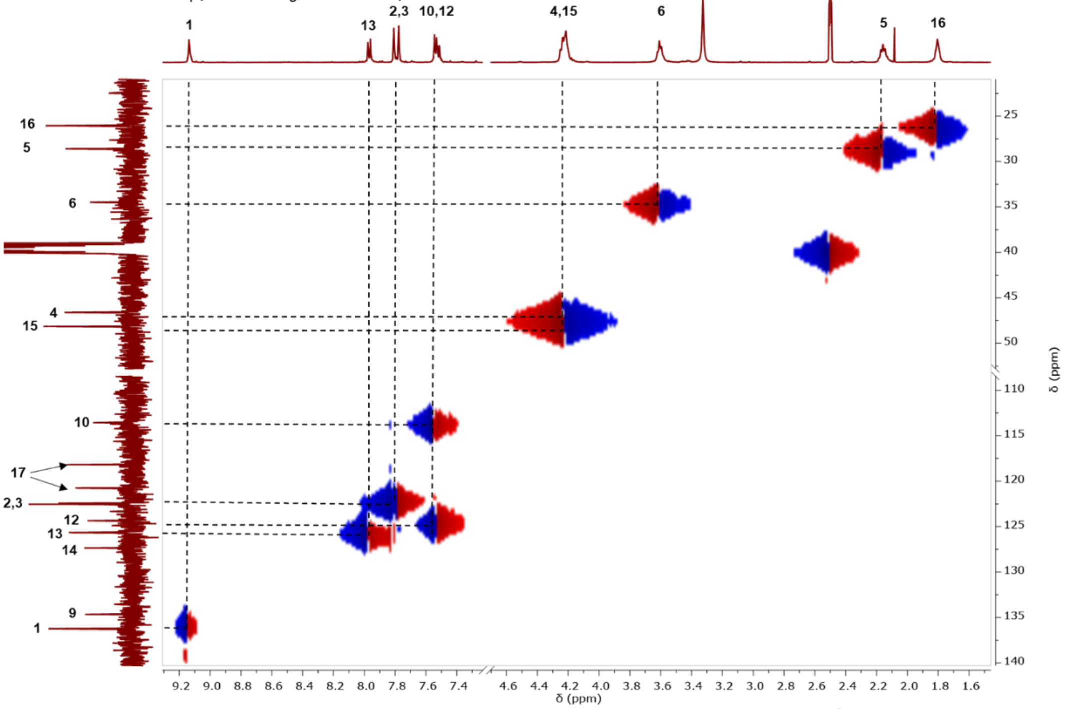

HMBC NMR I-O-C_4 (DMSO-d_6, 500 MHz)

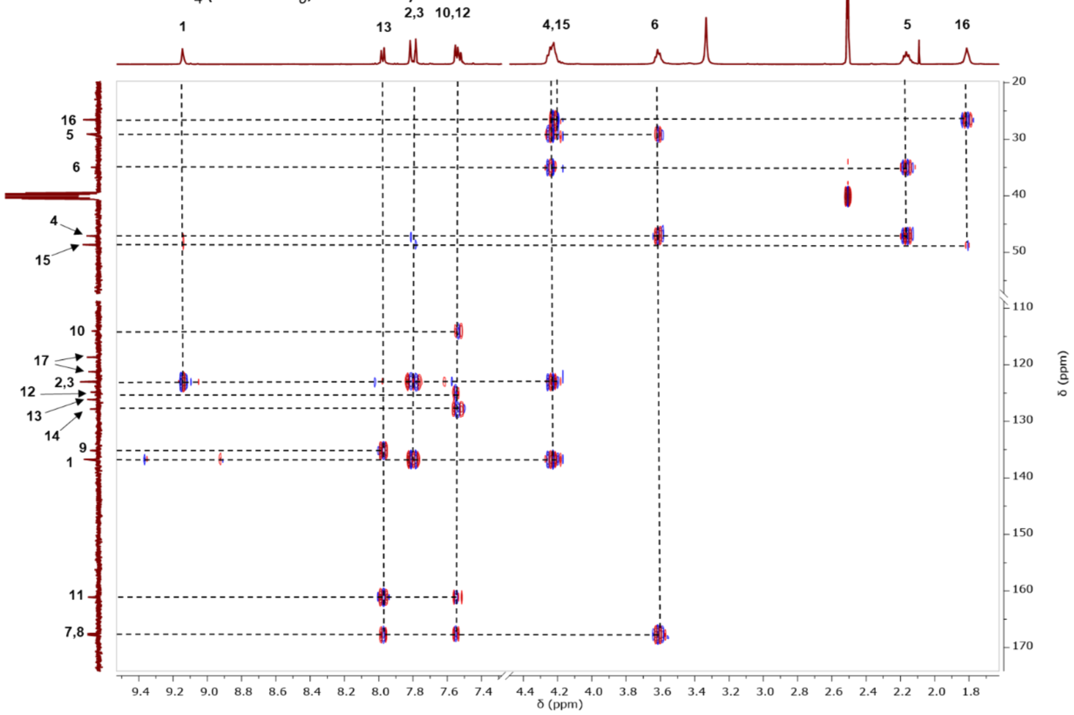

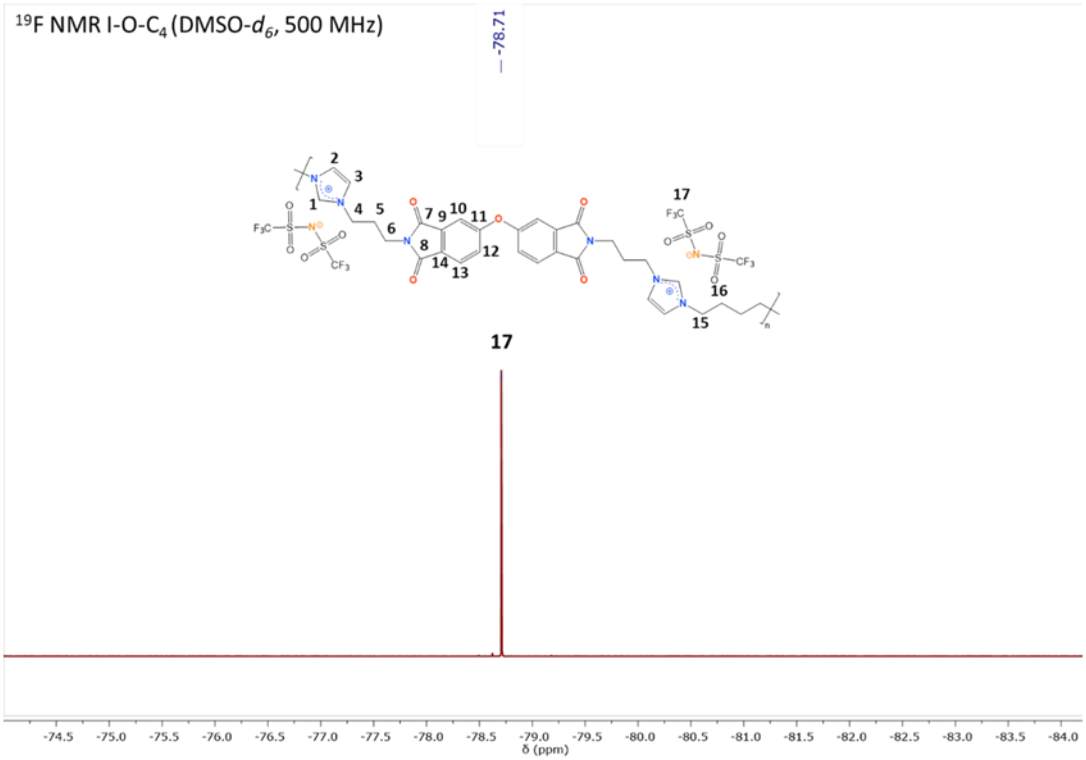

Figure S11: FT-IR-ATR and ^{1}H, ^{13}C, DEPT 135°, COSY, HSQC, HMBC and ^{19}F NMR spectra of ionene O-C4.

O-C8 [ODPA-API-C8] [NTf2]

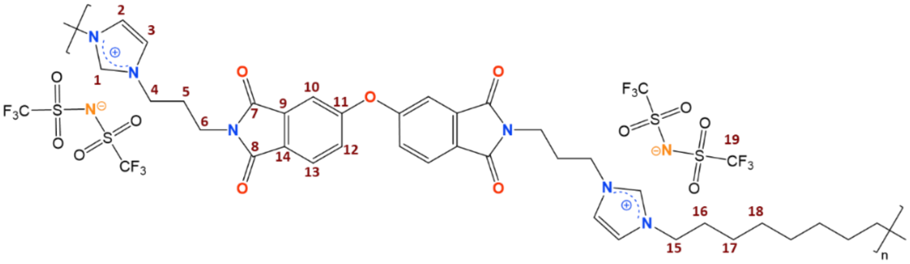

O-C8 spectroscopic data. Yield: 95 %. IR (KBr, cm^{-1}): 3150, 3114, 3091 (C-H, arom.); 2925, 2854 (C-H, aliph); 1770, 1707 (C=O); 1621, 1564 (C=C); 1442 (C=N); 1394, 1179 (C-N); 1347, 1130 (SO_2); 1227 (CF_3); 1051 (S-N-S); 739 (subts. imide ring). ^{1}H NMR (DMSO-*d*6, δ, ppm): 9.14 (m, 2H, **1**); 7.97 (d, *J* = 7.3 Hz, 2H, **13**); 7.78 (m, 4H, **2,3**); 7.53 (m, 4H, **10,12**); 4.23 (t, *J* = 7.3 Hz, 4H, **4**); 4.15 (t, *J* = 7.2 Hz, 4H, **15**); 3.61 (t, *J* = 6.3 Hz, 4H, **6**); 2.16 (q, *J* = 6.7 Hz, 4H, **5**); 1.78 (q, *J* = 7.4 Hz, 4H, **16**); 1.27 (m, 8H, **17,18**). ^{13}C NMR (DMSO-*d*6, δ, ppm): 167.27 (**8**), 167.07 (**7**); 160.59 (**11**); 136.09 (**1**); 134.67 (**9**); 127.37 (**14**); 125.65 (**13**); 124.37 (**12**); 122.44 (**2,3**); 120.75,

118.19 (**19**); 113.52 (**10**); 48.88 (**15**); 46.59 (**4**); 34.50 (**6**), 29.33 (**5**); 28.60 (**16**); 28.25 (**17**); 25.52 (**18**). ^{19}F NMR (DMSO-d_6, δ, ppm): -78.71 (**19**).

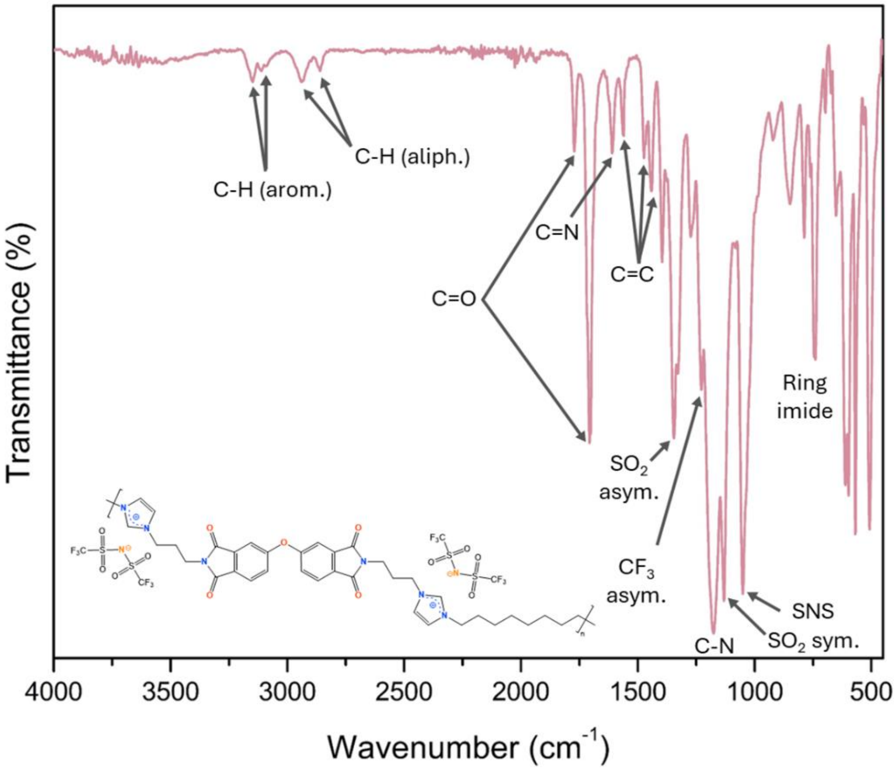

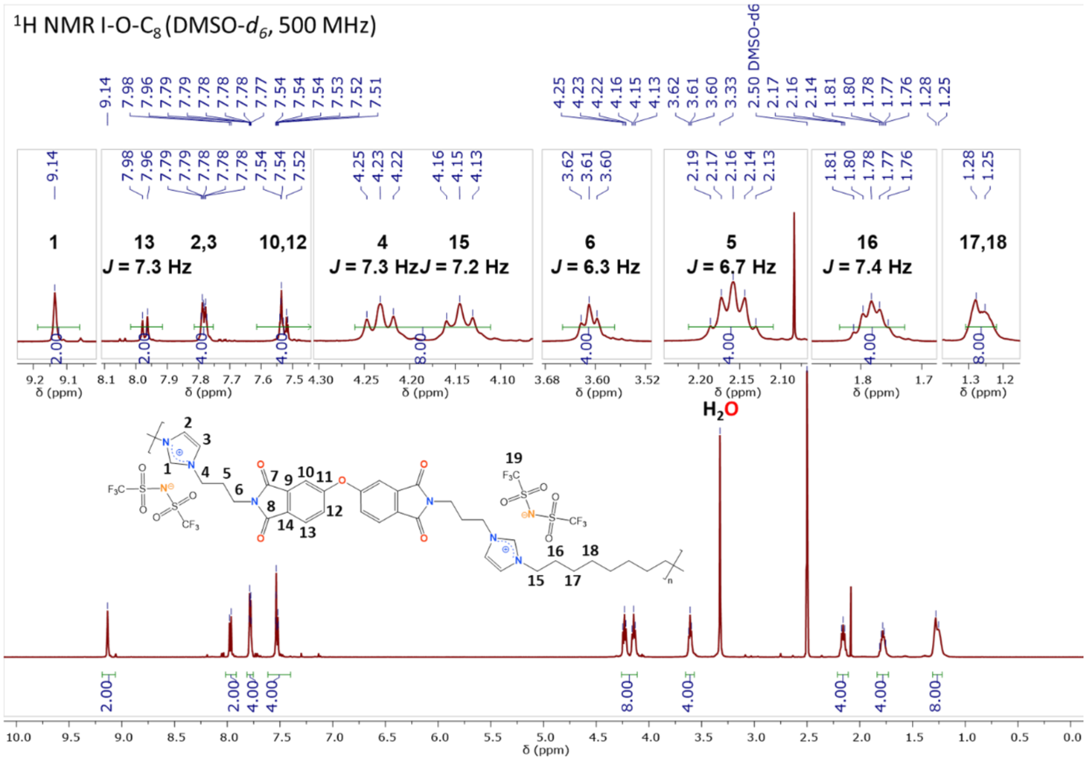

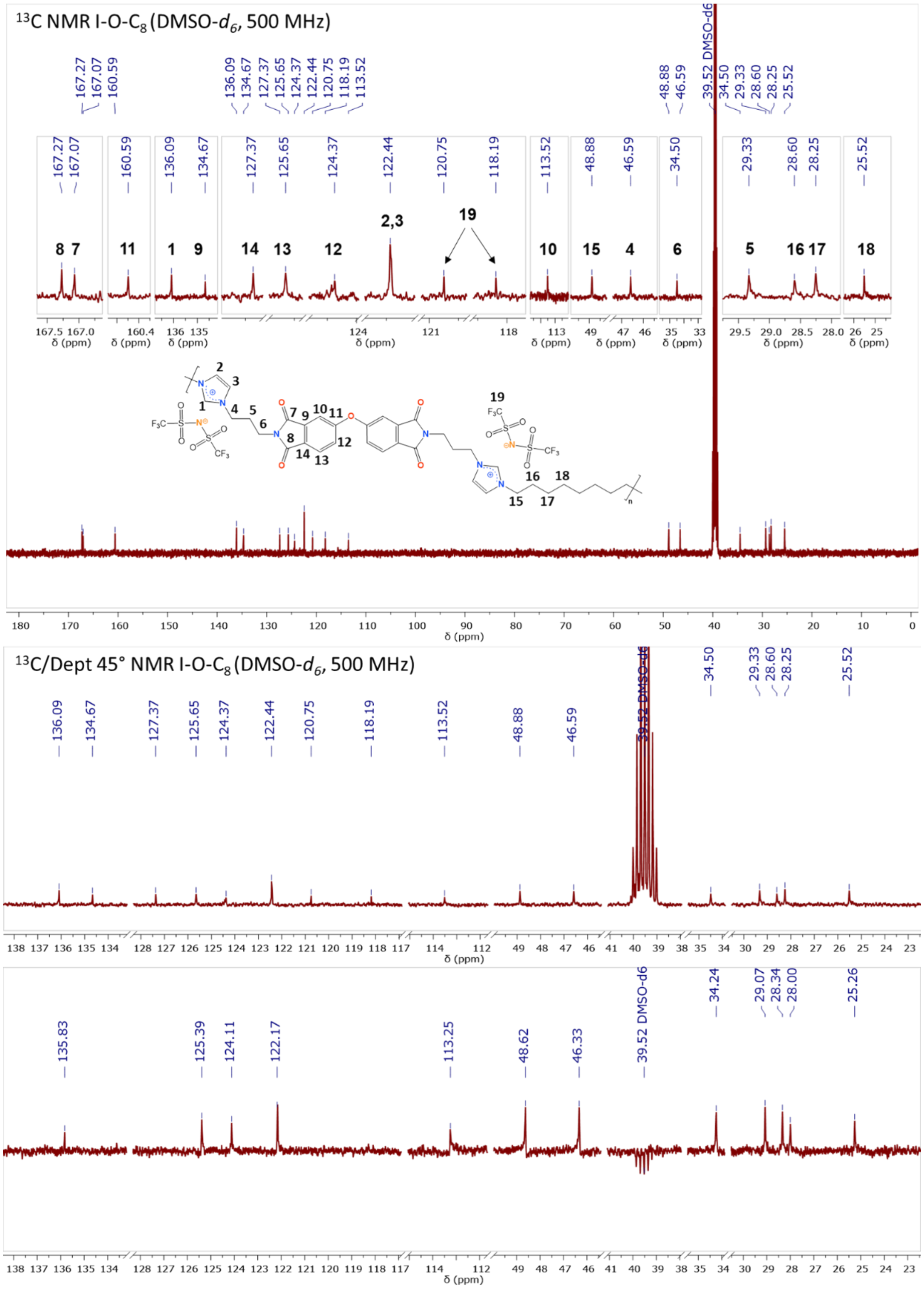
13C NMR I-O-C8 (DMSO-d6, 500 MHz)
167.27 167.07 160.59
136.09 134.67 127.37 125.65 124.37 122.44 120.75 118.19 113.52
48.88 46.59
39.52 DMSO-d6
34.50 29.33 28.60 28.25 25.52
δ (ppm)
13C/Dept 45° NMR I-O-C8 (DMSO-d6, 500 MHz)
136.09 134.67 127.37 125.65 124.37 122.44 120.75 118.19 113.52 48.88 46.59
34.50 29.33 28.60 28.25 25.52
135.83 125.39 124.11 122.17 113.25 48.62 46.33
39.52 DMSO-d6
34.24 29.07 28.34 28.00 25.26
δ (ppm)

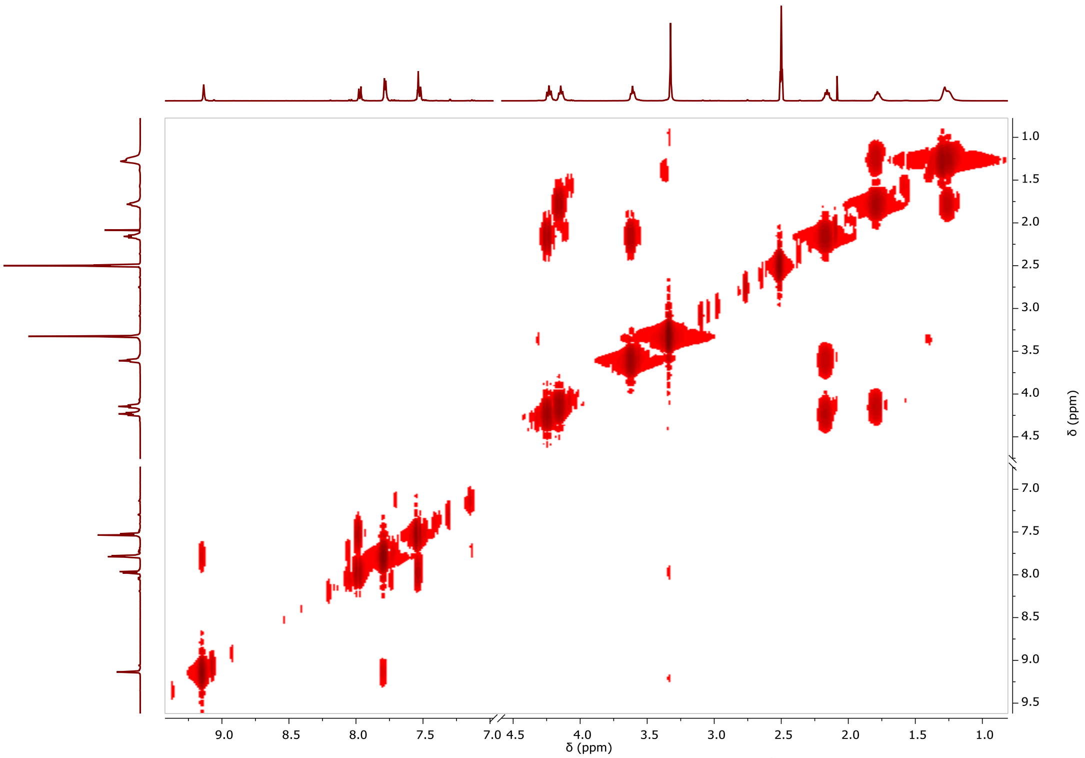

HSQC NMR I-O-C_8 (DMSO-d_6, 500 MHz)

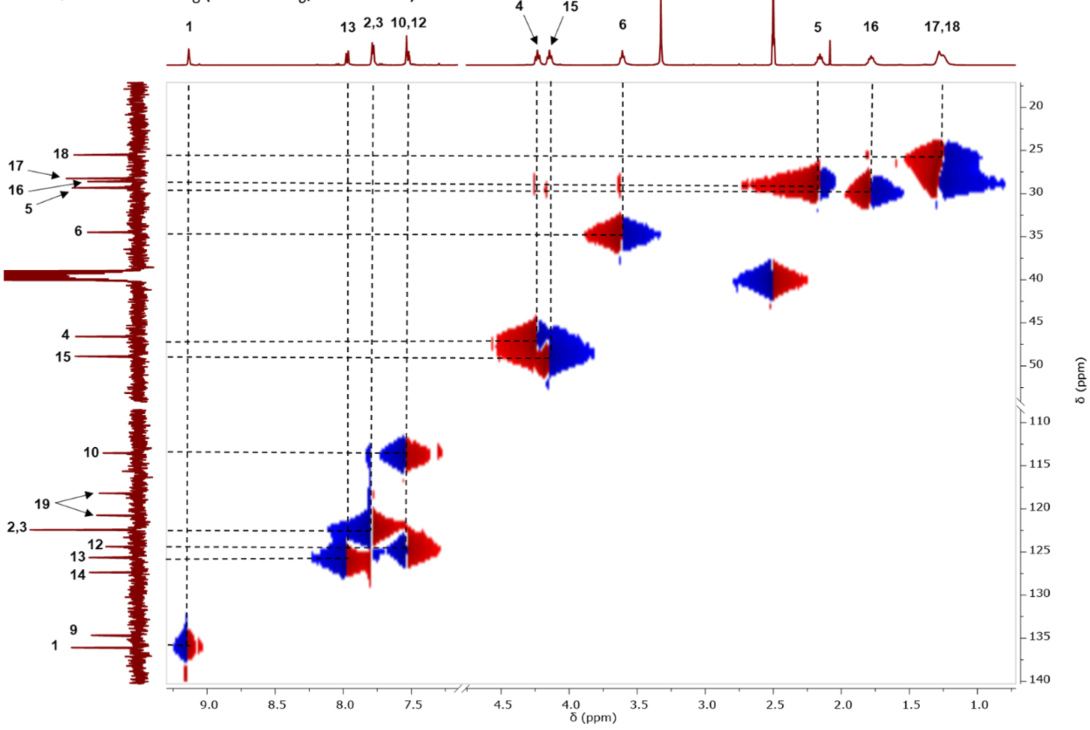

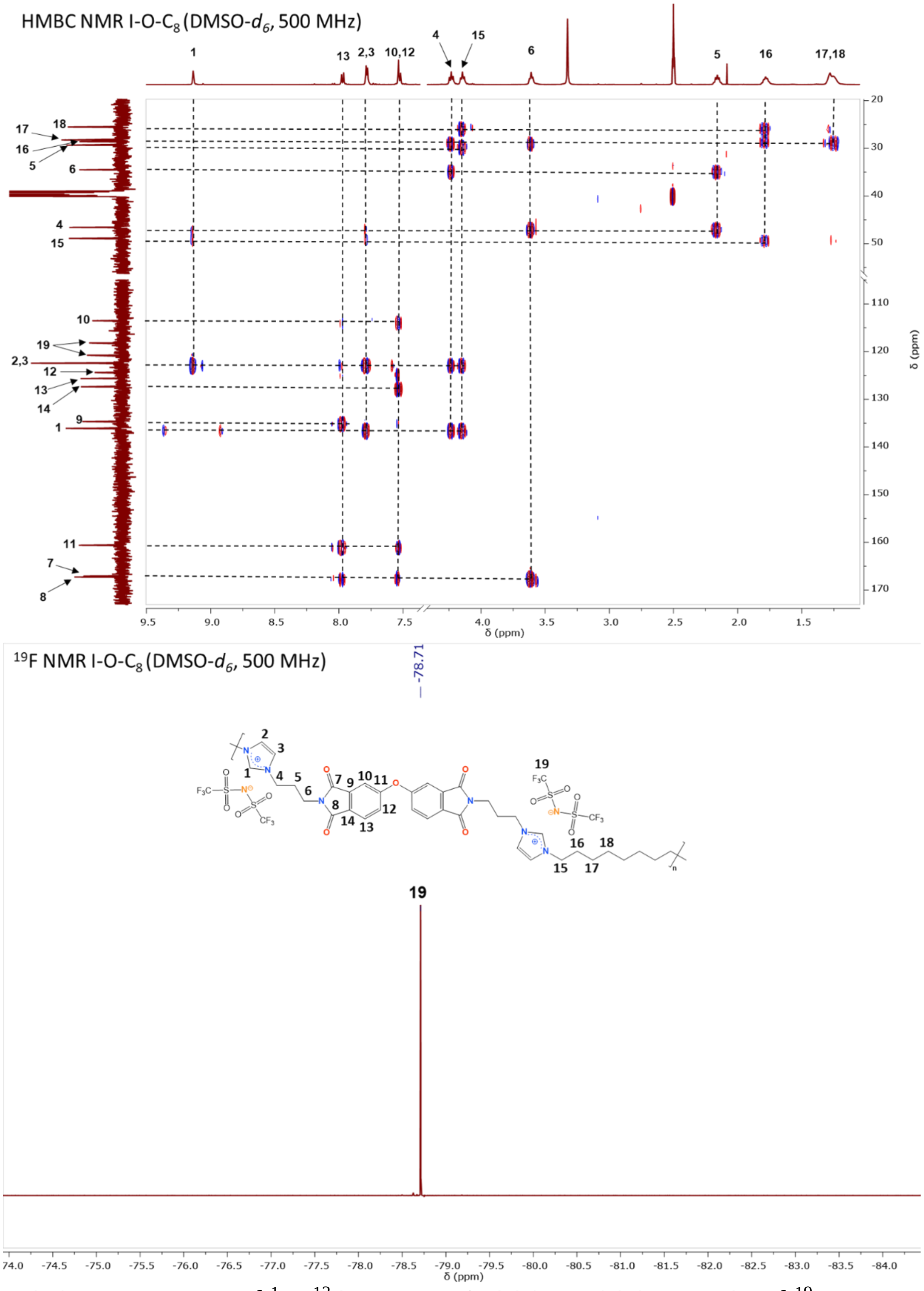

Figure S12: FT-IR-ATR and ^{1}H, ^{13}C, DEPT 45°, COSY, HSQC, HMBC and ^{19}F NMR spectra of ionene O-C_8.

O-C$_{12}$ [ODPA-API-C$_{12}$] [NTf_2]

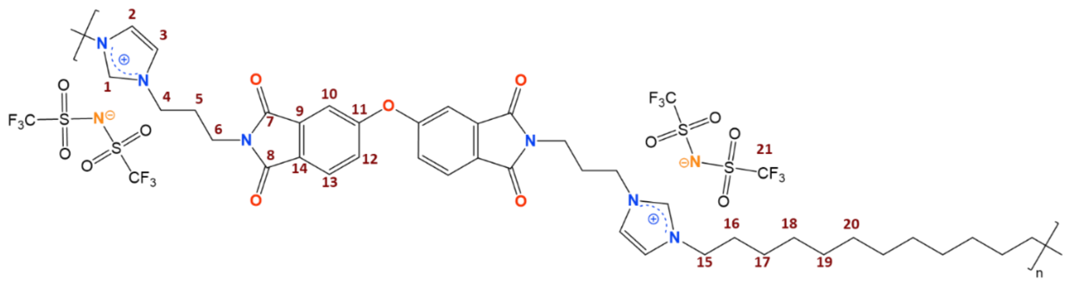

O-C$_{12}$ spectroscopic data. Yield: 95 %. IR (KBr, cm^{-1}): 3149, 3112 (C-H, arom.); 2930, 2857 (C-H, aliph); 1772, 1708 (C=O); 1610, 1562 (C=C); 1442 (C=N); 1396, 1177 (C-N); 1346, 1131 (SO_2); 1227 (CF_3); 1051 (S-N-S); 740 (subts. imide ring). ^{1}H NMR (DMSO-*d$_6$*, δ, ppm): 9.14 (m, 2H, **1**); 7.98 (m, 2H, **13**); 7.79 (m, 4H, **2,3**); 7.53 (m, 4H, **10,12**); 4.24 (t, *J* = 7.3 Hz, 4H, **4**); 4.15 (t, *J* = 7.2 Hz, 4H, **15**); 3.62 (t, *J* = 6.4 Hz, 4H, **6**); 2.17 (q, *J* = 6.7 Hz, 4H, **5**); 1.78 (m, 4H, **16**); 1.24 (m, 8H, **17-20**). ^{13}C NMR (DMSO-*d$_6$*, δ, ppm): 167.27 (**8**), 167.07 (**7**); 160.59 (**11**); 136.09 (**1**); 134.66 (**9**); 127.37 (**14**); 125.65 (**13**); 124.41 (**12**); 122.42 (**2,3**); 120.75, 118.19 (**21**); 113.51 (**10**); 48.90 (**15**); 46.59 (**4**); 34.50 (**6**), 29.36 (**5**); 29.02 (**16**); 28.92 (**17**); 28.58 (**18**); 28.46 (**19**); 25.58 (**20**). ^{19}F NMR (DMSO-*d$_6$*, δ, ppm): -78.71 (**21**).

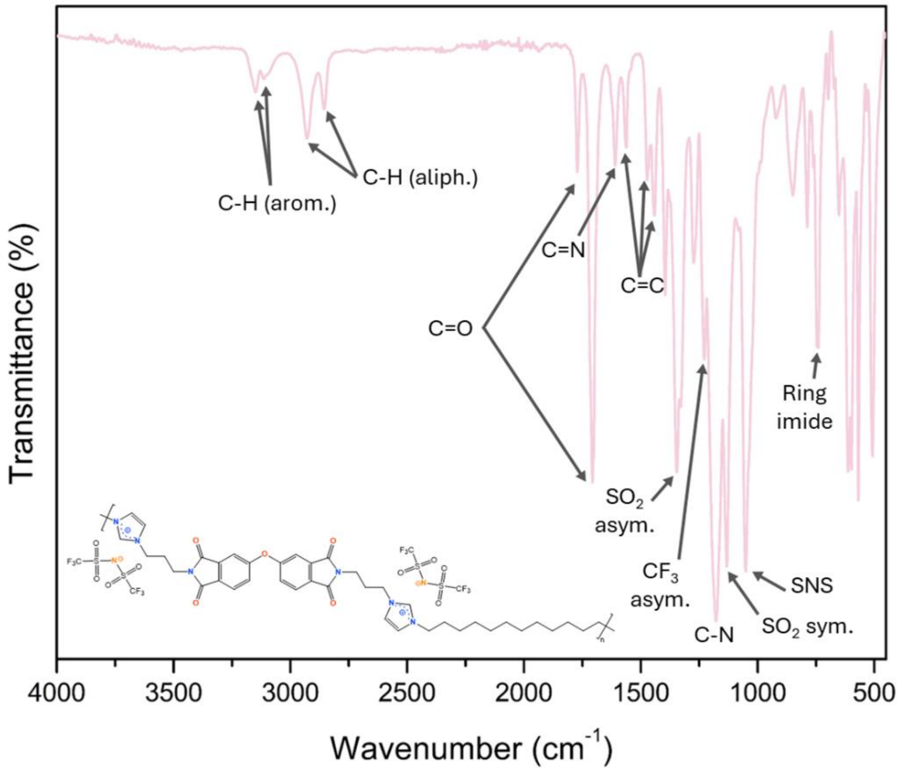

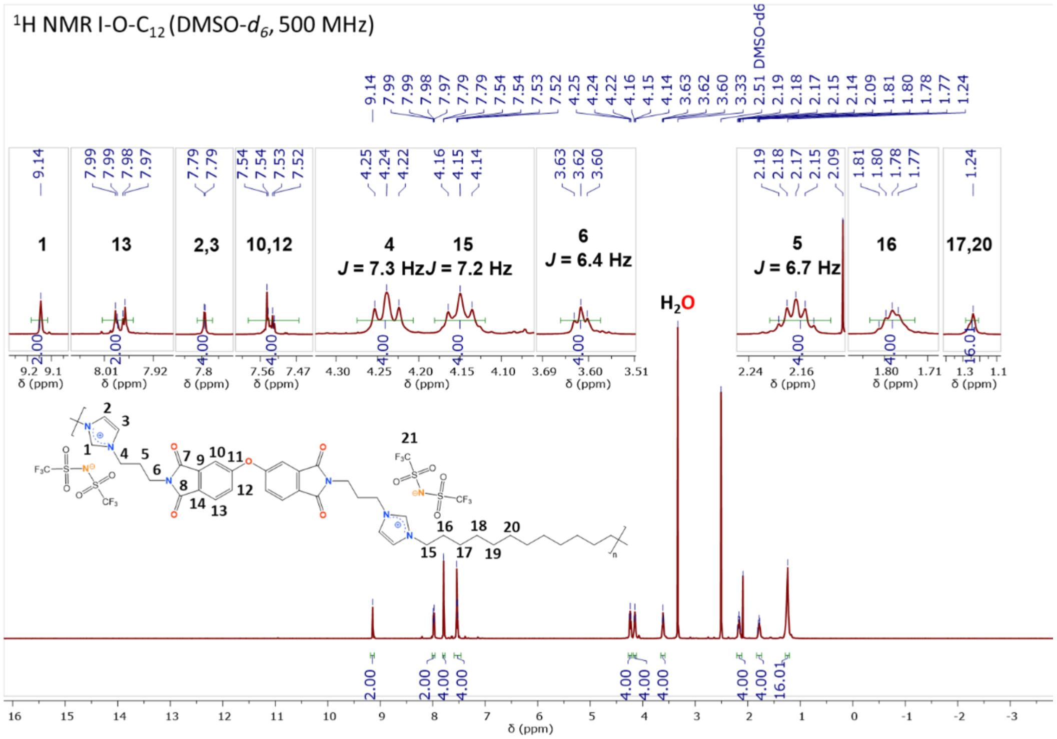
¹H NMR I-O-C12 (DMSO-d6, 500 MHz)
H2O
DMSO-d6
J = 7.3 Hz
J = 7.2 Hz
J = 6.4 Hz
J = 6.7 Hz
δ (ppm)

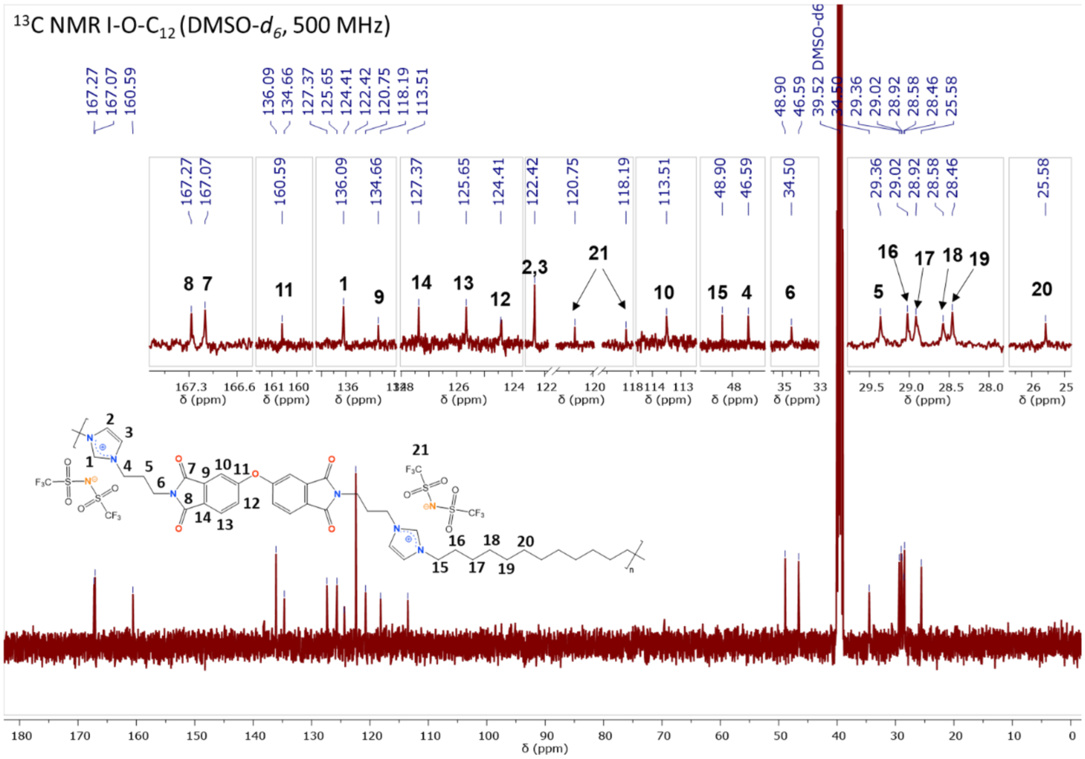
¹³C NMR I-O-C12 (DMSO-d6, 500 MHz)
39.52 DMSO-d6
δ (ppm)

^{13}C/Dept 45° NMR I-O-C_{12} (DMSO-d_6, 500 MHz)

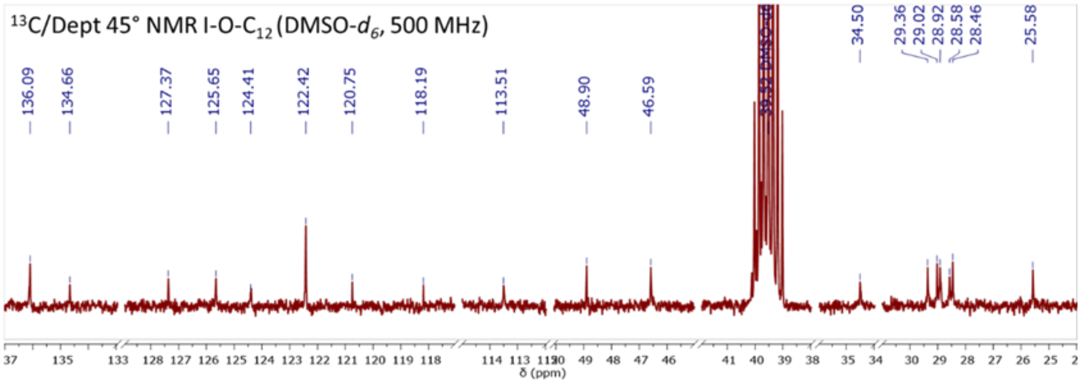

COSY NMR I-O-C_{12} (DMSO-d_6, 500 MHz)

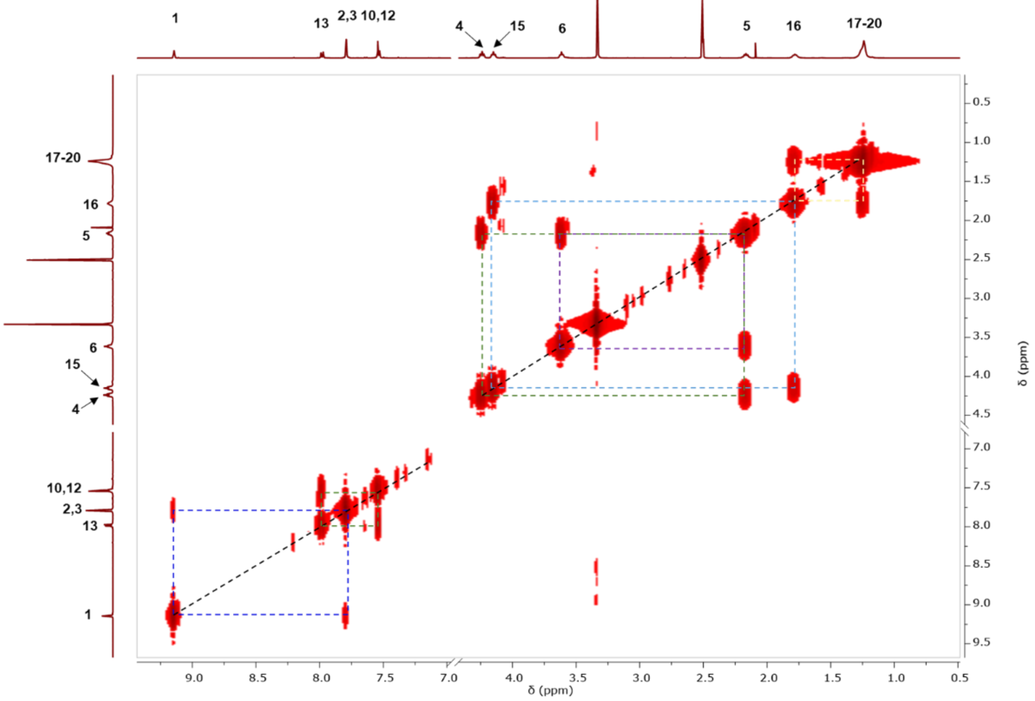

HSQC NMR I-O-C_{12} (DMSO-d_6, 500 MHz)

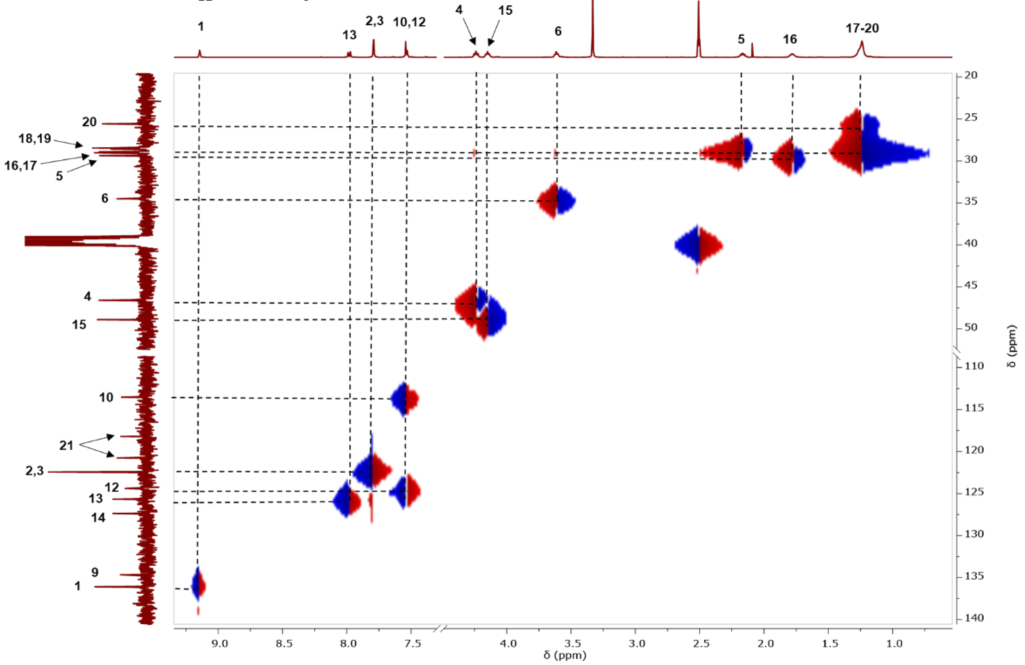

HMBC NMR I-O-C_{12} (DMSO-d_6, 500 MHz)

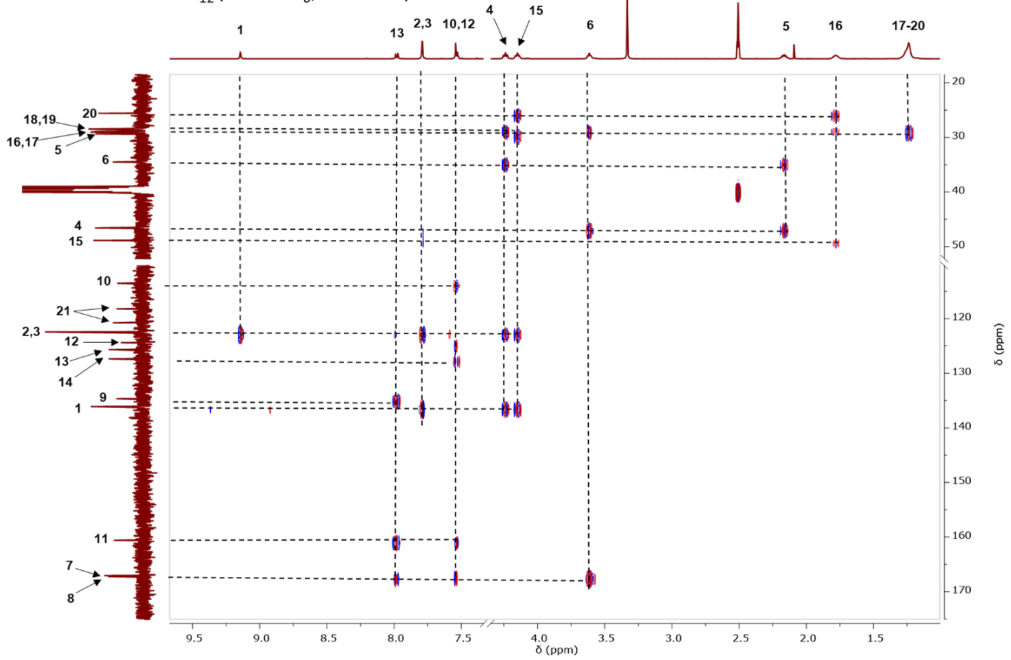

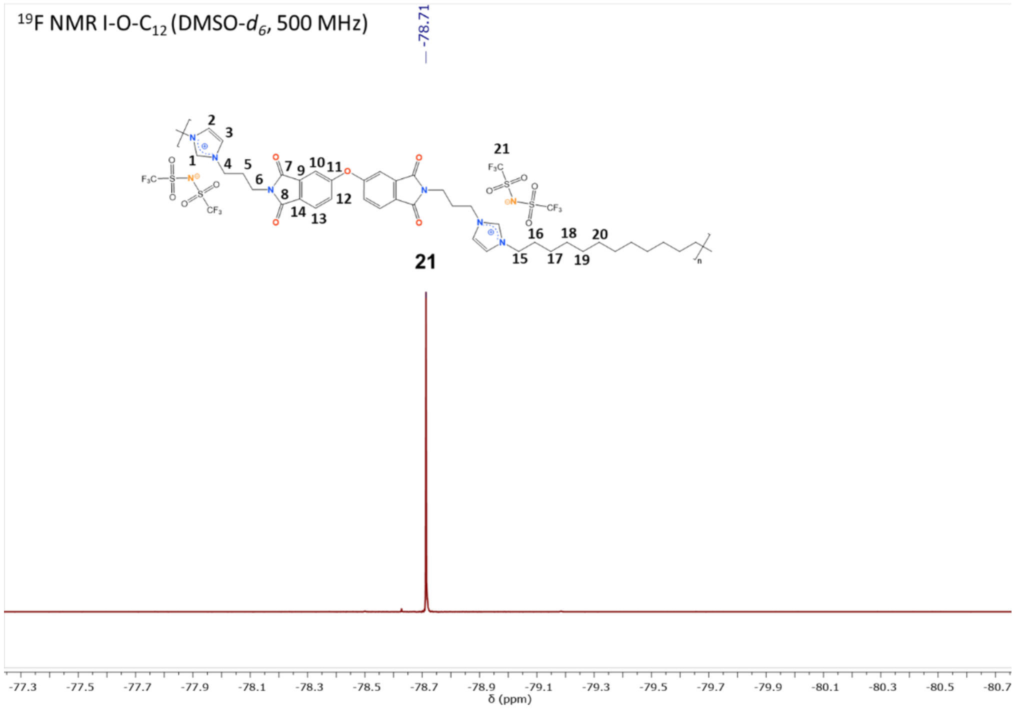

Figure S13: FT-IR-ATR and ^{1}H, ^{13}C, DEPT 45°, COSY, HSQC, HMBC and ^{19}F NMR spectra of ionene O-C_{12}.

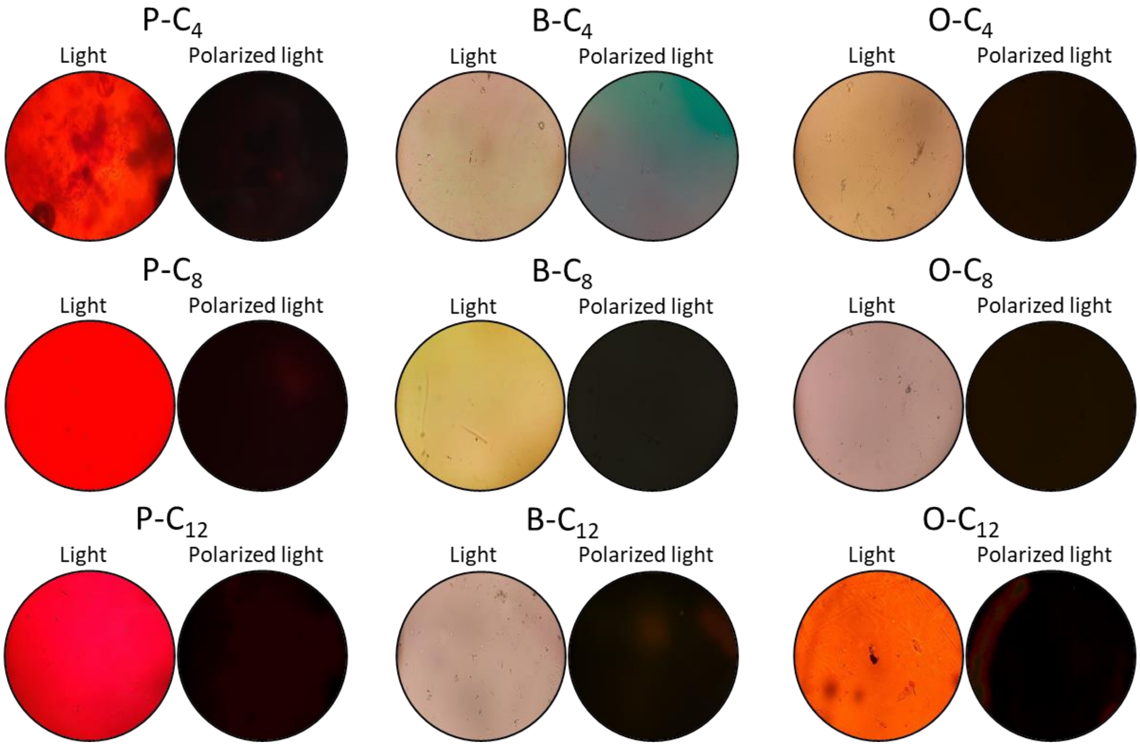

Figure S14: Evaluation of the crystalline structure of ionenes using polarised light microscopy.

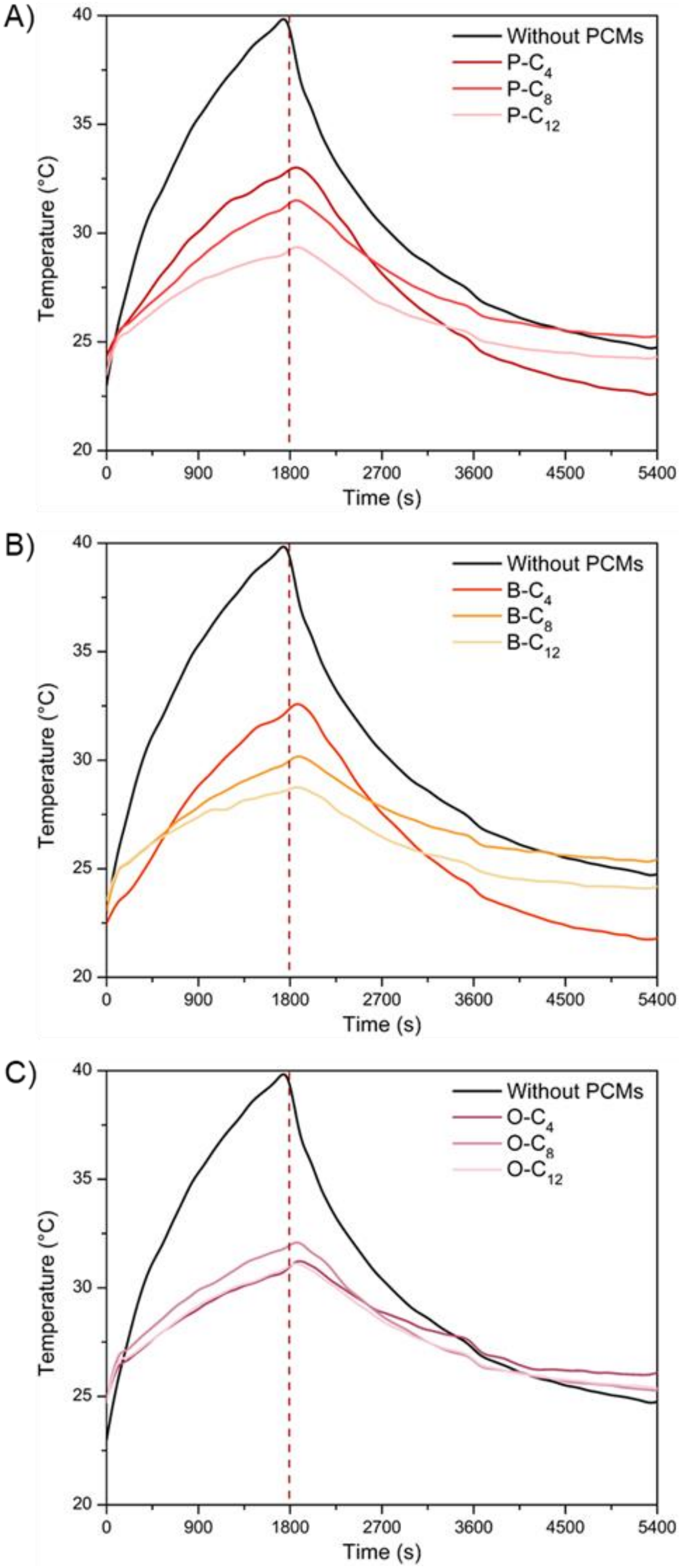

Figure S15: Results of the evaluation of the ionenes as SS-PCMs in heat dissipation.
